# Methylcellulose colony assay and single-cell micro-manipulation reveal progenitor-like cells in adult human pancreatic ducts

**DOI:** 10.1016/j.stemcr.2023.02.001

**Published:** 2023-03-02

**Authors:** Janine C. Quijano, Lena Wedeken, Jose A. Ortiz, Heather N. Zook, Jeanne M. LeBon, Angela Luo, Jeffrey Rawson, Jacob R. Tremblay, Jacob M. Mares, Kassandra Lopez, Min-Hsuan Chen, Kevin Jou, Carlos Mendez-Dorantes, Ismail H. Al-Abdullah, Debbie C. Thurmond, Fouad Kandeel, Arthur D. Riggs, Hsun Teresa Ku

**Affiliations:** 1 Department of Translational Research & Cellular Therapeutics, City of Hope, 1500 E. Duarte Road, Duarte, CA 91010, USA; 2 Irell and Manella Graduate School of Biological Sciences, City of Hope, Duarte, CA 91010, USA; 3 Integrative Genomics Core, City of Hope, Duarte, CA 91010, USA; 4 Department of Molecular & Cellular Endocrinology, City of Hope, Duarte, CA 91010, USA; 5 Department of Clinical Diabetes, Endocrinology & Metabolism, City of Hope, Duarte, CA 91010, USA; 6 Department of Diabetes & Drug Discovery, City of Hope, Duarte, CA 91010, USA

**Keywords:** colonies, self-renewal, differentiation, pancreatic colony-forming units, methylcellulose, 3D culture, insulin-producing beta cells, ROCK inhibitor, NOTCH signaling, single-cell RNA sequencing

## Abstract

Progenitor cells capable of self-renewal and differentiation in the adult human pancreas are an under-explored resource for regenerative medicine. Using micro-manipulation and three-dimensional colony assays we identify cells within the adult human exocrine pancreas that resemble progenitor cells. Exocrine tissues were dissociated into single cells and plated into a colony assay containing methylcellulose and 5% Matrigel. A subpopulation of ductal cells formed colonies containing differentiated ductal, acinar, and endocrine lineage cells, and expanded up to 300-fold with a ROCK inhibitor. When transplanted into diabetic mice, colonies pre-treated with a NOTCH inhibitor gave rise to insulin-expressing cells. Both colonies and primary human ducts contained cells that simultaneously express progenitor transcription factors SOX9, NKX6.1, and PDX1. In addition, *in silico* analysis identified progenitor-like cells within ductal clusters in a single-cell RNA sequencing dataset. Therefore, progenitor-like cells capable of self-renewal and tri-lineage differentiation either pre-exist in the adult human exocrine pancreas, or readily adapt in culture.

## Highlights

• Single cells from human pancreatic ducts form colonies in a 3D culture
• Ductal progenitor cells self-renew and differentiate to multiple lineages
• Transplanted colonies further differentiate toward insulin-expressing cells
• Single-cell RNA sequencing confirms some ductal cells have progenitor phenotypes

## Graphical abstract

**Figure:**
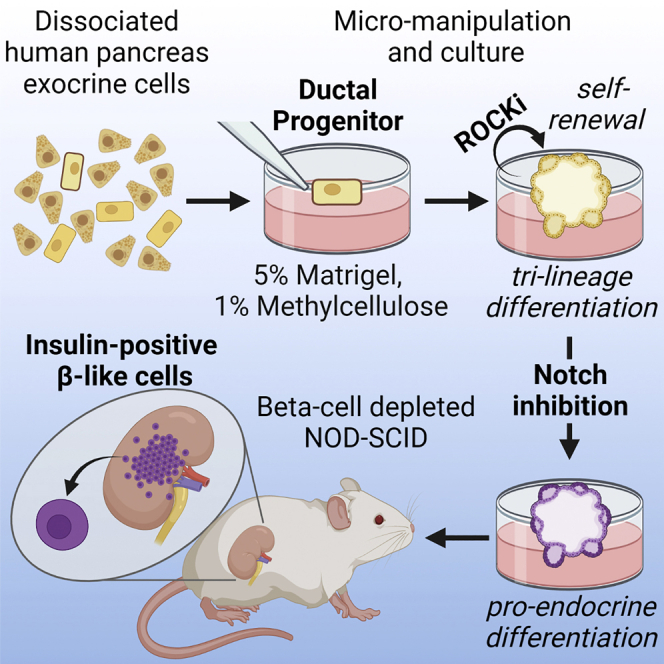

## Teaser

In this work, Quijano, Wedeken, and colleagues isolated and characterized progenitor-like cells from the ducts of adult human pancreas. These ductal cells self-renew and differentiate into multiple pancreatic lineages in culture. When transplanted into mice, these cells further differentiate into insulin-expressing cells essential for regulating blood glucose levels, thus suggesting a potential utility for clinical use.

## Introduction

Progenitor cells are distinguished by their ability to both self-renew and differentiate. These cells have been identified in many adult organs and can maintain tissue homeostasis and initiate repair of injuries. In the adult pancreas, there are three major cell lineages: ductal, acinar, and endocrine cells that include insulin-secreting beta cells. Studies of mouse embryos revealed that, in early (<E12.5) pancreas development, multi-potent progenitor cells (MPCs), expressing *Sox9*, *Pdx1*, and *Nkx6.1* (Gu et al., 2002; Kopp et al., 2011; Nelson et al., 2007), can give rise to these three lineages *in vivo* (Gu et al., 2002; Kopp et al., 2011) as well as *in vitro* using a three-dimensional (3D) culture assay (Greggio et al., 2013). Using *in vivo* lineage-tracing strategies, some studies found that in adult mice ductal cells can also give rise to beta cells (Al-Hasani et al., 2013; Dirice et al., 2019; Gribben et al., 2021; Inada et al., 2008; Xu et al., 2008), while others refuted these findings (Kopp et al., 2011; Solar et al., 2009; Zhao et al., 2021).

Although *in vivo* studies remain inconclusive, the use of certain 3D cultures has shown that some of the adult murine ductal cells self-renew and differentiate *in vitro* (Dorrell et al., 2014; Huch et al., 2013). For example, the 3D organoid assay established by Huch et al. (2013) showed that dissociated adult murine ductal cells and duct fragments can differentiate into endocrine and ductal, but not acinar, cell lineages in the presence of high concentrations of Matrigel. In contrast, the 3D colony assay system developed by our laboratory (Jin et al., 2013) uses methylcellulose, a biologically inert and viscous material, which allows us to lower Matrigel concentration to 5% v/v and detect tri-lineage differentiation. In a methylcellulose-containing semisolid medium, cells cannot move and aggregate. Following the tradition of hematologists who call hematopoietic progenitor cells grown in a methylcellulose-containing culture medium “colony-forming units,” we named a pancreatic progenitor cell capable of giving rise to a colony a pancreatic colony-forming unit (PCFU). Using this system, quantifying colony-forming progenitor cells can be done with relative ease.

In cadaveric human pancreatic ducts, previous reports have identified progenitor-like cells that are capable of duct and endocrine differentiation and some expansion (Bonner-Weir et al., 2000; Georgakopoulos et al., 2020; Lee et al., 2013; Loomans et al., 2018; Qadir et al., 2018). However, no human study of pancreas tissue thus far has utilized micro-manipulation of a single cell or colony to address lineage potential or lineage composition, respectively. Micro-manipulation is a technique that utilizes tools such as a pipette with a narrow opening to aspirate a cell or a colony of interest, one at a time, for subsequent downstream analysis (Tremblay et al., 2016). Such a clonal analysis is critical to ascertain multi-potency because a population of uni-potent progenitor cells for different lineages may collectively appear to be multipotent. Thus, despite the advances made in the aforementioned studies, no definitive evidence exists yet to demonstrate self-renewal and tri-lineage differentiation of adult human pancreatic progenitor cells. In this study, we describe a human colony assay system that reveals the self-renewal and tri-lineage differentiation abilities of an adult human ductal subpopulation. Single-cell RNA sequencing (scRNA-seq) analysis on dissociated exocrine cells confirms ductal cell heterogeneity, with a sub-cluster expressing genes consistent with progenitor cell phenotype.

## Results

### Establishment of a methylcellulose-based colony assay for adult human PCFUs

**Figure 1:**
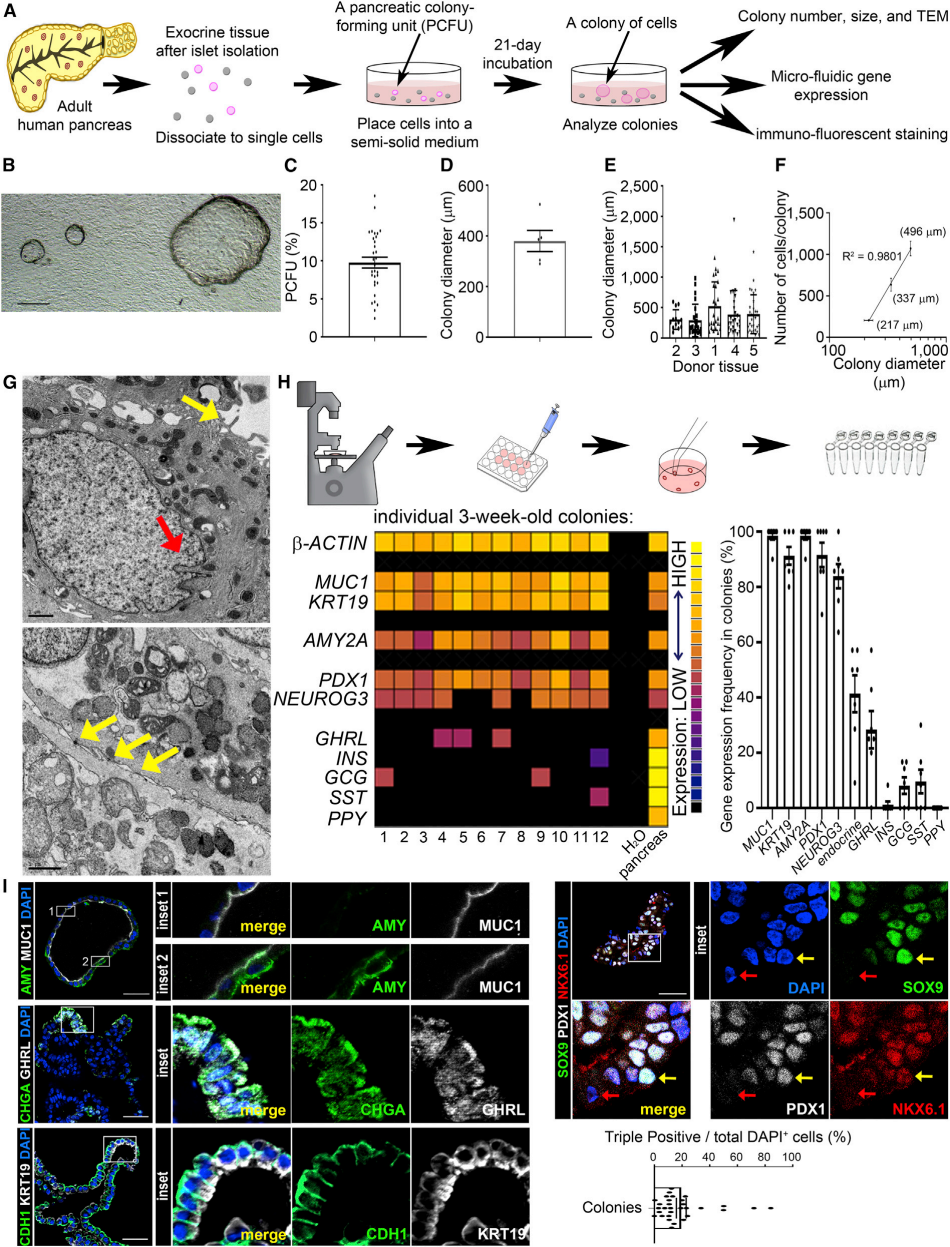
Methylcellulose-based colony assay for adult human pancreatic progenitor cells capable of tri-lineage differentiation (A) Experimental diagram. (B) Representative bright-field image of colonies. Scale bar, 200 μm. (C) % PCFUs in dissociated exocrine tissues is 9.8% ± 0.7% (N = 31 donors). (D) Colony diameter = 394 ± 37 μm; mean ± SEM, ≥10 colonies per donor, N = 6 donors with 4 technical replicates. (E) Diameters of colonies between different donors (N = 5). (F) Mean diameter of colonies is positively correlated with the total number of cells per colony (R^2^ = 0.9801); mean ± SEM from 2 independent experiments and 20 individual colonies per data point. (G) TEM of 3-wo colonies displaying microvilli on the apical side (top, yellow arrow), nuclear invaginations (top, red arrow), and desmosomes (bottom, yellow arrows). Scale bars, 1 μm. (H) Micro-manipulation of individual colony for microfluidic qRT-PCR. Representative heatmap of lineage markers; n = 58 colonies, N = 7 donors. Gene expression frequency; mean ± SD. (I) IF staining confirms protein expression. Scale bar, 50 μm (insets enlarged 4×). Yellow arrow points to a representative cell that is triple-positive (TP) for SOX9, PDX1, and NKX6.1 and a red arrow for a non-TP cell. TP quantification represents mean ± SEM (19.5% ± 3.5%) from a total of 31 colonies from N = 3 donors. See also Figure S1B.

We studied pancreatic exocrine tissues, which include ductal and acinar cells, from 41 cadaveric human donors without apparent disease (Table S1). These donors had an average age of 36 ± 14 years, body mass index of 30.4 ± 6.6 kg/m^2^, and hemoglobin A1c of 5.1 ± 0.3% (Table S2). After islets were isolated, the pancreas tissue was dissociated into a single-cell suspension and was either cryopreserved or immediately plated into our colony assay system (Figure 1A). Our “standard” culture medium (Table S3) contains methylcellulose (1% w/v), non-defined extracellular matrix proteins (Matrigel; 5% v/v) (Jin et al., 2013), and defined soluble factors (Nicotinamide, EGF, Noggin, Exendin4, SB202190, Gastrin, RSPO1, VEGF, and A83-01) that were inspired by culture conditions for adult human gastrointestinal stem cells (Bartfeld et al., 2015; Sato et al., 2011) and adult murine ductal progenitor cells (Huch et al., 2013; Wedeken et al., 2017). Using this colony assay system, we achieved 100% isolation efficiency from every human exocrine tissue obtained to date, in contrast to 75%–80% shown by others (Boj et al., 2015).

After 3 weeks of culture morphologically distinct colonies formed, mostly appearing as hollow spheres (Figure 1B). The % PCFU, or colony-forming efficiency, varied among different donors with an average of 9.8% ± 0.7% (n = 31; range 2.4%–18.6%) (Figure 1C). The mean diameter of a colony was 380 ± 42 μm (Figure 1D), with individual donors showing high variability of sizes (Figure 1E). Colonies were segregated into small, medium, and large categories (10 colonies each) and were dispersed into single cells to quantify the number of cells per category. We observed a strong positive correlation between colony size and cell number (Figure 1F; R^2^ = 0.98), indicating that colony size is predictive of the number of cells in that colony. The colony size can be indicative of the proliferative potential of the originating PCFU. Alternatively, the differences in colony size may be due to variable delay of cells entering replication or time needed for differentiation.

Transmission electron microscopy (TEM) revealed that cells in colonies displayed microvilli facing the lumen (Figure 1G), suggesting apical polarization. Cells had nuclear invaginations and desmosomes at cell-cell junctions. These results indicate that a colony is composed of duct-like cells. Furthermore, 3D scanning electron microscopy (3D-SEM) analysis clarified that the walls of the colonies contained individual cells that were flat and elongated (Video S1). Also, microvilli were facing lumen and nuclei contained invaginations (Figure S1C); confirming that most cells in a colony are ductal and exhibit apical-basal polarity.

Video S1. Cells in a 3-week-old colony, grown from dissociated pancreatic exocrine cells (unsorted) in the standard colony assay, are arranged mostly as a single-cell layer Serial block-face scanning electron microscopy (SBF-SEM), also known as 3D-SEM, was used to generate 500 serial images of a colony. Using Amira software, the images were stitched sequentially and presented as a movie. The movie starts by scanning through the cells from the top of the stacked images to the bottom, then scans from the bottom of the stacked images to the top. Next, the borders (including microvilli) of individual cells (n = 11) were segmented; each cell was painted a unique color. Translucent colors were rendered initially and then changed to solid colors to increase visualization of the arrangement of cells. Note a smooth side of the cell surface, which is facing away from the lumen and is adjacent to the methylcellulose and Matrigel-containing semisolid medium. The yellow cell has an elongated shape with cytoplasmic protrusions.

The lineage potential of a PCFU is reflected in colonies expressing markers for various lineages. To determine the lineage potential of each PCFU, we micro-manipulated each 3-week-old (3-wo) colony by identifying it under a light microscope, picking the colony up with a pipette and placing that volume into a microcentrifuge tube (Figure 1H) pre-loaded with reagents for microfluidic qRT-PCR analysis (Jin et al., 2013). All individual colonies expressed high levels of markers for ductal (*MUC1*, *KRT19*, *KRT7*, *HNF1B*, *SOX9*, and *PROM1*), and multi-potent and endocrine progenitor cells (*PDX1*, *NEUROG3*, *NEUROD2*, *MAFB*, and *NKX6.1*; Figures 1H and S1A). The acinar cell marker *AMY2A* was also consistently expressed by all colonies. In contrast, the frequency of colonies that displayed at least one of the combined five endocrine markers (*INS*, *GCG*, *PPY*, *SST*, and *GHRL*) was only 41.7% ± 15.5%, with ghrelin being the most frequent (30.6% ± 16.9%). Because all colonies expressed markers for ductal and acinar lineages, and 41.7% of colonies expressed combined endocrine markers, these results demonstrate that approximately 40% of adult human PCFUs are tri-potent. The lower expression of *INS* in colonies at this stage reflects suboptimal culture conditions rather than a lack of lineage potential, as is shown later in the transplantation study.

Immunofluorescence (IF) staining verified protein expression of MUCIN1, KRT19, amylase, ghrelin, chromogranin A (a pan-endocrine marker), and CDH1 (a pan-epithelial marker) in 3-wo colonies (Figures 1I and S1B). MUCIN1 was detected at the surface of cells facing the lumen, confirming apical polarization. Because the transcription factors PDX1, SOX9, and NKX6.1 are known markers for the mouse and human embryonic MPCs (Jennings et al., 2013), we co-stained for these markers. On average, one-fifth of the cells in 3-wo colonies were triple-positive (TP) for SOX9^+^/PDX1^+^/NKX6.1^+^ (Figure 1I, 19.5% ± 3.5%), demonstrating that a subset of cells within colonies display a progenitor cell phenotype.

### A micro-manipulated single PCFU is sufficient to give rise to a 3-wo colony expressing the three major pancreatic lineages

**Figure 2:**
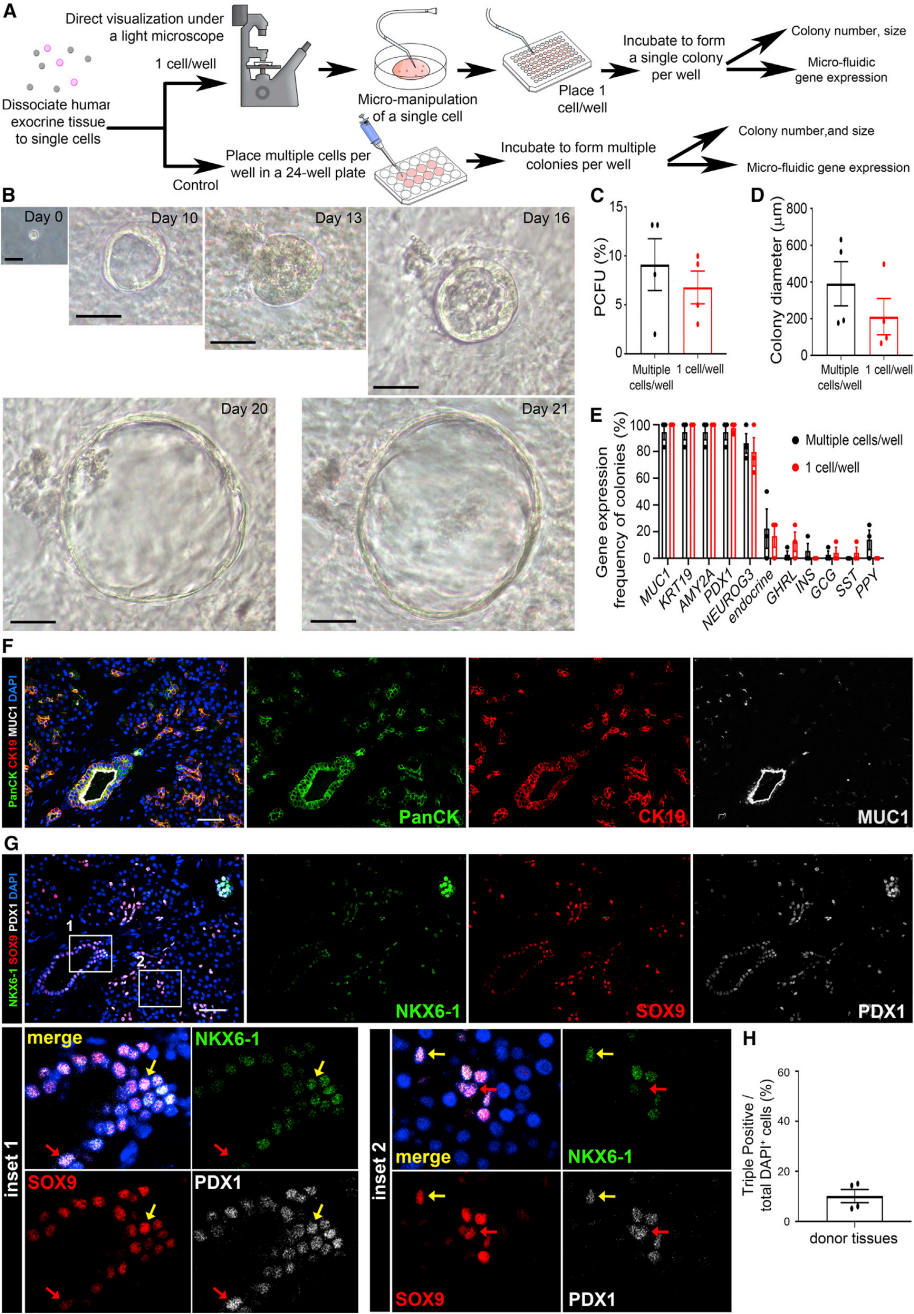
A micro-manipulated single PCFU is sufficient to give rise to a colony expressing the three major pancreas lineages, and identification of SOX9^+^/PDX1^+^/NKX6.1^+^ cells in endogenous ducts (A) Experimental diagram. (B) Time course bright-field imaging of a single PCFU grown into a colony. Scale bar, 20 μm (on day 0) and 50 μm (for all other days). (C and D) (C) % PCFUs and (D) mean diameter of colonies grown from 1 cell per well vs. multiple cells per well; mean ± SEM from 4 independent experiments using 3 donor tissues. Paired t test determined significance. (E) Microfluidic qRT-PCR analysis of colonies grown from unsorted cells plated at multiple cells per well (black) (n = 24 colonies, 3 independent experiments from 2 donors), vs. 1 cell per well (red) (n = 22 colonies); mean ± SD. Significance was determined by two-way ANOVA with Sidak’s multiple comparison. (F) IF staining of Pan-CK (green), CK19 (red), and MUC1 (white) in human pancreas. The image contains interlobular, intralobular, and intercalated ducts. Scale bar, 50 μm. (G) The same region in a sequential slide to (F) is stained with NKX6.1 (green), PDX1 (white), and SOX9 (red). Insets 1 and 2 highlight an interlobular and an intercalated duct, respectively; both contained TP cells (yellow arrows point to representatives of SOX9^+^PDX1^+^NKX6.1^+^ cells) and non-TP cells (red arrow points to representative of a SOX9^+^PDX1^+^NKX6.1^−^ cell). Scale bar, 50 μm (insets enlarged 4×). (H) Quantification of % TP cells among total cells within stitched images; mean ± SEM (10.2% ± 2.6%), N = 4 donor tissues. See also Figures S1F and S2.

To further ascertain the tri-lineage potential of PCFUs, we micro-manipulated freshly dissociated cells before culture by identifying single cells under a microscope and placing them into a 96-well plate at 1 cell per well (Figure 2A). Cells from the same donor were also plated into a standard colony assay as a control. Tracking each well of the 96-well plate for 3 weeks confirmed that a colony originated from one cell (Figures 2B and S1D). Compared with the control colonies, colonies derived from micro-manipulated single cells showed no significant difference in % PCFU (Figure 2C) and diameter (Figure 2D), suggesting that the formation of a colony is cell autonomous.

Microfluidic qRT-PCR analysis on individual 3-wo colonies demonstrated that the frequency of colonies expressing tri-lineage markers was similar between colonies derived from plating with single vs. multiple cells per well (Figures 2E and S1E). Overall, a total of 306 micro-manipulated single cells from 3 independent experiments resulted in an average of 6.0 ± 2.1% colony formation. Of those colonies, more than 15% gave rise to 3-wo colonies expressing duct, acinar, and endocrine lineage markers (example colonies nos. 3 and 7 in Figure S1E), confirming the tri-potency of those PCFUs.

### Endogenous ducts contain TP cells

The presence of TP cells in 3-wo colonies (Figure 1I) and the tri-potency of individually micro-manipulated PCFUs (Figure 2E) prompted us to examine the existence of TP cells in the adult human pancreas. Large and small ducts were identified by pan-CK, CK19, and MUC1 staining (Figures 2F and S2A). In the sequential slide, TP cells were identified in ductal areas (Figure 2G, yellow arrows; Figures S1F andS2B). Some SOX9^+^/PDX1^+^ ductal cells lacked NKX6.1 (Figure 2G, red arrows), suggesting heterogeneity. Overall, the TP cells constituted 10.2 ± 2.6% of total pancreatic cells (Figure 2H), a frequency consistent to % PCFUs among dissociated exocrine cells (Figure 1C).

### Live-sorted human pancreatic CD133^+^CD49f^low^ cells are enriched for ductal cells

**Figure 3:**
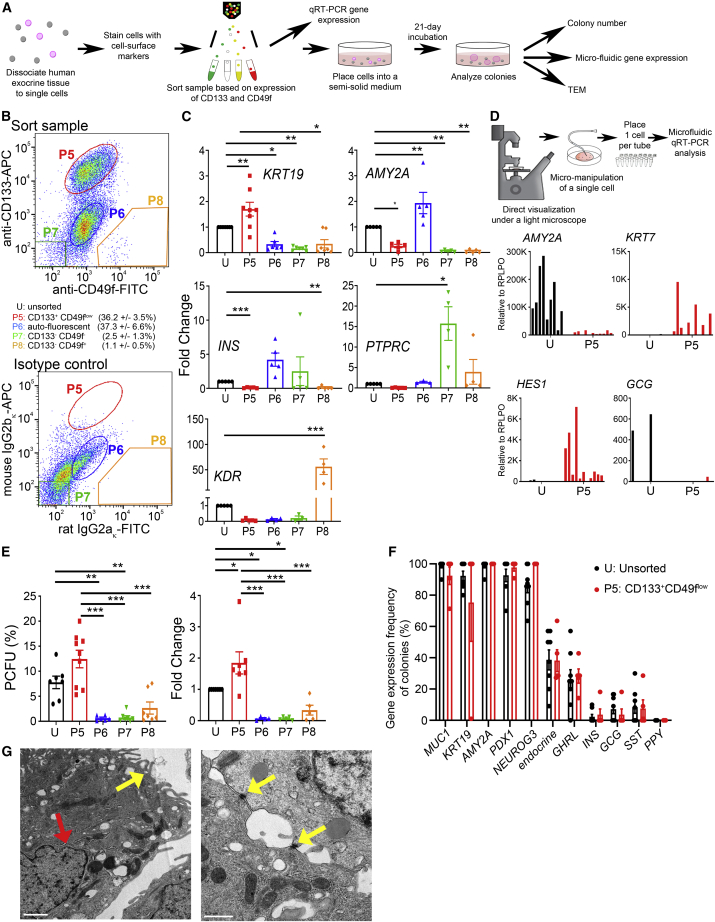
Live-sorted human pancreatic CD133^+^CD49f^low^ ductal cells are enriched for PCFUs (A) Experimental diagram. (B) Representative sorting windows for four cell populations (P5, P6, P7, and P8) and their percentages; mean ± SEM, N = 5 donors. (C) qRT-PCR analysis of freshly sorted populations, compared with gene expression levels of the unsorted population (U) as fold change for ductal (*KRT19*, N = 6 donors), acinar (*AMY2A*, N = 5 donors), endocrine (*INS*, N = 5), endothelial (*KDR*, N = 3 donors), and leukocyte (*PTPRC*, N = 3 donors) cells; mean ± SEM. (D) Micro-manipulation of freshly sorted individual cells for microfluidic qRT-PCR analysis. Data are from N = 1 donor with n ≥ 11 single cells per population. (E) PCFU among sorted populations compared with unsorted (U) population, expressed as % PCFU (left) or fold change (right) (N = 7 donors; mean ± SEM). (F) Gene expression frequencies of colonies grown from unsorted (U, black) or sorted (P5, red) cells. U: n = 32 colonies, N = 4 donors. P5: n = 38 colonies, N = 5 donors, mean ± SD. Significance was determined by two-way ANOVA, with Sidak’s multiple comparison. (G) Ultrastructure analysis of P5-derived colonies displaying microvilli on the apical side (left, yellow arrow), nucleus invagination (left, red arrow), and desmosomes (right, yellow arrows). Scale bar, 1 μm (left) or 0.5 μm (right). ∗p < 0.05, ∗∗p < 0.01, ∗∗∗p < 0.001. See also Figures S3A–S3E.

CD133 (*PROM1*) is a known ductal marker, and CD49f (*ITGA6*) co-expresses with CD133 in human fetal pancreas (Sugiyama et al., 2007). We therefore tested the utility of these cell surface markers in fluorescence-activated cell sorting (Figure 3A). Freshly dissociated exocrine cells were stained with antibodies against CD133 and CD49f and analyzed using flow cytometry (Figures 3B and S3A). All CD133^+^ cells expressed low levels of CD49f, indicating that CD49f did not improve adult ductal cell identification, but CD49f separated a CD133^−^ (non-ductal) subpopulation (see population 8 [P8] below). The CD133^+^CD49f^low^ cells (marked as P5) comprised 36.2 ± 3.5% of total dissociated exocrine cells (Figure 3B). We next sorted four subpopulations: CD133^+^CD49f^low^ (P5), CD133^low^CD49f^low^ (P6), CD133^−^CD49f^−^ (P7), and CD133^−^CD49f^+^ (P8). Conventional qRT-PCR analyses revealed that, compared with unsorted cells (U), freshly sorted P5 cells expressed higher levels of the ductal marker *KRT19* and low-to-undetectable levels of endocrine (*INS*) and acinar (*AMY2A*) markers (Figure 3C). Markers for leukocytes (*CD45*) and endothelial cells (*KDR*) were also expressed lower in P5 cells compared with unsorted cells (Figure 3C). Microfluidic qRT-PCR confirmed that freshly sorted, micro-manipulated single P5 cells expressed *KRT7* and low levels of *AMY2A* (Figure 3D). Although most of the islets were already removed, infrequent *GCG* expressing cells remained in the unsorted population (Figure 3D). These results confirm that CD133^+^CD49f^low^ cells are specifically enriched for ductal cells.

### Sorted human pancreatic CD133^+^CD49f^low^ ductal cells are enriched for PCFUs

To assess which pancreatic subpopulation(s) were enriched for PCFUs, sorted cells were plated in our standard colony assay. Unsorted cells from this cohort displayed an overall % PCFU of 7.8 ± 1.3% (Figure 3E, left). Compared with unsorted cells, only P5 displayed a higher (1.9- ± 0.4-fold) % PCFU (Figure 3E, right). The 3-wo colonies grown from P5 cells also appeared as hollow spheres (Figure S3B), with no donor-to-donor variation in colony diameter (Figure S3C). The mean diameter of colonies grown from P5 cells was 316 ± 34 μm (Figure S3D), which is comparable to that of colonies grown from unsorted cells (compare Figure S3D with 1D; p > 0.05). Gene expression patterns and frequencies of P5-derived individual colonies were similar to those derived from unsorted cells (Figures 3F and S3E), suggesting that P5-derived PCFUs are tri-potent. Finally, TEM of colonies grown from P5 cells displayed microvilli facing the lumen, nuclei with invaginations, and desmosomes at cell-cell junctions (Figure 3G). Overall, these results demonstrate that sorting does not significantly impact the growth, differentiation, or colony morphology of human PCFUs, and that PCFUs are derived from the ducts. Due to the unchanged colony phenotypes and logistic limitation with cadaveric tissues, unsorted cells were used for subsequent experiments.

### Adult human PCFUs self-renew and expand up to 300-fold over 9 weeks in the presence of a ROCK inhibitor

**Figure 4:**
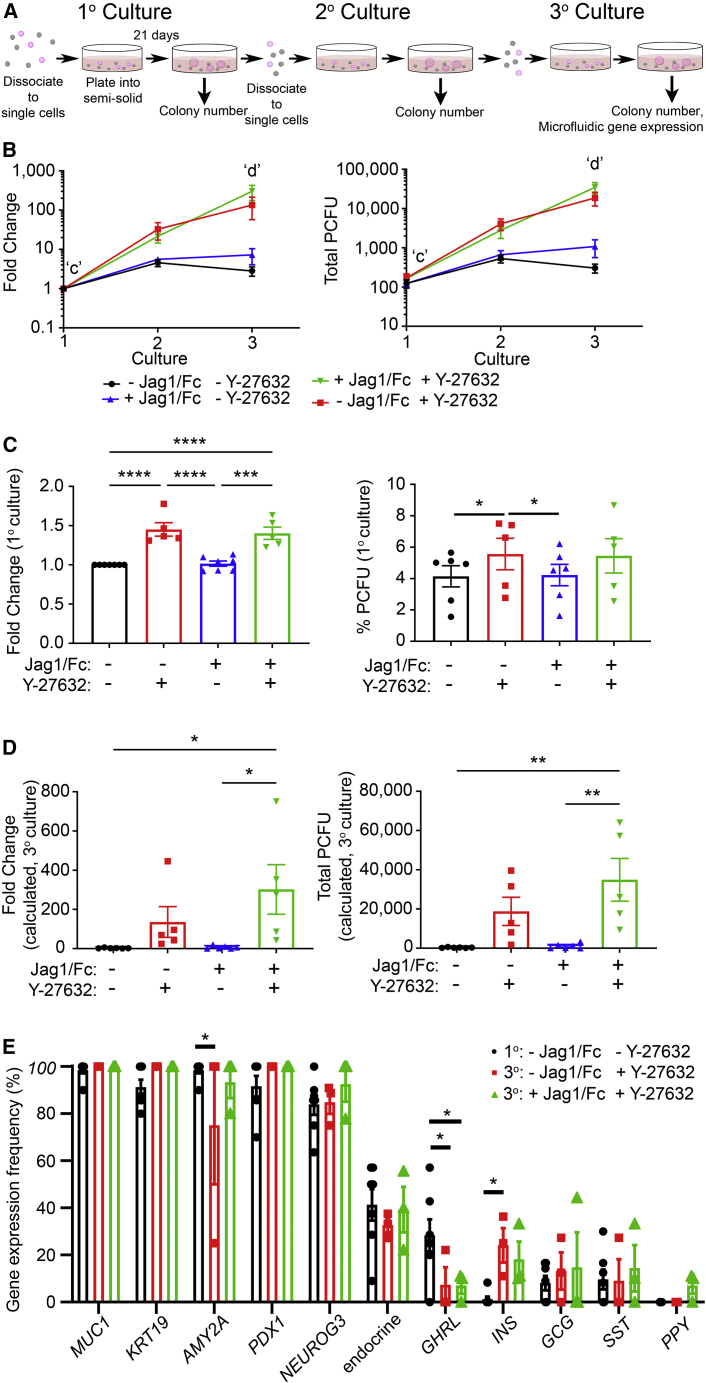
Adult human PCFUs self-renew and expand up to 300-fold (A) Experimental diagram. (B) PCFU fold change (left) and total PCFU (right); mean ± SEM, N = 4 donors from 5 (groups containing Y-27632 ± Jag1/Fc) or 6 (control and Jag1/Fc alone) independent experiments, with 4 technical replicates per plating. (C and D) Further analysis of data from (B); PCFUs in the 1° culture (C) or on the 3° culture (D). (E) Microfluidic qRT-PCR analysis of n ≥ 8 individually handpicked colonies per donor collected from the 3° culture, grown in the presence of Y-27632 (red; n = 28 total colonies from N = 3 donors) or Y-27632 and Jag1/Fc (green; n = 28 total colonies from N = 3 donors). Black: same data as Figure 1H; mean ± SD. Significance was determined by two-way ANOVA, with Tukey’s multiple comparison. ∗p < 0.05, ∗∗p < 0.01, ∗∗∗p < 0.001, ∗∗∗∗p < 0.0001. See also Figures S3F–S3H.

We assessed the self-renewal abilities of PCFUs using serial dissociation of colonies into single cells and re-plating from 1° through 3° cultures (Figure 4A). After 9 weeks, the number of PCFUs only expanded about 3-fold in our standard culture (Figures 4B and 4D). Prior studies found that inhibition of Rho-associated protein kinase (ROCK) enhances the survival of fetal murine pancreatic progenitor cells *in vitro* (Greggio et al., 2013), and that Notch activation is required for maintaining the progenitor cell pool (Apelqvist et al., 1999). We therefore tested the effects of Y-27632, a ROCK inhibitor, and Jag1/Fc, a Notch activator, on PCFU self-renewal. Compared with the control, addition of Y-27632 with or without Jag1/Fc increased the number of PCFUs over 9 weeks by 302- ± 126-fold or 136- ± 78-fold, respectively (Figure 4B). Y-27632 treatment also increased 1° colonies (Figure 4C), indicating enhanced PCFU survival. In contrast, Jag1/Fc alone did not affect PCFU self-renewal (Figures 4B and 4D) or survival (Figure 4C), even though Jag1/Fc could increase *MKI67* expression in colonies at days 14 and 21 (Figure S3F). In the 3° culture, % PCFUs among total cells plated with Y-27632 was significantly higher than the no addition control (Figure S3G), suggesting PCFU exhaustion over time without ROCK inhibition. These results demonstrate that ROCK inhibition, but not Notch activation, is sufficient for self-renewal of human PCFUs.

To determine whether PCFU tri-potency was preserved after expansion, individual 3° colonies from three donors were micro-manipulated and analyzed using microfluidic qRT-PCR. Only 3° colonies cultured with Y-27632, with or without Jag1/Fc, were analyzed. Similar to 1° colonies (Figure 1H), 3° colonies collectively expressed the three main pancreatic lineage markers (Figures 4E and S3H). There was no difference in the frequency of 3° colonies expressing pancreas lineage markers between colonies grown with Y-27632 and with the combination of Y-27632 + Jag1/Fc (Figure 4E). Interestingly, when comparing the 3° colonies grown with Y-27632 to 1° colonies grown in our standard culture, there was a reduction in the frequency of colonies expressing *AMY2A* and *GHRL*, but an increase of *INS* (Figure 4E). Overall, these data demonstrate that tri-potent PCFUs are preserved over 9 weeks in culture.

### Notch inhibition enhances endocrine progenitor gene expression profiles in human colonies

Many micro-manipulated individual CD133^+^CD49f^low^ cells expressed *HES1* (Figure 3D), a known Notch target gene. The relative low expression of endocrine genes in colonies grown in our standard culture (Figure 1H) prompted us to test Notch inhibition, because the reduction of HES1 can de-repress *NEUROG3* gene expression (Lee et al., 2001; Shih et al., 2012), which is necessary for endocrine lineage commitment (Apelqvist et al., 1999; Jensen et al., 2000; Murtaugh et al., 2003).

**Figure 5:**
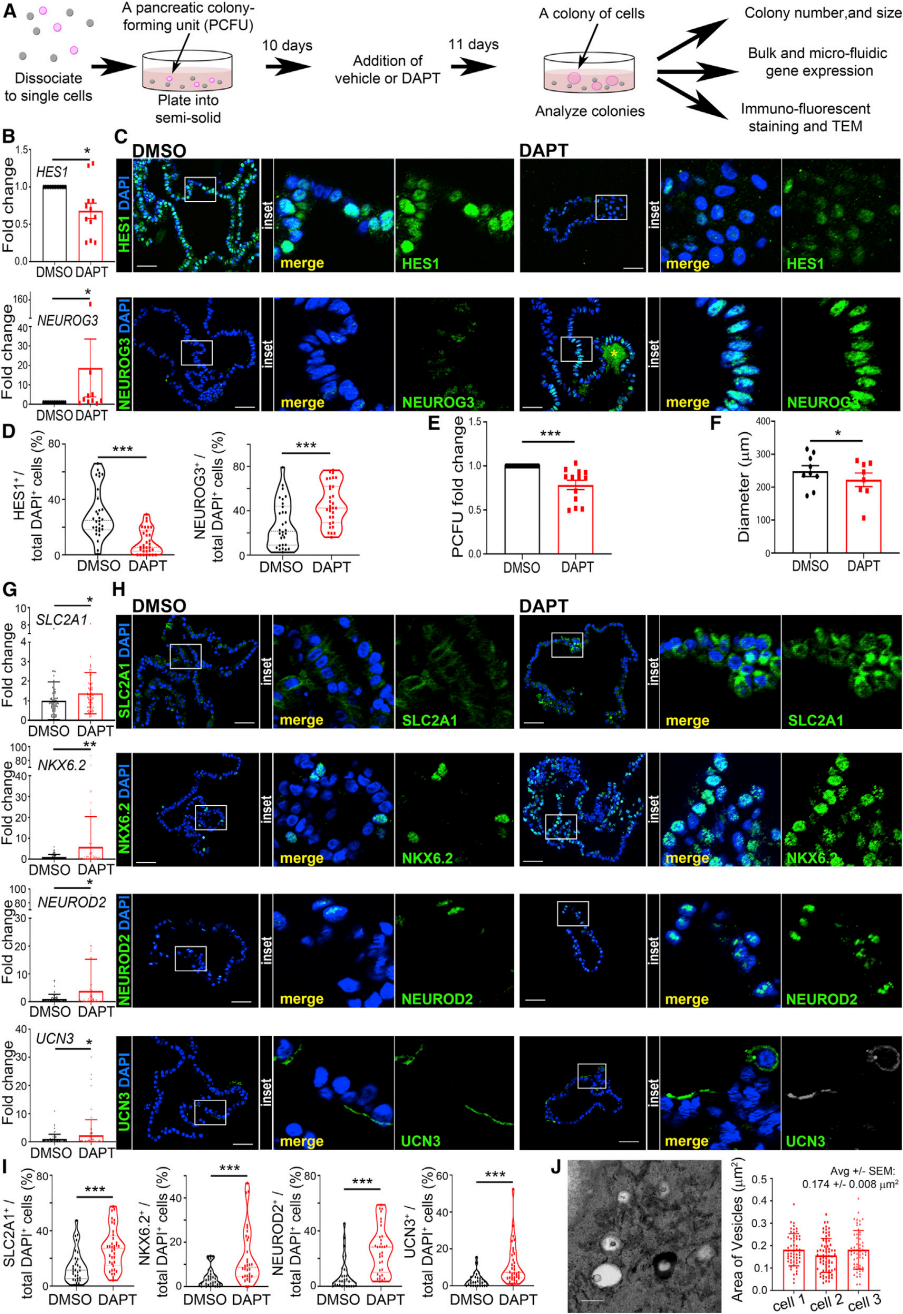
Notch inhibition enhances endocrine gene expression in human colonies (A) Experimental diagram. (B) Colonies analyzed for *HES1* and *NEUROG3* gene expression by qRT-PCR; mean ± SEM, n = 13 experiments, N = 9 donors. (C) IF of colonies treated with DMSO (left) or DAPT (right) with HES1 (green, top) or NEUROG3 (green, bottom) and DAPI (blue). Yellow star (^∗^, bottom-right) denotes an area of non-specific staining. Scale bar, 50 μm (box enlarged 4× to the right). (D) IF quantification of percent positive cells. n = 30–31 colonies treated with DMSO or DAPT from N = 3 donors. Each dot represents a colony. (E) % PCFU (fold change); mean ± SEM, N = 13 donors. (F) Average colony diameter; mean ± SEM, N = 8 donors. (G) Fold change of gene expression from individual colonies examined by microfluidic qRT-PCR analysis; mean ± SD, n = 73–78 colonies treated with DMSO or DAPT, N = 5 donors. Each dot represents a colony. (H) IF analysis of endocrine markers in DMSO-treated colonies (left) or DAPT (right). Scale bar, 50 μm (insets enlarged 4× to the right). (I) IF quantification of percent positive cells; mean ± SD, n = 30 DMSO and n = 34 DAPT, N = 3 donors. Each dot represents a colony. (J) DAPT-treated colonies were analyzed using TEM and three-dimensional scanning electron microscopy (3D-SEM). Left: a representative TEM photomicrograph of a portion of a cell showing vesicles with granules. Scale bar, 500 nm. Right: the area of the vesicles containing insulin-like granules were measured in 3 cells. Data represent areas from 55, 67, and 56 individual vesicles from cells 1, 2, and 3, respectively. ∗p < 0.05, ∗∗p < 0.01, ∗∗∗p < 0.001. See also Figure S4.

DAPT is a small molecule that inhibits gamma-secretase and is known to reduce *HES1* expression levels (Kopinke et al., 2011). It is known that the timing of Notch inhibition is critical for proper endocrine differentiation *in vivo* (Cras-Meneur et al., 2009) and *in vitro* (Shih et al., 2012; Wedeken et al., 2017). Addition of DAPT to human colonies on day 10 (Figure 5A) decreased *HES1* and increased *NEUROG3* expression compared with the vehicle control (Figure 5B). Quantification of IF staining confirmed a reduction of HES1^+^ cells and an increase of NEUROG3^+^ cells in DAPT-treated colonies (Figures 5C, 5D, and S4A). DAPT treatment reduced % PCFU (Figure 5E) and colony size (Figure 5F), suggesting that Notch signaling is necessary for the survival and growth of PCFUs, similar to behavior from pancreatic progenitors (Apelqvist et al., 1999).

Microfluidic qRT-PCR analysis of individual colonies revealed that DAPT did not increase the frequency of tri-lineage colonies (Figure S4B) but increased the expression of several beta cell maturation markers, including *SLC2A1*, *NKX6.2*, *NEUROD2*, and *UCN3* (Figure 5G). IF staining and quantification confirmed that an increased proportion of cells within DAPT-treated colonies express these maturation markers (Figures 5H, 5I, and S4C). These results demonstrate that Notch inhibition directs differentiation toward an endocrine phenotype in our colonies but does not change a PCFU from bi- to tri-potent.

To clarify if DAPT induced the formation of insulin vesicles, we performed TEM analysis of DAPT-treated colonies and found condensed insulin granules in vesicles (Figure 5J), which were not observed in control colonies. Using 3D-SEM, we analyzed 178 non-overlapping insulin vesicles from 3 cells and found that the mean area was 0.17 ± 0.01 μm^2^/vesicle (Figure 5J). The vesicles in DAPT-treated cells were slightly smaller than reported endogenous insulin vesicles (0.19 μm^2^/vesicle) (Fava et al., 2012). Also, the insulin granules appeared faint compared with adult beta cells, suggesting functional immaturity (Ni et al., 2017).

### DAPT-treated human colonies give rise to insulin-expressing cells in hyperglycemic mice

**Figure 6:**
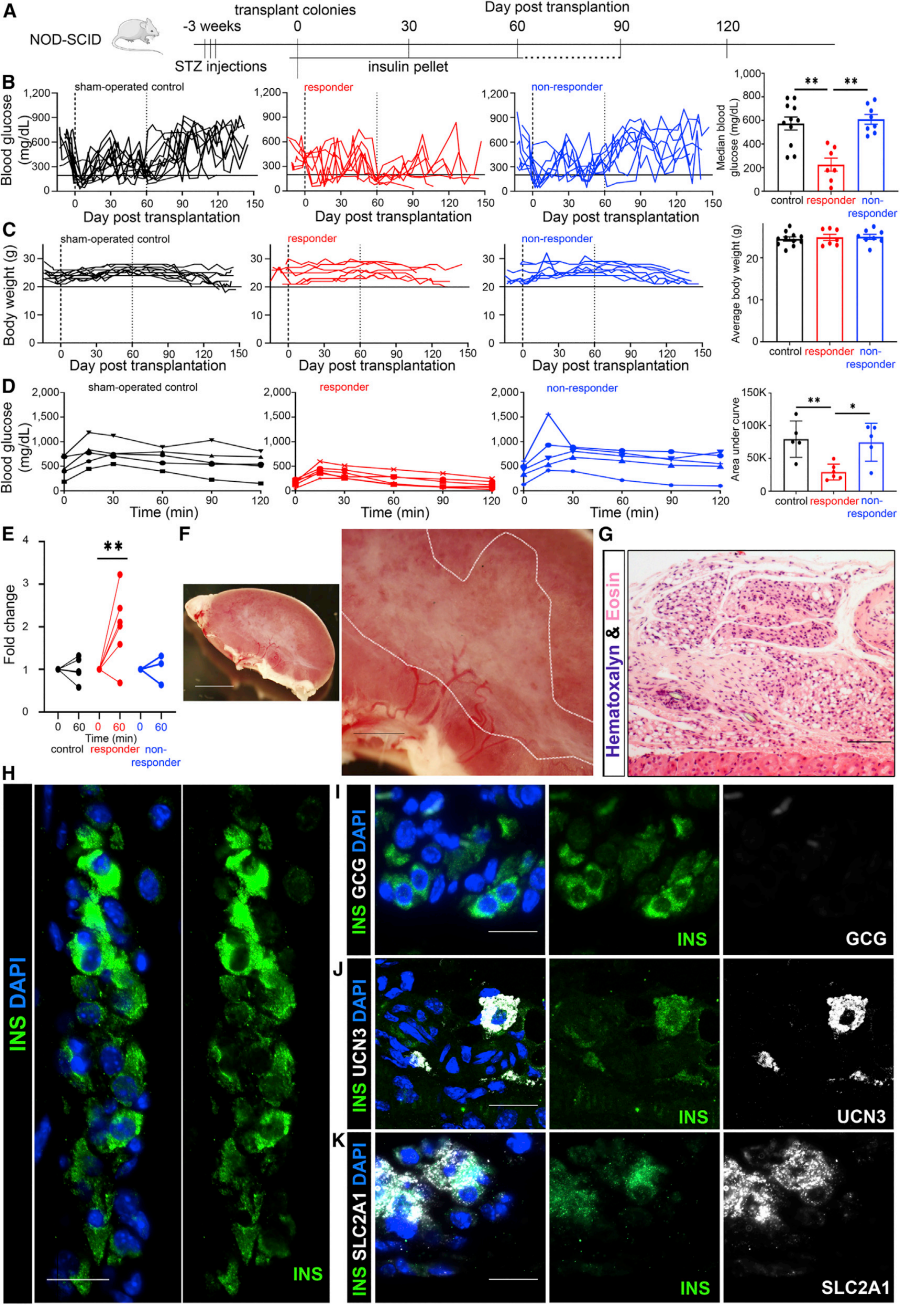
DAPT-treated human colonies give rise to insulin-expressing cells in insulin-dependent diabetic mice (A–C) (A) Experimental diagram. Data were analyzed between day 90 and 120 post-transplantation for (B) blood glucose and (C) body weight, with individual mice separated by sham-operated control (black, n = 11), responder (red, n = 7) and non-responder (blue, n = 8) mice. Data represent median blood glucose or average body weight ± SEM. (D) IP-GTT analysis on control (black, n = 5), responder (red, n = 6), and non-responder (blue, n = 5) mice. AUC was analyzed as mean ± SD. (E) Human C-peptide in serum, expressed as relative C-peptide fold change between time 0 and 60 min post glucose challenge from control (black, n = 4), responder (red, n = 6), and non-responder (blue, n = 3) mice. (F) Bright-field image of a kidney grafted with DAPT-treated human colonies. Grafted cells are outlined (white dashed line). Scale bars, 3 mm (left) or 1 mm (right). (G) H&E staining of a kidney graft, with kidney tissue shown at the bottom of the image. Scale bar, 200 μm. (H–K) IF staining of grafted cells with INS (green) (G and H), GCG (white) (H), UCN3 (white) (J), or SLC2A1 (white) (K). Scale bar, 20 μm. ∗p < 0.05, ∗∗p < 0.01. See also Figures S5 and S6.

We determined whether DAPT-treated human colonies may further differentiate and become functionally mature in insulin-dependent diabetic NOD-SCID mice (Figure 6A). Streptozotocin was injected to mice to destroy beta cells and induce hyperglycemia (fasting blood glucose >200 mg/dL). Subsequently, 3-wo DAPT-treated colonies were pooled and placed under the kidney capsule at 1–2.5 million cells per mouse. An insulin pellet was inserted when fasting blood glucose exceeded 450 mg/dL to minimize the detrimental effects of overt hyperglycemia (Brereton et al., 2016). To test graft function without interference, insulin pellets were not inserted 60 days post-transplantation. Colonies from 4 different donor tissues were independently transplanted into multiple mice (n ≥ 2 mice per donor tissue, 15 mice total).

Between days 90 and 120 post-transplantation, body weight was similar (Figure S5A) while median fasting blood glucose trended lower but did not reach significance in diabetic mice transplanted with colonies compared with sham controls (Figure S5B; individual mouse data in Figure S5C). However, a difference was found when we ranked transplant recipients based on their median fasting blood glucose, separating the top 8 from the bottom 7 mice—the bottom 7 mice (herein “responders”) displayed a median fasting blood glucose at ∼200 mg/dL (Figure 6B). Control diabetic mice did not approach a median fasting blood glucose of 200 mg/dL (Figure 6B), suggesting a lack of beta cells; this was confirmed using H&E staining (Figure S5D). Again, body weights were not different between responders and non-responders (Figure 6C). Importantly, colonies from each donor tissue transplanted into diabetic mice resulted in at least one responder mouse (Figure S5E); this suggests that 1–2.5 million DAPT-treated cells per mouse are the marginal mass, the minimum number of cells necessary to reverse hyperglycemia in some but not all transplanted diabetic mice.

To further analyze graft function, we performed an intra-peritoneal glucose tolerance test (IP-GTT), which revealed a trend of better glucose clearance in transplanted mice (Figure S5F), with a significant decrease in mean area under the curve (AUC) in the responder mice (Figure 6D). Human C-peptide levels in blood 1 h after glucose stimulation significantly increased in the responder mice (Figure 6E). However, stimulated human C-peptide concentration was 2.8 ± 1.9 pmol/L, which was much lower than the 366 ± 154 pmol/L of human C-peptide detected in hyperglycemic NOD-SCID mice transplanted with 1,200 human islets (Table S1) 90–150 days post-transplantation (Figure S5G). This result prompted us to calculate the ratio of blood glucose to human C-peptide in individual mice; a lower ratio indicates a better function of the graft (Takita and Matusmoto, 2012). The colony transplanted mice showed higher ratio than the mice with human islet grafts (Figure S5H), suggesting functional immaturity of our grafted colonies *in vivo* compared with adult islets.

To detect beta-like cells in the transplant mice, we dissected the kidney grafts (Figures 6F and 6G) 3–4 months post-transplantation for IF analysis. INS^+^ cell clusters that did not co-express GCG were found (Figures 6H and 6I), suggesting the presence of mono-hormonal beta-like cells (Herrera, 2000). Some INS^+^ cells also co-expressed beta cell maturation markers UCN3 or SLC2A1 (Figures 6J, 6K, S6A, and S6B). Together, these results show that colonies pre-treated with DAPT can differentiate into beta-like cells after transplantation into diabetic mice.

### scRNA-seq analysis reveals a subset of ductal cells as progenitor-like cells

**Figure 7:**
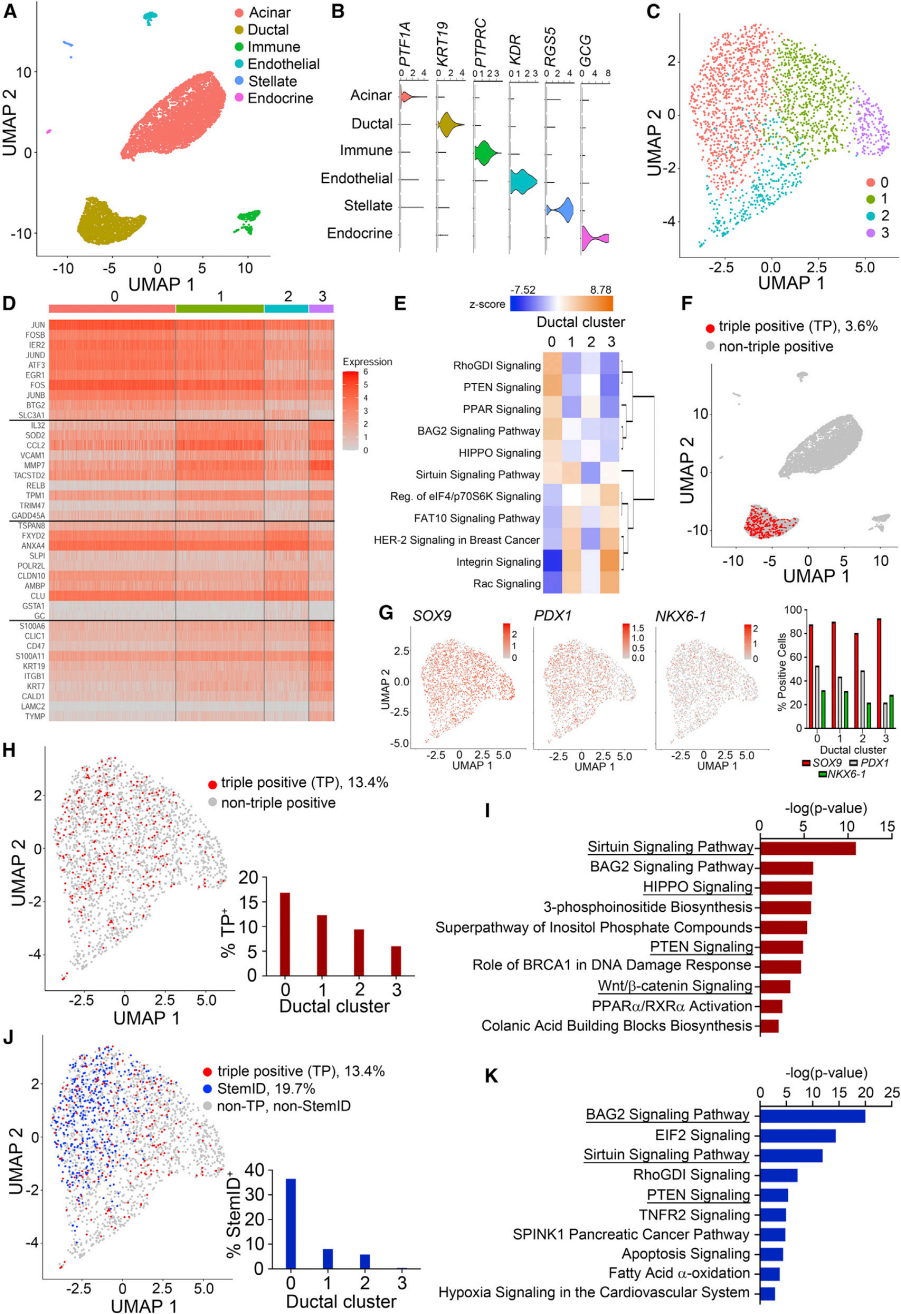
Human ducts are heterogeneous with a subset resembling progenitor-like cells (A) scRNA-seq of dissociated adult human exocrine tissue identifies 6 distinct clusters. (B) Violin plots of representative gene markers. (C) The ductal cluster was re-analyzed by principal-component analysis and segregated into four unique clusters (0–3). (D) Heatmap of cluster-specific genes identified in the four ductal clusters. (E) IPA predicted up and downregulated pathways using DE genes from the four ductal clusters. (F) Cells simultaneously expressing *SOX9*, *PDX1*, and *NKX6.1* are 3.6% of the total cells, and only in the ductal cell population. (G) Uniform Manifold Approximation and Projections (UMAPs) of the ductal cluster showing the expression of the *SOX9*, *PDX1*, or *NKX6.1*, with percent positive cells calculated in each ductal cluster. (H) TP cells are enriched in ductal cluster 0. (I) IPA-predicted upregulated pathways in TP cells. (J) StemID cluster 4 cells (blue) are overlaid with TP cells (red) within the ductal cluster. (K) IPA-predicted upregulated pathways in StemID cluster 4. See also Figure S7 and Data S1.

To gain insight into gene expression patterns of human pancreatic ducts, we performed scRNA-seq analysis using freshly dissociated exocrine tissue. An estimated 14,822 cells were read at 103,333 mean reads per cell. Events that passed quality control (7,812 cells, Figures S7A and S7B) were subsequently analyzed using the Seurat R package. Using Uniform Manifold Approximation and Projection dimensional reduction, we identified clusters of acinar (*PTF1A*: 5,094 cells), ductal (*KRT19*: 2,119 cells), immune (*PTPRC*: 326 cells), endothelial (*KDR*: 185 cells), stellate (*RGS5*: 50 cells), and endocrine cells (*GCG*: 38 cells) (Figures 7A and 7B). Consistent with the sorting results (Figure 3), the ductal cell cluster expressed *PROM1* (CD133) with minimal expression of *ITGA6* (CD49f) (Figure S7C).

To identify potential progenitor cell population within ductal cells, we performed unbiased principal-component analysis of the ductal cluster and found 4 distinct subpopulations (Figure 7C); the top 10 cluster-specific genes are presented in a heatmap (Figure 7D; differentially expressed [DE] genes in Data S1). Cluster 0 was identified with progenitor genes such as *JUN* and *FOS* (Goncalves et al., 2021), whereas cluster 3 showed more mature ductal genes such as *KRT19* and *KRT7*. Bioinformatics analysis using Ingenuity Pathway Analysis (IPA) software revealed the top 6 predicted upregulated and top 5 downregulated canonical pathways for cluster 0, which showed both similar and divergent pathways against other ductal clusters (Figure 7E). These results support the heterogeneity hypothesis on adult human pancreatic ductal cells.

TP cells were exclusively found in the ductal cluster (Figure 7F), consistent with IF analysis in ducts (Figures 2G and 2H). Independent assessment of *SOX9*, *PDX1*, or *NKX6.1* expression in total cells (Figure S7D) and within the four ductal clusters revealed that *PDX1* and *NKX6.1* were the limiting factors (Figure 7G). Overall, TP cells were 3.6% of total cells (Figure 7F) and 13.4% of ductal cells (Figure 7H), with a majority found in cluster 0 (Figure 7H); these percentages were within range of colony-forming efficiency of unsorted and sorted CD133^+^CD49^flow^ ductal (P5) cells (Figure 3E, left). DE genes between the TP cells and other non-TP ductal cells (Data S1) were analyzed by IPA, which revealed upregulated Sirtuin, HIPPO, PTEN, and Wnt-β catenin pathways in TP cells (Figure 7I). These pathways are known to be involved in pancreas development.

The StemID algorithm (Grun et al., 2016) was applied to independently re-cluster all of the ductal cells, and each new cluster according to their stem potential was scored. We selected the cluster with the highest score, StemID cluster 4 (Figures S7E–S7I), and re-mapped those cells to the original clustering analysis. StemID cluster 4 was mapped to 19.7% of the total ductal cells and 36.5% of cluster 0 ductal cells (Figure 7J). DE gene expression of StemID cluster 4 compared with other ductal cells (Data S1) was analyzed in IPA. Predicted upregulated pathways, such as BAG2, Sirtuin, and PTEN signaling, were identified in the StemID cells (Figure 7K); they were also found in the TP cell analysis, indicating similarities between the two populations. Together, these data provide evidence that a subset of ductal cells have progenitor properties.

## Discussion

In this study, we show evidence for the existence of self-renewing progenitor-like cells from the adult human pancreatic ducts, which we call PCFUs. By using single-cell micro-manipulation, we provide a rigorous demonstration of multi-lineage differentiation potential of PCFUs *in vitro*. In addition, we show for the first time that colonies and adult human ducts *in vivo* contain cells capable of expressing embryonic MPC markers (SOX9^+^/PDX1^+^/NKX6.1^+^), which we call TP cells.

Tremendous progress has been made in 3D organoid technology. Many current organoid culture techniques are modeled after a study that established epithelial organoid culture using Matrigel (Sato et al., 2011), where high concentrations (>90% v/v) of Matrigel are used to embed and immobilize cells. However, it is difficult to micro-manipulate individual organoids; one reason is the elevated viscosity and rigidity caused by the high Matrigel concentrations. Our 3D colony assay differs in that viscous methylcellulose is added (Perko et al., 2011), allowing the dilution of Matrigel to a much lower concentration (5% v/v in this study) and aiding micro-manipulation.

Using organoid culture systems based on the intestinal organoid platform from Sato et al., several studies show that pancreatic ductal cells can expand *in vitro*, but those ductal cells possess only two lineage potential (duct and endocrine) or with limited self-renewal. Loomans et al. (2018) found that the net expansion of total ductal cells was approximately 20-fold over 20 weeks. Lee et al. (2013) observed an 8-fold increase of total cells over 6–9 weeks, and Georgakopoulos et al. (2020) showed an impressive expansion of organoids for over 15 weeks. In contrast, rather than mechanical digestion into cell clumps as in the other studies, we expanded our colonies by enzymatic digestion to single cells during passaging, observing up to 300-fold expansion of PCFUs over 9 weeks (Figure 4). In addition, PCFUs comprise ∼8% of the total cells in the 3° culture (Figure S3G); therefore, total expansion of our ductal cells is calculated to be about 3,750-fold. We also report that colonies after expansion maintain the same gene expression patterns as colonies from the first culture, showing preservation of tri-lineage potency (Figure 4E). Thus, PCFUs may represent the true self-renewing progenitor cells from the adult human pancreas.

With respect to lineage potential, Loomans et al. (2018) showed that progenitor-like cells marked by ALDH^high^ staining possess duct and endocrine lineage potential. In contrast, our PCFUs possess duct, endocrine, and acinar lineage potential. Lee et al. (2013) reported that their ductal cells cannot be transdifferentiated into INS^+^ cells in organoid culture unless forced to express NGN3, MAFA, and PDX1. Interestingly, Qadir et al. (2018, 2020) demonstrated that sorted adult human P2RY1^+^/ALK3^bright^ cells, which are found in the main ducts, can give rise to the three major pancreatic lineages using a 2D attachment culture system. Our data agree with their findings that some ductal cells possess tri-lineage potential. However, Qadir et al. did not report the self-renewal capacity of the P2RY1^+^/ALK3^bright^ cells, which is an important aspect of progenitor cells.

Cellular compartments in the adult pancreas had been largely considered homogeneous, but increasing evidence suggests that endocrine (Baron et al., 2016; Butler et al., 2010), acinar (Kusmartseva et al., 2020), and ductal cells (Baron et al., 2016; Grun et al., 2016; Qadir et al., 2020) are heterogeneous. In this study, we not only confirm ductal cell heterogeneity among adult human exocrine tissue (Figures 2G and 7), but we also add functional heterogeneity in colony formation among sorted human ductal cells (Figure 3E). This ductal cell heterogeneity may explain the difficulties of Cre-lox lineage tracing using a pan-duct marker, such as *Sox9* (Kopp et al., 2011) or *Hnf1b* (Solar et al., 2009), to detect significant activities of adult murine pancreatic progenitor cells due to the relatively minor population of progenitor cells. In addition, subtle differences in the expression levels of progenitor cell markers may dictate functionality (Rezanejad et al., 2018). Thus, to address ductal cell heterogeneity further, future experiments are needed to identify unique cell surface markers in combination with CD133, but not CD49f, that enrich or purify the ductal progenitor cells.

A potential clinically relevant finding of our study is that DAPT-treated colonies grafted into diabetic mice give rise to beta-like cells *in vivo*. Although, our grafts did not result in high levels of human C-peptide, our transplant mice did show an observable drop in blood glucose levels (i.e., in responder mice) between 3 and 4 months post-transplantation (Figure 6B). These results raise the possibility for proinsulin, rather than C-peptide or insulin, as the predominant form of the insulin gene product that is secreted from our colony grafts—a possibility that requires future investigation. Proinsulin has been reported to exert biological effects in development and various adult cell types (Malaguarnera et al., 2012) and therefore may provide clinically beneficial effects to the hyperglycemic mice. Loomans et al. (2018) transplanted ductal organoids under the kidney capsule of mice (up to 4.5 × 10^5^ cells per mouse) and detected INS^+^KRT19^−^ cells. However, their mice were followed for only 1 month post-transplantation; it remains unknown whether their INS^+^ cells improve glucose regulation over a longer period. Pluripotent stem cell (PSC)-derived insulin-expressing cells have been shown to regulate blood glucose levels in insulin-dependent diabetic mice after transplantation (Migliorini et al., 2021). However, there is the concern of teratoma formation from undifferentiated PSCs (Cunningham et al., 2012). In contrast to PSCs, adult stem cells do not give rise to teratomas. Thus, should PSC-derived products raise safety concerns in future clinical trials, adult PCFUs can be a suitable alternative source of insulin-expressing cells.

In summary, we have shown in functional *in vitro* assays that some adult human ductal cells, resembling progenitor cells, are capable of tri-lineage differentiation and self-renewal in a unique 3D methylcellulose-containing culture system. Also, we identified a subset of human pancreatic ductal cells capable of expressing TP progenitor markers through IF and *in silico* analysis. Given the severe shortage of donor organs, our results suggest a potential utility of human cadaveric ductal tissues for therapy in insulin-dependent diabetic patients.

## Experimental procedures

### Resource availability

#### Corresponding author

The data that support the findings of this study are available from the corresponding author, Janine C. Quijano (jquijano@coh.org), upon reasonable request.

#### Materials availability

This study did not generate new unique reagents.

#### Single-cell suspension

Donated pancreata were procured and shipped to City of Hope for isolation of islets (Qi et al., 2015). All tissues used in this study had consent for research from close relatives of the donors. After islet removal, de-identified human pancreata were obtained from the Southern California Islet Cell Resource (SC-ICR) Center at the City of Hope. The exocrine tissue was dissociated to yield a single-cell suspension before cryopreservation, culture, or other procedures.

#### Mice

Mice used in this study were maintained according to protocols approved by the City of Hope Institutional Animal Care and Use Committee.

Additional detailed experimental methods are provided in the supplemental information.

## Author contributions

Conceptualization, J.C.Q., L.W., and H.T.K.; methodology, J.C.Q., L.W., and H.T.K.; software, M.-H.C.; formal analysis, J.C.Q. and L.W.; investigation, J.C.Q., L.W., J.A.O., J.M.L., A.L., J.R., J.M.M., K.L., H.N.Z., J.R.T., K.J., and C.M.-D.; resources, I.H.A. and F.K.; writing – original draft, J.C.Q., L.W., and H.T.K.; writing – review & editing, J.C.Q., L.W., J.A.O., H.N.Z., J.R.T., I.H.A., D.C.T., F.K., A.D.R., and H.T.K.; supervision, J.C.Q., L.W., and H.T.K.; funding acquisition, J.C.Q., L.W., and H.T.K.

## Data Availability

The data that support the findings of this study are available from the corresponding author upon reasonable request. Single-cell RNA sequencing (scRNA-seq) data are available from Gene Expression Omnibus (GEO) database: GSE153834.

## Supplemental information

Document S1. Figures S1–S7, Tables S1–S5, and supplemental experimental procedures

Data S1. Differential expressed (DE) genes between various ductal clusters, related to Figure 7

Document S2. Article plus supplemental information
