# Supplementary material for: Methylcellulose colony assay and single-cell micro-manipulation reveal progenitor-like cells in adult human pancreatic ducts
Source: Stem Cell Reports. 2023 Mar 2;18(3):618–35. doi: 10.1016/j.stemcr.2023.02.001 (PMC10031308; doi:10.1016/j.stemcr.2023.02.001)
Supplement: Document S1. Figures S1–S7, Tables S1–S5, and supplemental experimental procedures [file mmc1.pdf]

Stem Cell Reports, Volume *18*

Supplemental Information

Methylcellulose colony assay and single-cell micro-manipulation reveal progenitor-like cells in adult human pancreatic ducts

Janine C. Quijano, Lena Wedeken, Jose A. Ortiz, Heather N. Zook, Jeanne M. LeBon, Angela Luo, Jeffrey Rawson, Jacob R. Tremblay, Jacob M. Mares, Kassandra Lopez, Min-Hsuan Chen, Kevin Jou, Carlos Mendez-Dorantes, Ismail H. Al-Abdullah, Debbie C. Thurmond, Fouad Kandeel, Arthur D. Riggs, and Hsun Teresa Ku

SUPPLEMENTARY MATERIALS

This document contains the following information.

1. **Figure S1 to S7.**
2. **Table S1 to S5.**
3. **Supplemental experimental procedures.**
4. **Supplemental references.**

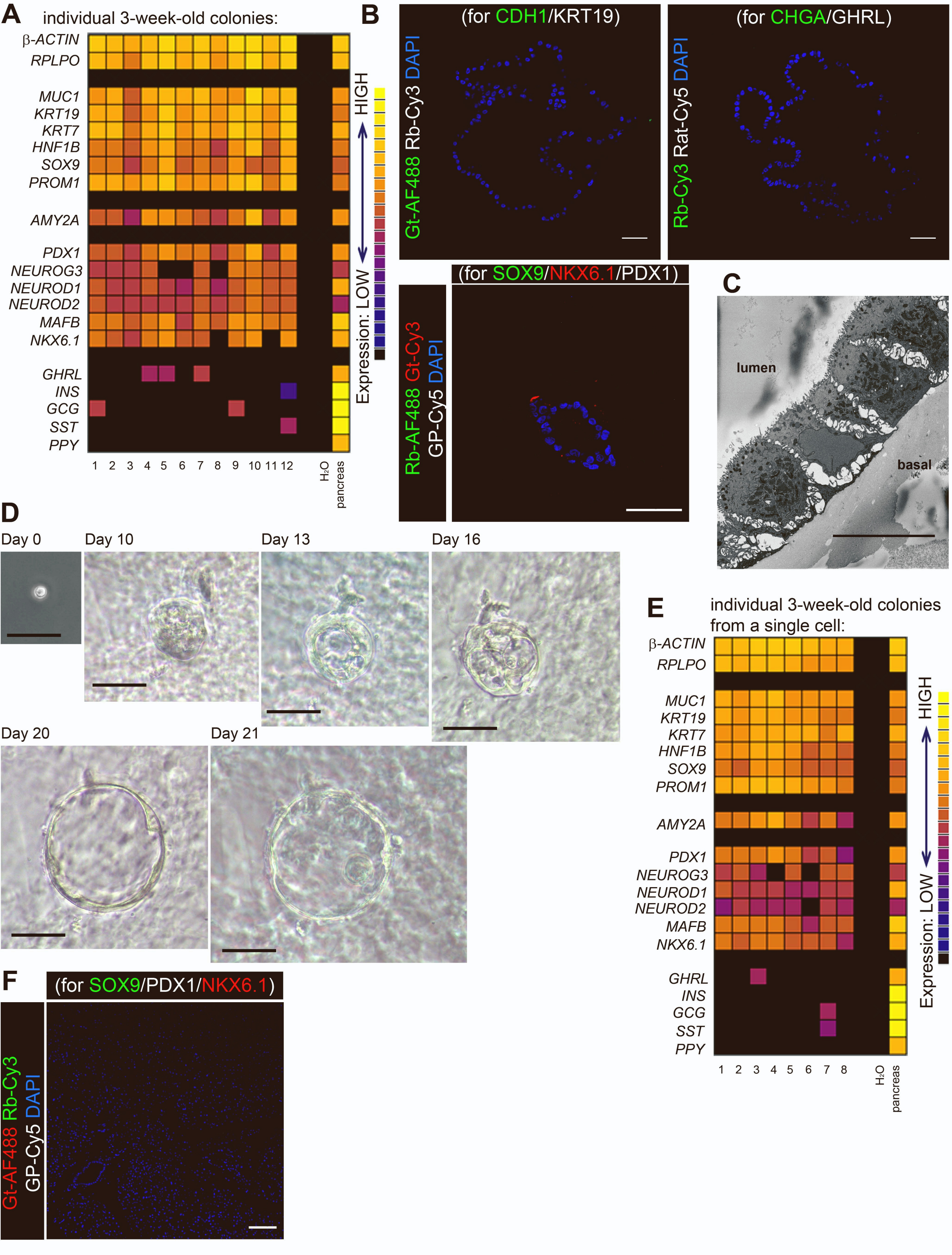

A
individual 3-week-old colonies:
β-ACTIN
RPLPO
MUC1
KRT19
KRT7
HNF1B
SOX9
PROM1
AMY2A
PDX1
NEUROG3
NEUROD1
NEUROD2
MAFB
NKX6.1
GHRL
INS
GCG
SST
PPY
1 2 3 4 5 6 7 8 9 10 11 12
H2O
pancreas
Expression: LOW ↔ HIGH
B
(for CDH1/KRT19)
Gt-AF488 Rb-Cy3 DAPI
(for CHGA/GHRL)
Rb-Cy3 Rat-Cy5 DAPI
(for SOX9/NKX6.1/PDX1)
Rb-AF488 Gt-Cy3 GP-Cy5 DAPI
C
lumen
basal
D
Day 0
Day 10
Day 13
Day 16
Day 20
Day 21
E
individual 3-week-old colonies from a single cell:
β-ACTIN
RPLPO
MUC1
KRT19
KRT7
HNF1B
SOX9
PROM1
AMY2A
PDX1
NEUROG3
NEUROD1
NEUROD2
MAFB
NKX6.1
GHRL
INS
GCG
SST
PPY
1 2 3 4 5 6 7 8
H2O
pancreas
Expression: LOW ↔ HIGH
F
(for SOX9/PDX1/NKX6.1)
Gt-AF488 Rb-Cy3 GP-Cy5 DAPI

Fig. S1: Characterization of 3-week-old colonies grown in our standard colony assay (associated with Fig. 1 and 2).

(A) Heatmap example of micro-fluidic qRT-PCR from 58 individually micro-manipulated colonies from 7 donors, associated with Fig. 1H. (B) Negative control images for antibodies used in Fig. 1I. Scale bars = 50 μm. (C) A representative image from 3-dimensional scanning electron microscopy (3D-SEM) of a 3-week-old colony derived from unsorted cells grown in the standard colony assay. The lumen of the colony is to the left of cells. Cells in colonies are polarized and ductal-like. Scale bar = 100 μm. (D) Tracking of a single cell to colony formation. The single cell was imaged and then micro-manipulated into a well of a 96-well plate. The resulting colony was identified on Day 10 and tracked to Day 21. Another colony is shown in Fig. 2B. Scale bar = 50 μm for all images. (E) Representative heatmap of micro-fluidic qRT-PCR from 22 (1 cell/well) individually micro-manipulated colonies in 3 independent experiments from 2 donors, associated with Fig. 2E. (F) Negative control image for antibodies used in Fig. 2G. Scale bar = 100 μm.

A

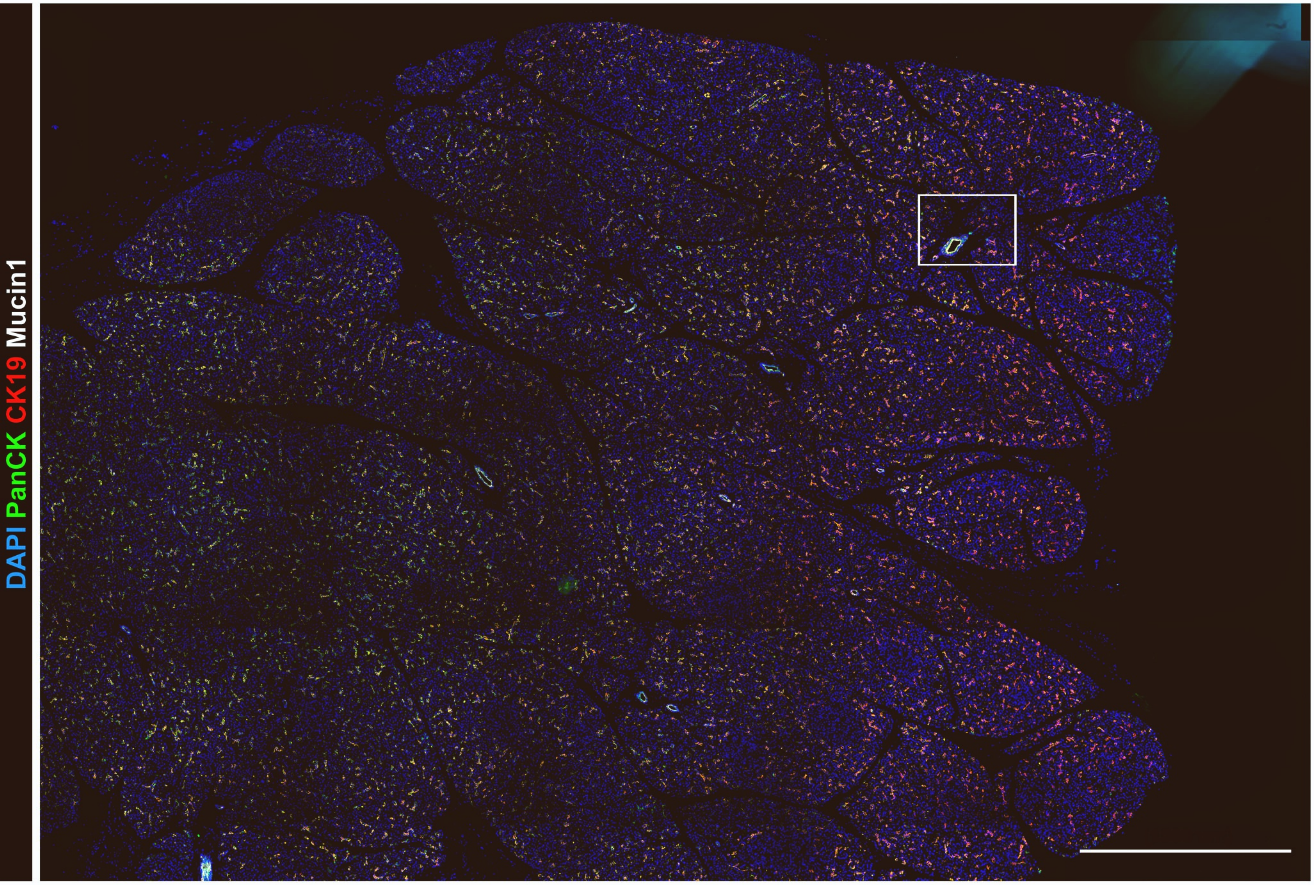

B

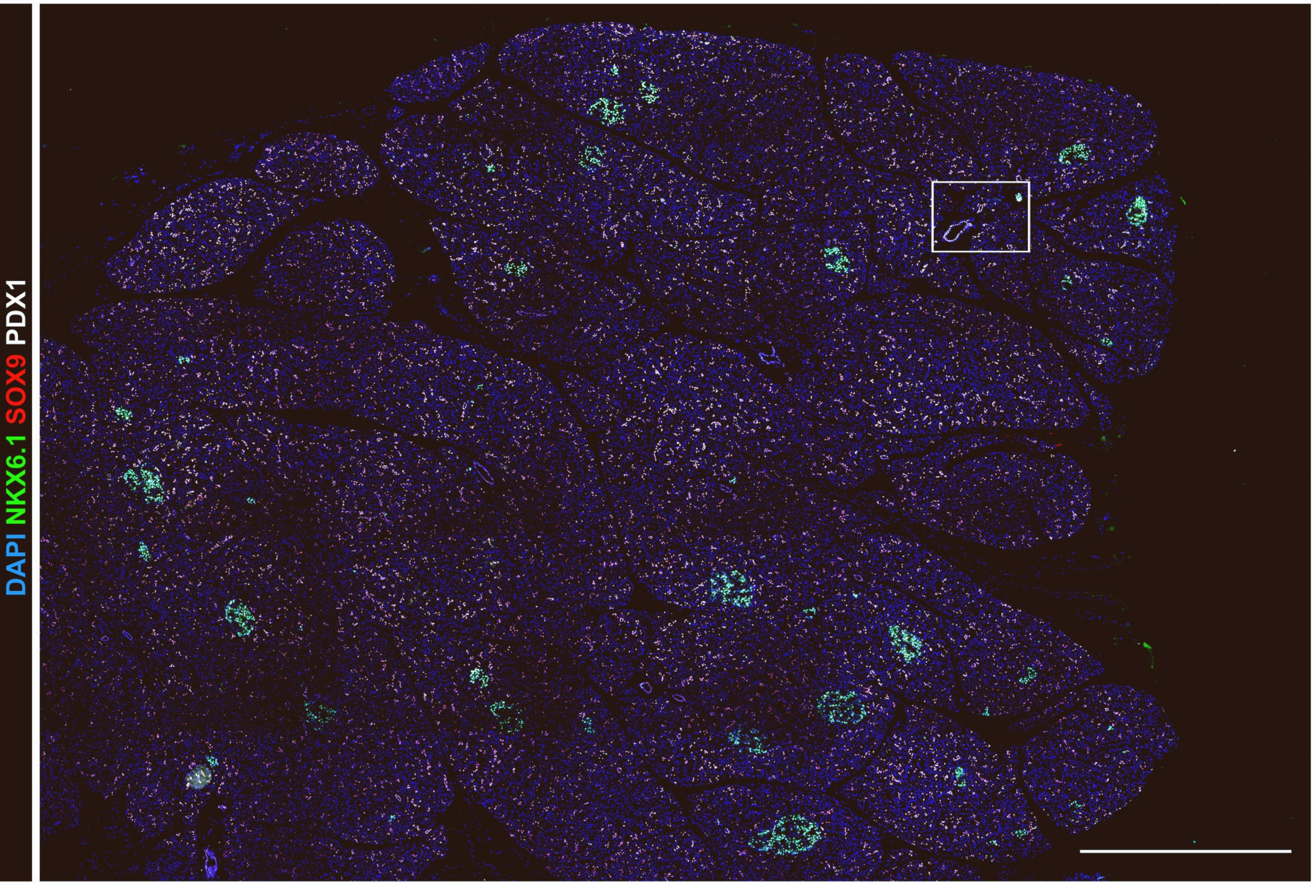

Fig. S2: Stitched images of human pancreas stained with ductal markers or in a sequential slide with SOX9/PDX1/NKX6-1 (associated with Fig. 2F-H).

Examples of sequential slides from a human pancreas that was triple stained for (A) ductal markers Pan-CK (green), CK19 (red) and Mucin1 (white), or (B) NKX6.1 (green), SOX9 (red) and PDX1 (white). Imaging was performed on a Zeiss Observer and the individual images stitched together and presented from 4 different donor tissues. White box indicates areas imaged with a confocal microscope shown in Fig. 2F and 2G. Scale bar = 1 mm. Quantification of SOX9+/PDX1+/NKX6-1 triple positive cells (shown in Fig. 2H) was done using QuPath software.

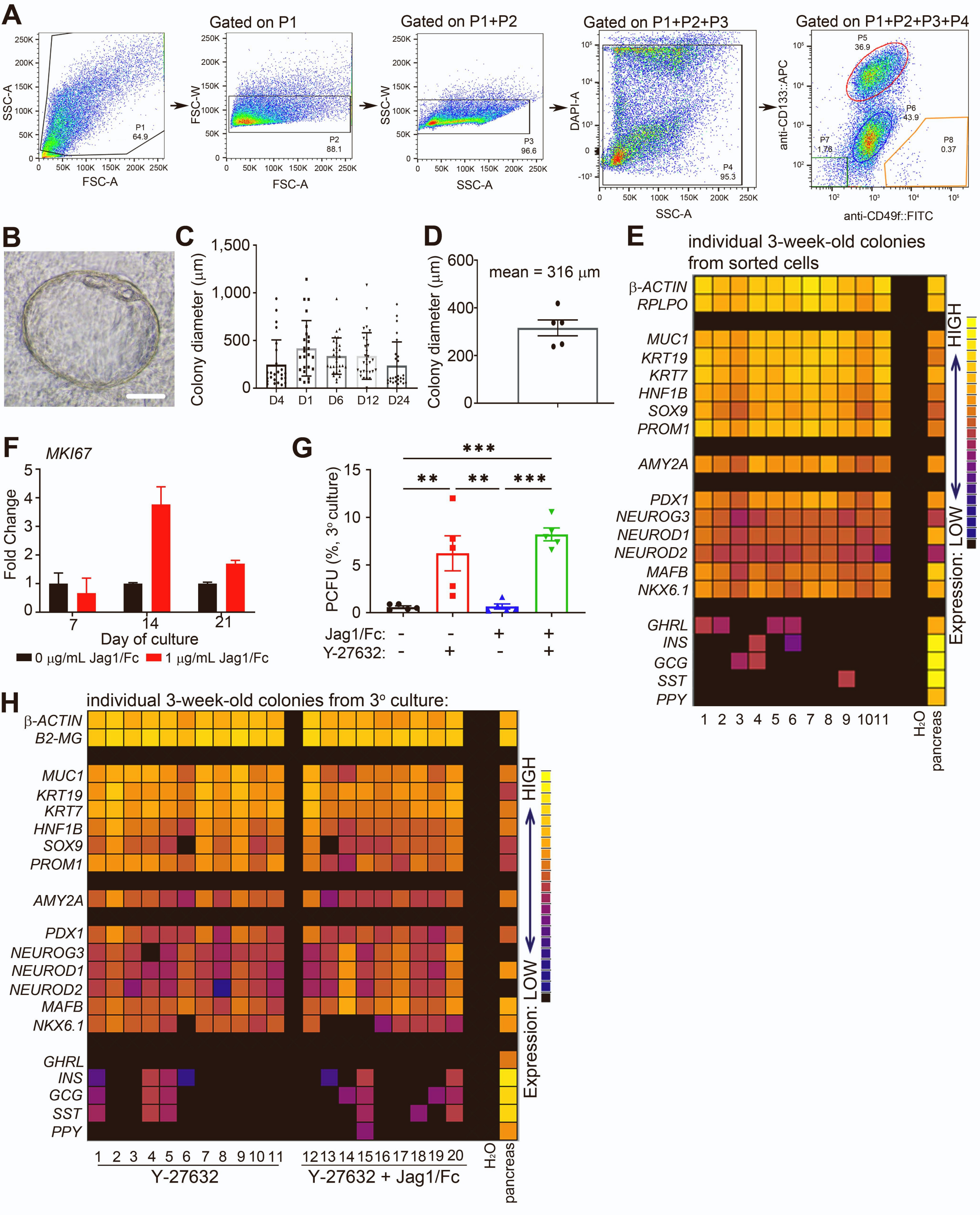
A
Gated on P1
Gated on P1+P2
Gated on P1+P2+P3
Gated on P1+P2+P3+P4
SSC-A
FSC-A
P1 64.9
FSC-W
P2 88.1
SSC-W
SSC-A
P3 96.6
DAPI-A
P4 95.3
anti-CD133::APC
anti-CD49f::FITC
P5 36.9
P6 43.9
P7 1.78
P8 0.37
B
C
Colony diameter (μm)
D4
D1
D6
D12
D24
D
mean = 316 μm
Colony diameter (μm)
E
individual 3-week-old colonies from sorted cells
β-ACTIN
RPLPO
MUC1
KRT19
KRT7
HNF1B
SOX9
PROM1
AMY2A
PDX1
NEUROG3
NEUROD1
NEUROD2
MAFB
NKX6.1
GHRL
INS
GCG
SST
PPY
1 2 3 4 5 6 7 8 9 10 11
H₂O
pancreas
HIGH
Expression: LOW
F
MKI67
Fold Change
Day of culture
7
14
21
0 μg/mL Jag1/Fc
1 μg/mL Jag1/Fc
G
PCFU (%, 3° culture)
***
**
**
***
Jag1/Fc: - - + +
Y-27632: - + - +
H
individual 3-week-old colonies from 3° culture:
β-ACTIN
B2-MG
MUC1
KRT19
KRT7
HNF1B
SOX9
PROM1
AMY2A
PDX1
NEUROG3
NEUROD1
NEUROD2
MAFB
NKX6.1
GHRL
INS
GCG
SST
PPY
1 2 3 4 5 6 7 8 9 10 11
12 13 14 15 16 17 18 19 20
Y-27632
Y-27632 + Jag1/Fc
H₂O
pancreas
HIGH
Expression: LOW

Fig. S3: Characterization of 3-week-old colonies derived from FACS sorted cells and serially replated colonies in 3º culture (associated with Fig. 3 and 4).

(A) Representative flow cytometry gates for sorting $CD133^{+}CD49f^{low}$ cells. To exclude debris, cells were gated on forward scatter (FSC-A) and side scatter (SSC-A) (population [P] 1). To exclude cell doublets, cells were gated by FSC-W (P2) followed by SSC-W (P3). To exclude dead cells, DAPI-negative live cells were gated (P4). The DAPI positive gate for dead cells was set based on pancreatic exocrine cell samples treated with 70% ethanol (not shown). (B) Representative bright-field image of a 3-week-old colony grown from plated $CD133^{+}CD49f^{low}$ cells. Scale bar = 50 μm. (C) The diameters of individual colonies grown from $CD133^{+}CD49f^{low}$ cells were measured from N=5 different donors and did not show donor-to-donor variation. The average and standard deviation of colony diameters are as follows: Donor 4 (n=25), 248 ± 260 μm; Donor 1 (n=25), 419 ± 291 μm; Donor 6 (n=30), 338 ± 191 μm; Donor 12 (n=29), 338 ± 244 μm; and Donor 24 (n=26), 238 ± 248 μm. (D) The mean diameter of 3-week-old colonies grown from sorted $CD133^{+}CD49f^{low}$ cells was 316 ± 34 μm (mean ± SEM) from N=5 donors analyzed in C. (E) Representative heatmap showing the gene expression profiles of individually micro-manipulated colonies grown from $CD133^{+}CD49f^{low}$ cells were analyzed using microfluidic qRT-PCR analysis. Data are representative of a total of n=38 colonies from N=5 donors tested. (F) Cells stimulated with Jag1/Fc (1 ug/ml, red) or without (0 ug/ml, black) were collected at different timepoints and analyzed for *MKI67* RNA expression. Cells from 1 donor were plated in 4 wells per group. The resulting colonies at designated days were pooled and analyzed by conventional qRT-PCR in technical triplicates. (G) The percent of PCFU among cells plated in 3° culture was higher in the presence of Y-27632 alone or a combination of Y-27632 plus Jag1/Fc, n=5 experiments from 4 donors. (H) A representative heatmap from microfluidic qRT-PCR analysis of individually micro-manipulated colonies from 3° culture treated with Y-27632 alone (n=28 colonies) or a combination of Y-27632 plus Jag1/Fc (n=28 colonies). Data are a representative heatmap from N= 3 donors tested. **p<0.01, ***p<0.001.

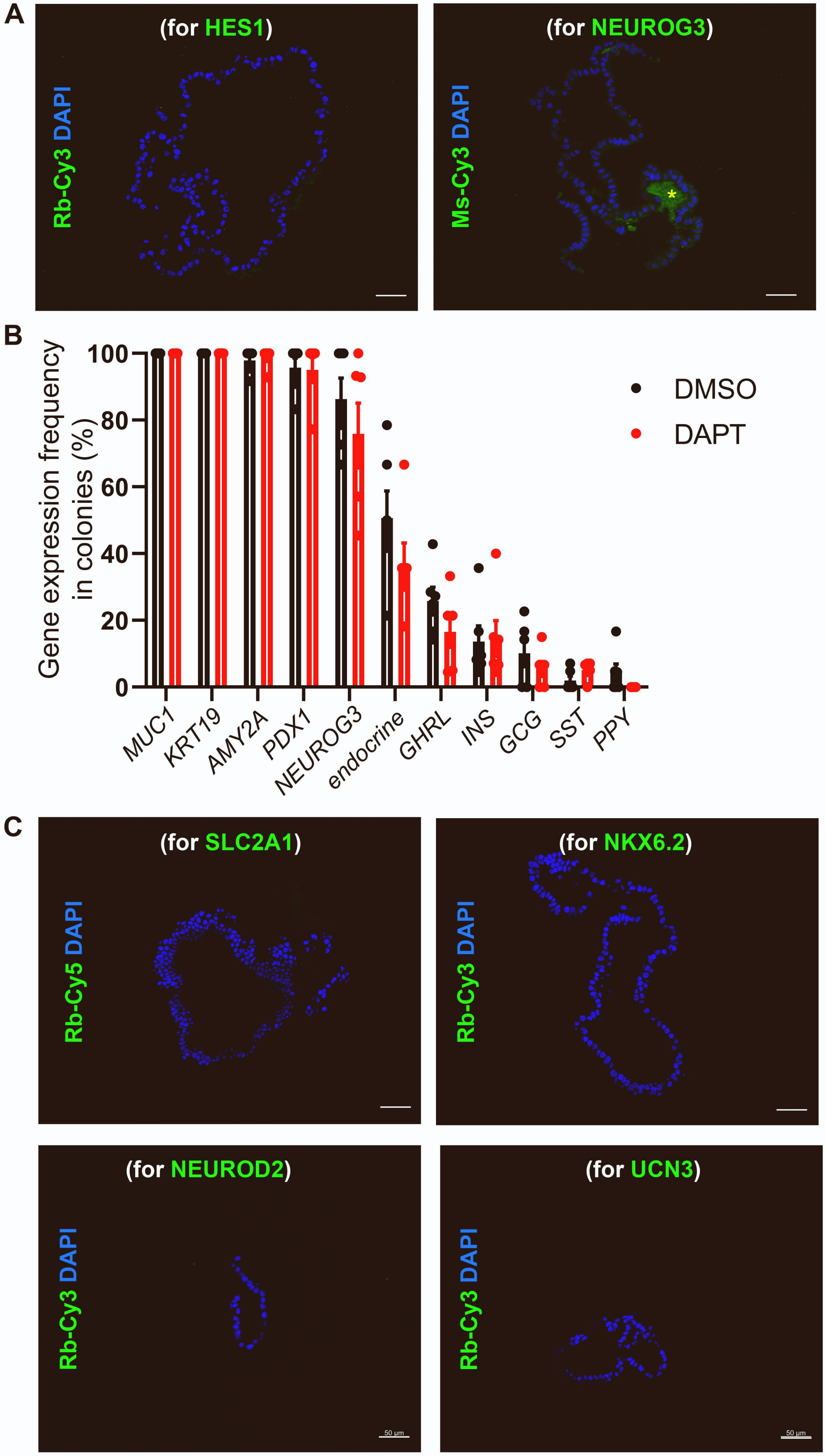
A
(for HES1)
Rb-Cy3 DAPI
(for NEUROG3)
Ms-Cy3 DAPI
*
B
Gene expression frequency in colonies (%)
100
80
60
40
20
0
DMSO
DAPT
MUC1
KRT19
AMY2A
PDX1
NEUROG3
endocrine
GHRL
INS
GCG
SST
PPY
C
(for SLC2A1)
Rb-Cy5 DAPI
(for NKX6.2)
Rb-Cy3 DAPI
(for NEUROD2)
Rb-Cy3 DAPI
(for UCN3)
Rb-Cy3 DAPI
50 μm
50 μm

Fig. S4: Characterization of 3-week-old colonies treated with DAPT (a Notch signaling inhibitor) (associated with Fig. 5).

(A) Sequential slides from 3-week-old colonies treated with DAPT shown in Fig. 5C were used for negative control staining. Secondary antibodies for the respective fluorescence were used, as indicated. Yellow star (*) denotes an area of non-specific staining in image on right. Scale bar = 50 μm. (B) Gene expression frequencies among 3-week-old colonies treated with vehicle (DMSO, black) or a Notch signaling inhibitor (DAPT, red). DMSO: n=95 colonies, DAPT: n=100 colonies from N=6 donors. Data represent mean ± standard deviation. Two-way ANOVA, with Sidak's multiple comparison, was used to determine significance. (C) Sequential slides from 3-week-old colonies treated with DAPT shown in Fig. 5H were used for negative control staining. Secondary antibodies for the respective fluorescence were used, as indicated. Scale bar = 50 μm.

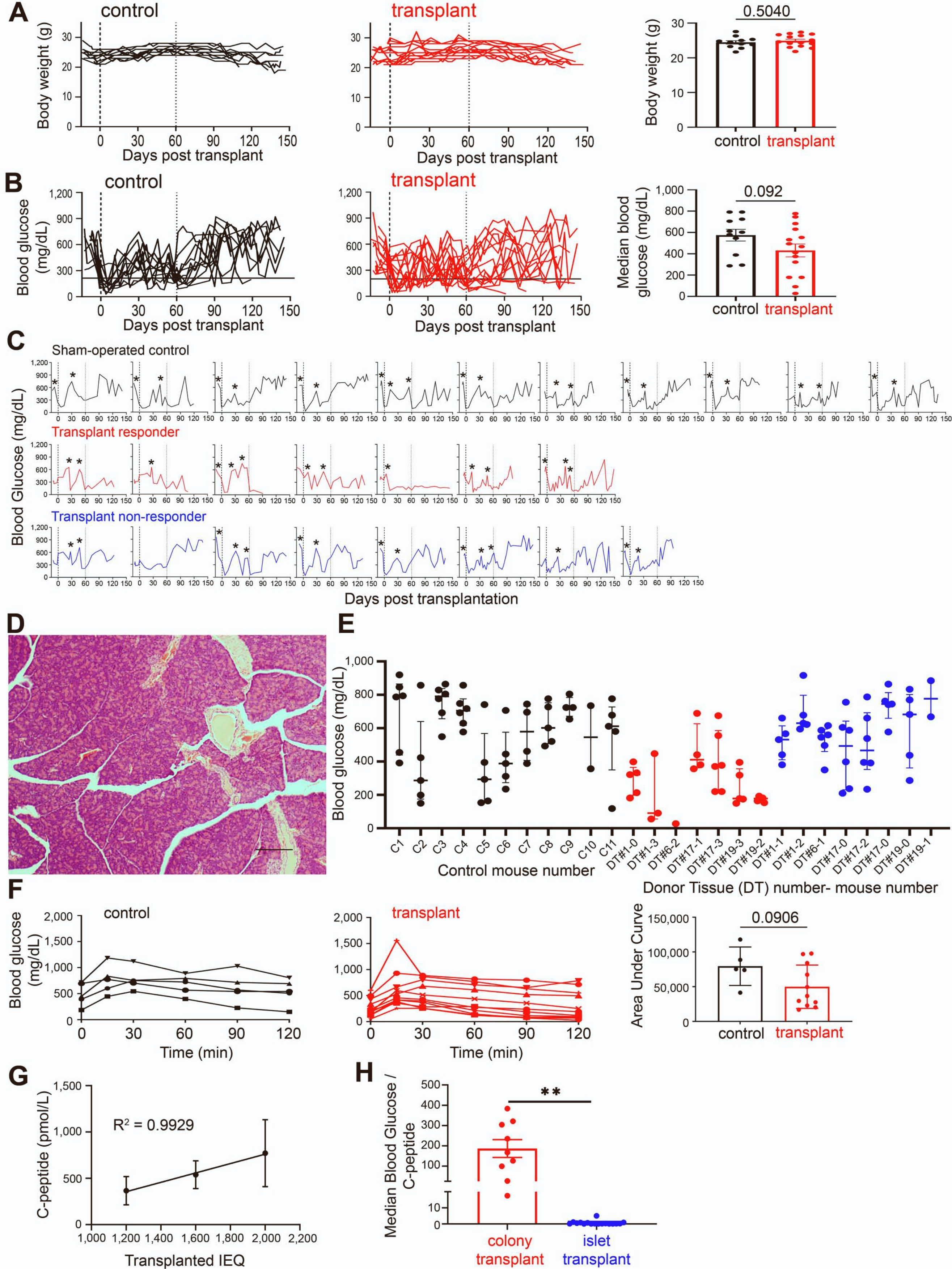
A
control
transplant
Body weight (g)
Days post transplant
0.5040
control
transplant
B
Blood glucose (mg/dL)
Median blood glucose (mg/dL)
0.092
C
Sham-operated control
Transplant responder
Transplant non-responder
Blood Glucose (mg/dL)
Days post transplantation
D
E
Blood glucose (mg/dL)
C1
C2
C3
C4
C5
C6
C7
C8
C9
C10
C11
DT#1-0
DT#1-3
DT#6-2
DT#17-1
DT#17-3
DT#19-3
DT#19-2
DT#1-1
DT#1-2
DT#6-1
DT#17-0
DT#17-2
DT#17-0
DT#19-0
DT#19-1
Control mouse number
Donor Tissue (DT) number- mouse number
F
control
transplant
Blood glucose (mg/dL)
Time (min)
Area Under Curve
0.0906
G
C-peptide (pmol/L)
R² = 0.9929
Transplanted IEQ
H
Median Blood Glucose / C-peptide
**
colony transplant
islet transplant

Fig. S5: Additional data associated with transplantation studies (associated with Fig. 6).
(A) Body weight measurements over time from individual sham-operated control (black) and transplant (red) mice. No difference was found. Data represent mean ± SEM. (B) Blood glucose measurements over time grouped by sham-operated control (black) or transplant (red) mice. Median blood glucose levels (90+ days post-transplantation) of transplant mice trended lower than sham-operated control mice. Data represent median ± SEM. (C) Blood glucose measurements over time from individual mice from sham-operated controls (black), transplant responders (red) and non-responders (blue). Asterisks (*) indicates the day that an insulin pellet was inserted into the mouse. (D) H&E staining of STZ-treated NOD-SCID pancreas 4 months post-transplantation; a representative mouse that received colonies under kidney capsule is shown. Scale bar = 200 μm. (E) Blood glucose measurements from mice between days 90 and 120 post-transplantation. Data represent median ± standard deviation of fasting blood glucose measured at least once per week. (F) An intraperitoneal glucose tolerance test (IP-GTT) was performed 3 months post-transplantation. Fasting blood glucose levels (mg/dL) were graphed, grouping together sham-operated mice (black) and transplant (red) mice. The area under the curve (AUC) of transplant mice trends lower than control mice. Data represent mean ± standard deviation from 5 control and 11 transplant mice. (G) Human islets were transplanted into the kidney capsule of STZ-treated NOD-SCID mice. Serum C-peptide levels were analyzed between 90-150 days post-transplantation. Abbreviation: islet equivalent, IEQ. (H) Ratio of the median blood glucose and measured c-peptide concentration in individual mice indicate that all mice transplanted with DAPT-treated colonies are significantly different than mice transplanted with human islets. **$p<0.01$.

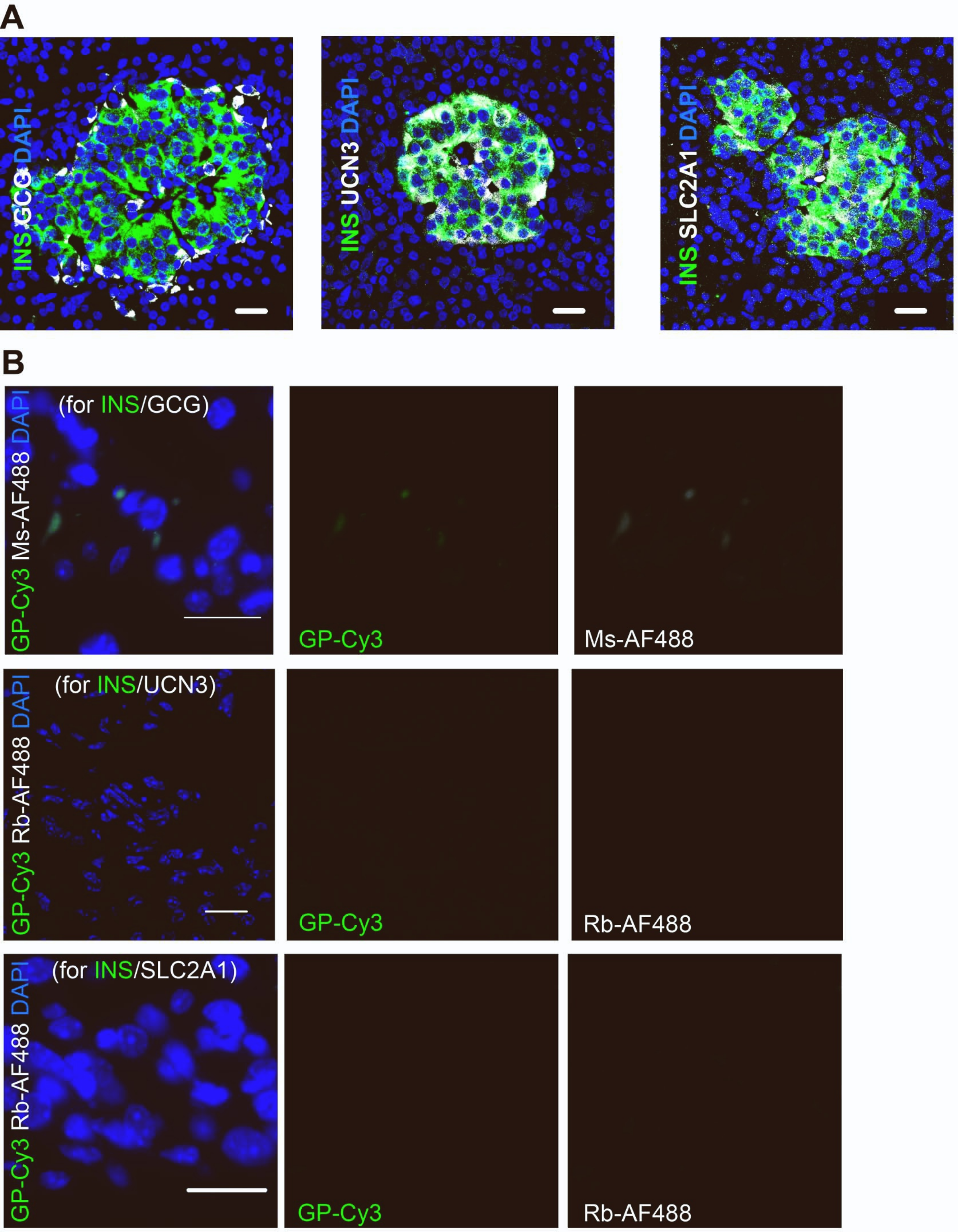

A
INS GCG DAPI
INS UCN3 DAPI
INS SLC2A1 DAPI
B
GP-Cy3 Ms-AF488 DAPI
(for INS/GCG)
GP-Cy3
Ms-AF488
GP-Cy3 Rb-AF488 DAPI
(for INS/UCN3)
GP-Cy3
Rb-AF488
GP-Cy3 Rb-AF488 DAPI
(for INS/SLC2A1)
GP-Cy3
Rb-AF488

Fig. S6: Validation of immunofluorescence staining of the grafts with positive and negative controls (associated with Fig. 6).

(A) Positive control staining. Left: a human islet stained with INS (green) and GCG (white). Middle: a human islet stained with INS (green) and UCN3 (white). Right: a human islet stained with INS (green) and SLC2A1 (white). All nuclei are stained with DAPI (blue). The same antibodies and conditions were used for staining kidney grafts in Fig. 6H-K. Scale bars = 20 μm. (B) Sequential slides from grafts shown in Fig. 6H-K were used for negative control staining (secondary antibodies only). Top row: INS (anti-guinea pig) and GCG (anti-mouse) secondary antibodies. Middle row: INS (anti-guinea pig) and UCN3 (anti-rabbit) secondary antibodies. Bottom row: INS (anti-guinea pig) and SLC2A1 (anti-rabbit) secondary antibodies. Scale bars = 20 μm.

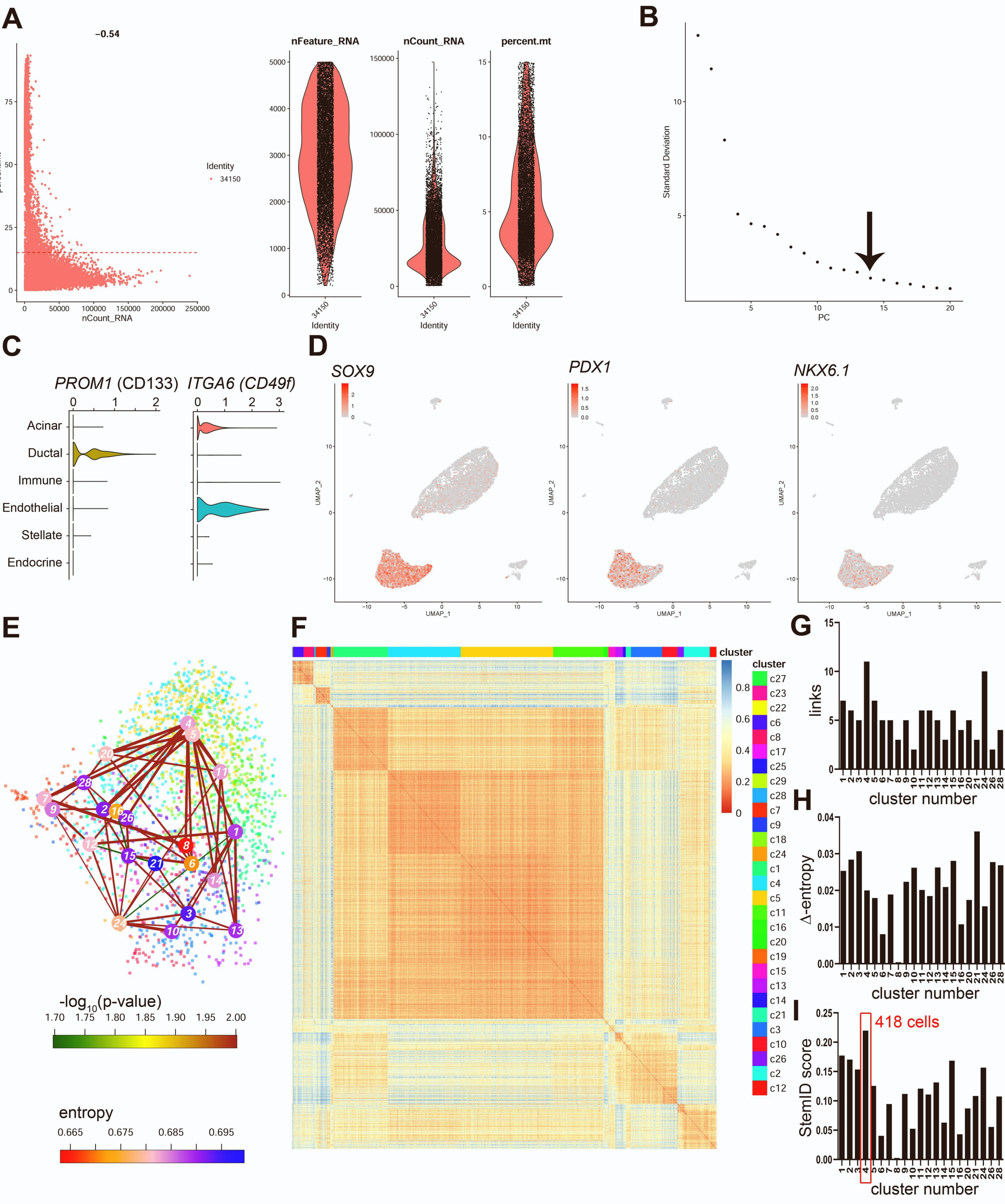

A
−0.54
nCount_RNA
Identity
34150
nFeature_RNA
nCount_RNA
percent.mt
B
Standard Deviation
PC
C
PROM1 (CD133)
ITGA6 (CD49f)
Acinar
Ductal
Immune
Endothelial
Stellate
Endocrine
D
SOX9
PDX1
NKX6.1
UMAP_1
UMAP_2
E
-log10(p-value)
1.70 1.75 1.80 1.85 1.90 1.95 2.00
entropy
0.665 0.675 0.685 0.695
F
cluster
c27 c23 c22 c6 c8 c17 c25 c29 c28 c7 c9 c18 c24 c1 c4 c5 c11 c16 c20 c19 c15 c13 c14 c21 c3 c10 c26 c2 c12
G
links
cluster number
H
Δ-entropy
cluster number
I
StemID score
418 cells
cluster number

Figure S7. Single-cell RNA-sequencing (scRNAseq) analysis of uncultured and dissociated exocrine tissue from adult human pancreas (associated with Fig. 7).

(A) Quality control analysis of scRNAseq data. Events that exceeded higher than 15% mitochondria genes, less than 200 RNA counts and more than 5,000 feature genes were excluded from subsequent analysis. (B) Elbow-plot; PC number 14 was used for clustering for total cells. (C) Ductal cell cluster expressed high levels of *PROM1* (CD133) but low amount of *ITGA6* (CD49f), consistent to flow cytometry analysis (Fig 3B). (D) UMAPs showing the gene expression of the *SOX9*, *PDX1* or *NKX6.1* within the total cells. The majority of the detected cells are within the ductal cluster. (E-I) Quality control for StemID analysis. (E) RaceID2 clusters, connected by links. (F) Upper left is showing a heatmap of cell-to-cell transcriptome distances. The color of the dots indicates the –log10 p-value and the color of the vertices indicates the entropy. (G) The number of links per cluster, (H) median transcriptome entropy, and the (I) resulting StemID score are shown in bar graphs, with cluster 4 indicated by the red box having the highest value; this bar represents 418 cells.

Table S1: List of donor information, related to Figures 1, 2, 3, 4, 5, 6 and 7.

Donor tissue[1]	Age (years)	BMI (kg/m^2)	HbA1c (%)	Gender	Ethnicity	Figure #
1	16	23.5	5.3	Male	Caucasian	1c, 1d, 1e, 1g, 1h, 1i, 3f, 3g, 4e, 5b, 5e, 6b, 6c, 6d, 6e, 6f, s1c, s3c, s3d, s5a, s5b, s5c, s5d, s5f, s5g, s5i
2	25	25.3	5.3	Male	Caucasian	1c, 1d, 1e, 2h, s3f
3	26	24.6	5.1	Male	Caucasian	1c, 1d, 1e, 4b, 4c, 4d, 5e, s3g
4	52	31.5	n/a	Female	Caucasian	1c, 1d, 1e, 1h, 3f, 4e, s3c, s3d
5	52	32.4	5	Male	Hispanic	1c, 1d, 1e, 1h, 4e, s3c, s3d
6	24	24.6	5	Male	Caucasian	1c, 1f, 1i, 4b, 4c, 4d, 4e, 5b, 5c, 5d, 5g, 5h, 5i, 5j, 6b, 6c, 6d, 6e, 6h, 6i, s3g, s4a, s4b, s4c, s5a, s5b, s5c, s5d, s5f, s5g, s5i, s6b
7	27	31.4	5.4	Male	African American	1c, 2c, 2d, 2e
8	45	38	5.3	Female	Caucasian	1c, 1h, 2c, 2d, 2e, 2h, 3d, 3e, 3f, 4e, s1a, s1e, s3e
9	59	35.6	n/a	Female	Caucasian	1c, 3b, 3c, 3e
10	24	32	4.7	Male	Caucasian	1c, 3b, 3c, 3e
11	29	33.9	4.2	Male	Hispanic	1c, 3b, 3c, 3e, s3a, s3c, s3d
12	24	50.5	5.6	Male	Caucasian	1c, 3b, 3c, 3e, 3f, s3b
13	41	19.1	4.7	Female	Caucasian	1c, 3b, 3c, 3e
14	36	36.6	5.3	Male	Hispanic	1c, 3c, 3e, 5e, 5f, 5g, s4b
15	53	27.7	5.5	Female	Caucasian	3c, 3e, 4e
16	17	39.4	5.1	Male	Hispanic	1c, 3c, 3e
17	35	27.4	5.4	Male	Caucasian	1c, 4e, 5b, 5e, 5f, 6b, 6c, 6d, 6e, s5a, s5b, s5c, s5d, s5f, s5g, s5i
18	60	37.9	4.5	Male	Caucasian	1c, 5b, 5e, 5f, 5g, s4b
19	65	35.1	5.6	Female	African American	1c, 4b, 4c, 4d, 5b, 5e, 5f, 5g, 6b, 6c, 6d, 6e, 6g, 6j, 6k, s3g, s4b, s5a, s5b, s5c, s5d, s5f, s5g, s5i, s6b
20	23	20.6	5.5	Male	African American	1c, 1i, 5b, 5d, 5e, 5f, 5g, 5h, 5i, s1b, s4b
21	50	21.5	4.9	Female	Caucasian	1c, s3g
22	39	30	5	Male	Caucasian	1c, 4b, 4c, 4d, s3g
23	61	42.1	n/a	Male	Hispanic	1c, s3c, s3d
24	47	29.3	5.1	Male	Hispanic	1c, 1h, 4e
25	34	31.3	5.1	Male	Caucasian	1c, 1i, 5b, 5d, 5e, 5h, 5i, s4c
26	23	32.9	4.7	Male	Hispanic	1c, 1i, 5b, 5d, 5e, 5h, 5i, s4c
27	27	30	5.2	Male	Hispanic	1c
28	57	26	5.4	Female	Hispanic	1c, 5b
29	24	24	5.2	Female	Hispanic	1c, 5b, 5e, 5f
30	23	36.1	5.1	Male	Hispanic	1c, 7, s7
31	28	21	5.5	Male	Caucasian	1c
32	22	31.3	5.5	Male	Hispanic	1b, 1c
33	15	24.5	5.1	Male	Caucasian	1c
34	40	36	5	Female	Hispanic	1c
35	46	30	5.6	Female	Caucasian	1c
36	38	23.8	5.1	Male	Hispanic	1i, 5e, 5f, s1b
37	26	28.6	4.8	Male	Hispanic	3f
38	26	24.4	5.2	Male	Hispanic	5g, s4b
39	50	36.2	4.9	Male	Caucasian	2b, 2c, 2d, s1d

40	44	24.38	5.3	Male	n/a	2h
41	18	35.5	5.2	Male	n/a	2f, 2g, 2h, s1f, s2a, s2b
42	17	32.2	5.5	male	Caucasian	S5H
43	55	28.6	5.6	male	Caucasian	S5H
44	42	35	5.4	male	Hispanic	S5H

[1]: Donor numbers 1-41 are from exocrine tissues that were used for progenitor cell studies. Donor numbers 42-44 are from islets that were used as positive control for transplantation studies.

Table S2: Summary of donor characteristics on exocrine tissue (donor numbers 1-41), related to Figures 1, 2, 3, 4, 5, 6 and 7.

	Mean ± SD	Lower limit	Upper limit
Age (years)	36 ± 14	15	65
Body Mass Index (BMI) (kg/m^2)	30.4 ± 6.6	19.1	50.5
HbA1c (%)	5.1 ± 0.3	4.2	5.6
Gender: male	73%	n/a	n/a
Gender: female	27%	n/a	n/a
Ethnicity[1]: Caucasian	51%	n/a	n/a
Ethnicity[1]: Hispanic	41%	n/a	n/a
Ethnicity[1]: African American	8%	n/a	n/a

[1]: When available in documentation; n=39 donors

Table S3: List of cell culture components used for the standard colony assay, related to Figures 1, 2, 3, 4, 5, 6 and 7.

Name	Stock Concentration	Final Concentration	Company	Catalog number
Methylcellulose, 1500 cPs	3.3% in DMEM/F12	1%	Shin-Etsu	9004-67-5
Matrigel	100% (8-12 mg/ml)	5%	Corning	356231
KnockOut Serum Replacement	100%	10%	ThermoFisher	10828-028
Nicotinamide	1 M	10 mM	Sigma	N0636
Exendin 4	0.1 mM	0.1 nM	Sigma	E7144
SB 202190	100 mM	10 μM	Sigma	S7067
Gastrin II Sulfated	1 mM	100 nM	Sigma	G1260
rm R-Spondin 1	250 mg/ml	750 ng/ml	R&D	3474-RS
rh VEGF	10 mg/ml	10 ng/ml	R&D	293-VE
rh EGF	25 mg/ml	50 ng/ml	R&D	236-EG
rm Noggin	10 mg/ml	100 ng/ml	R&D	1967-NG
A 83-01	50 mM	500 nM	Tocris	2939
rh/m/r Activin B[1]	10 mg/ml	10 ng/ml	R&D	6905-AB

[1]: Activin B is included in culture media for Fig. 1, 2, 3, S1, S3, and Movie 1.

Table S4: List of primary antibodies used for immune-fluorescent staining, related to Figures 1, 2, 5 and 6.

Antibody	Company	Catalog Number	RRID
Hamster anti-Mucin	Neomarkers/ThermoFisher	1630-PI	AB_11000874
Rabbit anti-Amylase	Sigma	A8373	AB_258380
Guinea pig anti-PDX1	Abcam	Ab47308	AB_777178
Rabbit anti-Ghrelin	Phoenix	H-031-031	AB_2314558
Mouse anti-NEUROG3	DSHB	F25A1B3	AB_528401
Rabbit anti-Glut1	Abcam	Ab15309	AB_301844
Guinea pig anti-Insulin	Dako/Agilent	A0564	AB_10013624
Mouse anti-Glucagon	Sigma	G2654	AB_259852
Goat anti-Ecad	R&D Systems	AF748	AB_355568
Rabbit anti-KRT19	Abcam	ab52625	AB_2281020
Mouse anti-pan-CK	DAKO	M3515	AB_2132885
Rabbit anti-Urocortin III (Ucn3)	Phoenix Pharmaceuticals	H-019-28	AB_2889826
Rabbit anti-Hes1 (D6P2U)	Cell signaling	11988S	AB_2728766
Rabbit anti-Neurod2	Abcam	ab104430	AB_10975628
Rabbit anti-Nkx6-2	Thermofisher	PA5-103900	AB_2853232
Rabbit anti-Chromogranin A (ChrA)	Novus	NB120-15160	AB_789299
Goat anti-NKX6.1	Novus/R&D Systems	AF5857	AB_1857045
Rabbit anti-Sox9	Chemicon/Milipore-Sigma	AB5535	AB_2239761

Table S5: List of Taqman probes used for conventional and micro-fluidic qRT-PCR, related to Figures 1, 2, 3, 4, and 5.

Human gene	Assay ID
Beta-actin	Hs01060665_g1
B2-MG	Hs00984230_m1
RPLPO	Hs99999902_m1
KRT19	Hs00761767_s1
KRT7	Hs00818825_m1
MUC1	Hs00159357_m1
HNF1B	Hs01001602_m1
SOX9	Hs00165814_m1
PROM1 (CD133)	Hs01009259_m1
AMY2A	Hs00420710_g1
CPA1	Hs01056157_m1
HES1	Hs00172878_m1
SLC2A1 (GLUT1)	Hs00892681_m1
PTPRC (CD45)	Hs00236304_m1
KDR	Hs00176676_m1
INS	Hs00355773_m1 (microfluidic), Hs02741908_m1 (conventional)
GCG	Hs00174967_m1
PPY	Hs01078030_m1
SST	Hs00356144_m1
GHRL	Hs00175082_m1
PDX1	Hs00236830_m1
NEUROG3 (NGN3)	Hs01875204_s1
NEUROD1	Hs01922995_s1
NEUROD2	Hs00272055_s1
MAFB	Hs00534343_s1
NKX6.1	Hs00232355_m1
NKX6.2	Hs00752986_s1
UCN3	Hs00846499_s1

Supplemental Experimental Procedures:

Single-cell suspension. Donated pancreata were procured and shipped to City of Hope for isolation of islets as previously described (1). All tissues used in this study had consent for research from close relatives of the donors. After islet removal, de-identified human pancreata were obtained from the Southern California Islet Cell Resource (SC-ICR) Center at the City of Hope. The tissue was rinsed once in cold PBS, resuspended in Dulbecco's phosphate-buffered saline (DPBS) containing 0.1% bovine serum albumin (BSA), collagenase B (2-4 mg/ml) (Roche, Mannheim, Germany), and DNase I (2,000 U/ml) (Calbiochem, Darmstadt, Germany), and incubated at 37°C for 20 min, during which time the tissue was gently disrupted every 5-10 min using a 16G syringe needle. Cells were washed twice in DPBS / 0.1% BSA / 2,000 U/ml DNase I and filtered through a 40 μm nylon mesh (BD Biosciences, San Jose, CA) to yield a single-cell suspension before cryopreservation, culture, or antibody staining.

Freezing and thawing of single cells. Dissociated single-cell suspensions were counted, resuspended in Cryostor (BioLife Solutions), and frozen using a programed cell freezer before being transferred to a liquid nitrogen tank for long-term storage. To thaw, frozen vials were placed in a 37°C water bath for 2 minutes. Thawed cells were transferred to a 15 ml conical tube and washed once with warm PBS / 0.1% BSA before culture.

Flow cytometry and cell sorting. Cell suspensions were blocked with human gamma globulin (IgG) (22.8 μg/ml) (Jackson ImmunoResearch, West Grove, PA) for 15 min on ice. Biotin-conjugated anti-human CD133/2 (clone 293C3; Miltenyi Biotec) and fluorescein (FITC)-conjugated anti-human/mouse CD49f (clone GoH3; BioLegend) antibodies were added to cells. After 20 min on ice, cells were washed twice, treated with streptavidin-labeled allophycocyanin (APC) (2 μg/ml BioLegend, San Diego, CA) for 15 min on ice, washed twice, and resuspended in PBS / BSA / DNase I containing 4',6-diamidine-2'-phenylindole dihydrochloride (DAPI, 0.2 μg/ml). Control antibodies used were biotin-conjugated mouse IgG_{2b} (Clone eBMG2b; eBioscience) and FITC-conjugated rat IgG_{2a} (BioLegend). Cell sorting was performed on an Aria-special order research product (SORP) (Becton Dickinson, San Jose, CA). All analyses included an initial gating of forward (FSC) and side (SSC) scatters to exclude debris. Sorting further excluded doublets by gating out high pulse-width cells; live cells were selected by negative staining with DAPI. The purity of the sorted events was routinely > 95%. Acquired flow data were analyzed using software provided by FlowJo (TreeStar, Ashland, OR).

***In vitro* colony assay.** Cells were cultured at a density between 1×10^3 to 5×10^3 cells in 0.5 ml per well as previously described (2). The standard culture medium for adult human cells contained DMEM/F12, KnockOut Serum Replacement, methylcellulose, Matrigel, nicotinamide, Noggin, epidermal growth factor (EGF), A83-01, SB202190, Rspondin-1 (RSPO1), exendin-4, vascular endothelial growth factor-A (VEGF), gastrin II (sulfated), and activin B (see Table S3 for concentrations and sources). Because transforming growth factor (TGF)-beta signaling is agonized by activin B and antagonized by A83-01, we later determined that activin B was dispensable (data not shown). Cells were plated in 24-well ultra-low protein-binding plates (Corning, New York, USA) and incubated in a humidified 5% CO_2 atmosphere at 37°C. Addition of N-[N-(3,5-Difluorophenacetyl)-L-alanyl]- S-phenylglycine t-butyl ester (DAPT) on day 10 was accomplished by distributing 50 μL of DAPT solution per well on top of the semisolid medium. The number of colonies was counted 3 weeks post-plating. Percent PCFU was calculated by dividing the number of colonies formed by the total number of cells plated per well.

Analysis of colony size and cell number. Individual colonies were imaged using Infinity Analyze software (Lumenera Corporation) and colony diameters were measured. Colonies were assigned to a category (small, medium, or large; 10 colonies per group with 2 technical replicates), hand-picked, and pooled into wells in a 96-well plate. Colonies were dissociated into single cells by incubating with 0.25% trypsin-EDTA at 37°C for 5 min, followed by gentle pipetting. Single cells were counted using a hemocytometer.

Micro-manipulation of single colonies or cells. Three-week-old colonies were visualized under a microscope, lifted one-by-one using a 10-μL pipette tip in a volume of ~2 μL, and processed for microfluidic qRT-PCR analysis (Fig. 1H, analysis procedure described below) (2). To micro-manipulate single cells, using a method similar to reported by Suda et al. (3), cells were first placed in a semisolid

medium in DMEM/F12 with 1% methylcellulose and 10% Knockout Serum at a density of 3,000 cells per ml in a 35-mm petri dish. Individual cells were visualized under a microscope and lifted one-by-one using a fine Pasteur pipet with a diameter of ~30 µm at the opening. Each cell was transferred into one well in a low-binding 96-well plate containing 100 µL of standard semisolid medium (Fig. 2A). The presence of a single colony was confirmed by visualization under a microscope. In some experiments, micro-manipulated single cells were processed for microfluidic qRT-PCR analysis (Fig. 3D).

In vitro self-renewal assay. Cells were plated into our standard colony assay (1° culture). Jag1/Fc (R&D Systems #1277), a Notch ligand, or Y-27632 (Stemgent #04-0012-10), a ROCK inhibitor, was added at a concentration of 1 µg/ml or 10 µM, respectively. Three weeks later, colonies were collected, pooled, washed in warm PBS/0.1% BSA, and dissociated into single cells by incubating with 0.25% trypsin-EDTA at 37°C for 5 min, followed by gentle pipetting. Single cells were counted, diluted, and a fraction of the cells were re-plated into a new 24-well plate at a concentration of 5×10^3 cells in 0.5 ml per well (2° culture). This pooling, dissociation, and re-plating was repeated once more into 3° culture. The final total number of PCFUs was calculated by multiplying the previous dilution factor(s) with the number of colonies per well in the present culture. The fold change of PCFUs was calculated by the total number of PCFUs in the culture divided by the total number of PCFUs in the 1° culture.

***In vivo* transplantation.** Three-week-old colonies that were treated with 10 µM DAPT on day 10 were collected on day 21, pooled, partially digested in 0.063% trypsin-EDTA at 37°C for 3 min, gently pipetted, washed, and concentrated (4). An aliquot was further dissociated into single cell suspension and counted with a hemocytometer to estimate cell number per graft. Three weeks before transplantation, NOD-SCID mice (8-week-old males) were injected with streptozotocin (STZ; 45 mg/kg body weight; freshly made in 100 mM Na-citrate buffer, pH 4.5) for 3 consecutive days to induce insulin-dependent diabetes (5). Hyperglycemia was defined as fasting blood glucose >200 mg/dl and was measured using a HemoCue Glucose 201 (HemoCue). Approximately 3,500 colonies (1-2.5 $\times 10^6$ cells) were placed under the renal capsule for each mouse; colonies from a single donor were transplanted into 2 to 4 mice (n=15 mice from 4 donor tissues). Fasting blood glucose was monitored weekly; mice were placed in a new cage without food and blood glucose measured 5 hours later. When a mouse had a blood glucose level higher than 450 mg/dL an insulin pellet was inserted within the first 60 days. Data collection on function of the beta cells occurred after 90 days to allow for a washout of the exogenous insulin.

Colonies from four different donor tissues were transplanted into mice in independent experiments. Control mice were age-matched, received STZ injections, and were subjected to sham operations. Mice with the top 8 median blood glucose were separated from the bottom 7, representing the non-responder and responder transplants, respectively.

***In vivo* glucose-stimulated insulin secretion (GSIS).** Mice were fasted for 6 hours in a clean cage before the first blood collection. D-glucose was then given intraperitoneally at 2 g/kg to each mouse, and blood serum was collected after 60 minutes. Concentrations of C-peptide were measured using the human C-peptide Ultrasensitive ELISA kit (Mercodia, Winston Salem, NC). The amount of C-peptide secretion was expressed as fold-change of C-peptide concentration before and after glucose bolus, from the same mouse.

***In vivo* intra-peritoneal glucose tolerance test (IP-GTT).** Levels of blood glucose were measured at 0, 15, 30, 60, 90, and 120 min using a HemoCue after an i.p. injection of D-glucose (2 g/kg) to a mouse that had fasted for 6 hours in a clean cage.

Immunofluorescence staining. For paraffin-embedded formalin-fixed graft tissues, samples were cut to 5 µm thickness, de-waxed in xylene and rehydrated in ethanol. Antigen retrieval was performed by heating samples in a microwave oven for 10 min in 200 ml of sodium citrate buffer, 6.0 pH (Vector Laboratories, H-3300). Samples were incubated with blocking buffer supplemented with 5% donkey serum and 0.1% Triton X-100 at for 2 hours at room temperature. Primary antibodies, as listed in Table S4, were added and samples were incubated at 4°C overnight**.** Slides were washed with PBS/0.1% Triton X-100 and incubated with donkey-raised secondary antibodies conjugated to Cy3, Cy5, DyLight488, AlexaFluor488, or AlexaFluor647 (Jackson ImmunoResearch), for 2 hours at room temperature.

Autofluorescence was minimized using TrueView kit (Vector Laboratories, SP-8400-15) and mounted with Vectashield vibrance antifade mounting medium (Vector Laboratories, H-1700)

For whole-mount immunofluorescent staining, colonies were collected, pooled, and incubated in Cell Recovery Solution (Corning, 354253) for 30 min on ice, fixed in 4% paraformaldehyde with 0.15% Triton-X 100x at 4°C overnight, and incubated with blocking buffer, primary and secondary antibodies as described above.

For frozen sections, colonies were collected, pooled, and incubated with Cell Recovery Solution for 30 minutes on ice. Next, colonies were fixed in 4% paraformaldehyde with 0.15% Triton X-100 at 4°C overnight. Colonies were then cryoprotected in 30% sucrose with PBS at 4°C overnight, embedded in Optimal Cutting Temperature compound (Fisher Scientific, 23-730-571), and frozen into blocks. Samples were cut into 8 µm thickness and thawed at room temperature; thawed samples were washed with PBS. Antigen retrieval was necessary for anti-NEUROG3 (at pH 5.5) as well as anti-NEUROD2 and anti-UCN3 (at pH 8.0) detection. For antibodies with high background, slides were blocked with buffer containing 5% donkey serum (DS) or 10% DS + 1x Universal Blocking Reagent (BioGenex #HK085-5K), and 0.1% Triton X-100 for 1 hour at room temperature. Next, slides were incubated with primary and secondary antibodies as described above.

Images were captured on a Zeiss LSM880 with Airyscan, Zeiss Axio-Observer-Z1 with Apotome, or Zeiss Axio-Cam506 Mono for tiling images. Figures were prepared using LSM Image Browser software (Carl Zeiss, Germany) and Photoshop (Adobe). Quantification of images was performed with QuPath software to identify total cells (DAPI) and positive cell staining (6).

Conventional or microfluidic quantitative reverse transcription-polymerase chain reaction (qRT-PCR). Total RNA extraction, reverse transcription, and conventional qRT-PCR analyses were performed as previously described (7). β-actin was used as an internal control for normalization. Three technical replicates were used in all PCR runs.

Microfluidic qRT-PCR was performed using the BioMark 48.48 Dynamic Array system (Fluidigm-Biomark). Individual micro-manipulated cells or colonies were collected in 10 µL of reaction buffer (CellsDirect One-Step qRT-PCR Kit, Invitrogen, Carlsbad, CA) and preamplified (14 cycles for single colonies or 22 cycles for single cells) according to the manufacturer's instructions (Fluidigm, Invitrogen). Amplified cDNA was loaded onto a 48.48 Dynamic Array using the NanoFlex integrated fluidic circuit (IFC) controller (Fluidigm). Threshold cycle (Ct) was used to measure fluorescence intensity. Ct values were determined by the BioMark PCR analysis software (Fluidigm) and expressed either as a heat map or relative expression (delta Ct), of the gene of interest to internal control gene. In a heat map, the cooler and warmer colors represent lower and higher expression, respectively. All reactions were performed with negative (water) and positive (cDNA from adult human pancreatic cells) controls in all experiments. Taqman probes (Life Technologies, Carlsbad, CA) and their catalog numbers are listed in Table S5. Gene expression frequency in colonies (%) was obtained from the number of colonies expressing a gene divided by the total number of colonies examined. Also, the 'endocrine' category is the percent of colonies expressing either *INS*, *GCG*, *SST*, *PPY* or *GHRL* gene.

Electron microscopy. Single colonies were collected, pooled, and fixed in 2% glutaraldehyde in 0.1 M cacodylate buffer [$Na(CH_3)_2AsO_2 \cdot 3H_2O$; pH7.2] at 4°C overnight. Colonies were washed with 0.1 M cacodylate buffer (pH 7.2), post-fixed with 1% OsO_4 in 0.1 M cacodylate buffer for 30 min, and washed with 0.1 M cacodylate buffer. Samples were dehydrated, embedded in EMbed 812 (Electron Microscopy Sciences, Hatfield, PA), and polymerized at 64°C for 48 h. Ultrathin sections (70-nm thickness) were cut using a Leica ultramicrotome with a diamond knife, transferred to 200-mesh EM grids, and stained with 2% uranyl acetate in 70% ethanol for 1 min followed by Reynold's lead citrate for 1 min. Electron microscopy was performed on an FEI Tecnai 12 transmission electron microscope (ThermoFisher Scientific, Waltham, MA) equipped with a Gatan Ultrascan 2K CCD camera operated at 120 keV.

Three-dimensional scanning electron microscopy. Serial block-face scanning electron microscopy (SBF-SEM) was performed as described (8) with minor modifications. Colonies were manually picked and immediately fixed overnight at 4°C in 2.5% glutaraldehyde containing 2 mM calcium chloride. Subsequently, colonies were collected by centrifugation, rinsed in 0.15 M cacodylate buffer (pH 7.4) containing 2 mM calcium chloride, and post-fixed for 1 hour on ice in 0.15 M cacodylate buffer containing 2 mM calcium chloride, 1.5% potassium ferrocyanide, and 2% osmium tetroxide. Samples were rinsed in

distilled water and treated with 0.1% thiocarbohydrazide for 20 min at room temperature and rinsed in distilled water. Samples were treated with 2% osmium tetroxide for 30 min, rinsed in distilled water, dehydrated in an ethanol series, and infiltrated with Durcupan ACM resin. The sample block was mounted on an aluminum pin and trimmed to 0.5 mm × 0.5 mm. The specimen was placed in a field-emission scanning electron microscope (Zeiss Sigma VP) equipped with a serial block-face sectioning unit (Gatan 3View2XP). A backscattered electron image of the face was obtained under an accelerating voltage of 4 keV and a chamber pressure of 20 Pa in a variable pressure mode. An automatic microtome removed a 70 nm-thick slice from the face of the block and another image was recorded. Image sets (~500 slices) were collected to capture an entire cell in a colony. Individual cells in the image sets were segmented and color-rendered; three-dimensional reconstructions of the segmented single cells were derived using the Amira software (ThermoFisher Scientific). The area of vesicles containing insulin-like granules was determined using Image J (9).

Single-cell RNA sequencing. Fresh exocrine tissue was dissociated to single cells, counted, and diluted to the manufacturer's recommended concentration in 1X PBS supplemented with 0.1% BSA. An estimated 14,822 cells were captured on a 10x Chromium device using a 10X V3 Single Cell 3' Solution kit (10x Genomics, Chromium Single Cell 3' Regent kit V3 Chemistry, Cat. PN-1000092). All protocols were performed following the manufacturer's instructions. Final sequencing libraries were analyzed on a High Sensitivity DNA Chip (Agilent, Cat 5067-4626) to determine library size; final library concentrations were determined using a Qubit High Sensitivity DNA Assay Kit (Thermo). Libraries were sequenced using the paired-end setting of 101-101 with 8 cycles of index reads on an Illumina NovaSeq 6000 platform. Approximately 0.1 million reads per cell were sequenced.

Data analysis for single-cell RNA sequencing. Raw sequencing data from the biological sample was aligned to the human genome (hg19) using the CellRanger count command to produce expression data at a single-cell resolution, according to 10x Genomics. The R package Seurat (10) was used for gene and cell filtration, normalization, principal component analysis, variable gene finding, clustering analysis, and Uniform Manifold Approximation and Projection (UMAP) dimension reduction. Briefly, a matrix containing gene-by-cell expression data was imported to create individual Seurat objects for the sample. Cells with <200 detectable genes, >5,000 featured genes, and >15% mitochondrial genes were excluded, resulting in 7,812 cells. Principle component analysis of all cells identified sub-populations, which were visualized using UMAP data dimension reduction into 2D clusters. Violin plots were used to present gene expression levels of cells for each cluster. Another round of unbiased principle component analysis of the ductal cell cluster further distinguished unique sub-populations.

The R packages RaceID3 and StemID2 (11) were used to detect rare cells within the Seurat-identified ductal cluster, that correspond to outliers in conventional clustering methods. These algorithms require a gene-by-cell expression matrix as input and produce a clustering partition representing cell types. Within the ductal cluster, *SOX9*, *PDX1*, *NKX6.1* triple-positive (TP) cells were separated from the non-TP cells, which include cells that expressed 1 or 2 of these transcription factors. Differentially expressed genes between these two groups were determined using the FindAllMarkers function in Seurat. Ingenuity Pathway Analysis (Qiagen) was performed on differentially expressed genes ($P<0.05$ and no limit set for log2 fold change) among the four ductal clusters (Data S1), TP cells compared to non-TP ductal cells (Data S1), and StemID cells compared to non-StemID ductal cells (Data S1). The identified upregulated canonical pathways (with a z-score >1.1) or downregulated canonical pathways (with a z-score <-1.1) were subsequently sorted based on –log(p-value). The top 5-10 pathways are presented.

Statistical Analysis. All values are shown as mean or median ± standard error of the mean (SEM) or standard deviation (SD). Unless otherwise specified, Student's *t*-test with Welch's correction was used between two groups, and ordinary one-way ANOVA, with Dunnett's multiple comparison, was used when there were more than two groups to determine statistical significance, with $p<0.05$ considered significant.

References:

1. Qi M, Valiente L, McFadden B, Omori K, Bilbao S, Juan J, et al. The Choice of Enzyme for Human Pancreas Digestion is a Critical Factor for Increasing the Success of Islet Isolation. Transplantation direct. 2015 May;1(4). PubMed PMID: 26146662. Pubmed Central PMCID: 4486320.
2. Tremblay JR, LeBon JM, Luo A, Quijano JC, Wedeken L, Jou K, et al. In Vitro Colony Assays for Characterizing Tri-potent Progenitor Cells Isolated from the Adult Murine Pancreas. Journal of visualized experiments : JoVE. 2016 Jun 10(112). PubMed PMID: 27340914. Pubmed Central PMCID: 4927776.
3. Suda T, Suda J, Ogawa M. Single-cell origin of mouse hemopoietic colonies expressing multiple lineages in variable combinations. Proceedings of the National Academy of Sciences of the United States of America. 1983 Nov;80(21):6689-93. PubMed PMID: 6579554. Pubmed Central PMCID: 391236.
4. Quijano JC, Tremblay JR, Rawson J, Ku HT. Isolation and Characterization of Colony-Forming Progenitor Cells from Adult Pancreas. Methods in molecular biology. 2019;2029:63-80. PubMed PMID: 31273734.
5. Gerling IC, Friedman H, Greiner DL, Shultz LD, Leiter EH. Multiple low-dose streptozocin-induced diabetes in NOD-scid/scid mice in the absence of functional lymphocytes. Diabetes. 1994 Mar;43(3):433-40. PubMed PMID: 8314017.
6. Bankhead P, Loughrey MB, Fernandez JA, Dombrowski Y, McArt DG, Dunne PD, et al. QuPath: Open source software for digital pathology image analysis. Scientific reports. 2017 Dec 4;7(1):16878. PubMed PMID: 29203879. Pubmed Central PMCID: 5715110.
7. Jin L, Feng T, Shih HP, Zerda R, Luo A, Hsu J, et al. Colony-forming cells in the adult mouse pancreas are expandable in Matrigel and form endocrine/acinar colonies in laminin hydrogel. Proceedings of the National Academy of Sciences of the United States of America. 2013 Mar 5;110(10):3907-12. PubMed PMID: 23431132. Pubmed Central PMCID: 3593860.
8. West JB, Fu Z, Deerinck TJ, Mackey MR, Obayashi JT, Ellisman MH. Structure-function studies of blood and air capillaries in chicken lung using 3D electron microscopy. Respiratory physiology & neurobiology. 2010 Feb 28;170(2):202-9. PubMed PMID: 20038456. Pubmed Central PMCID: 2821748.
9. Schneider CA, Rasband WS, Eliceiri KW. NIH Image to ImageJ: 25 years of image analysis. Nature methods. 2012 Jul;9(7):671-5. PubMed PMID: 22930834. Pubmed Central PMCID: 5554542.
10. Satija R, Farrell JA, Gennert D, Schier AF, Regev A. Spatial reconstruction of single-cell gene expression data. Nature biotechnology. 2015 May;33(5):495-502. PubMed PMID: 25867923. Pubmed Central PMCID: 4430369.
11. Grun D, Muraro MJ, Boisset JC, Wiebrands K, Lyubimova A, Dharmadhikari G, et al. De Novo Prediction of Stem Cell Identity using Single-Cell Transcriptome Data. Cell stem cell. 2016 Aug 4;19(2):266-77. PubMed PMID: 27345837. Pubmed Central PMCID: 4985539.
