# Supplementary material for: Methylcellulose colony assay and single-cell micro-manipulation reveal progenitor-like cells in adult human pancreatic ducts
Source: Stem Cell Reports. 2023 Mar 2;18(3):618–35. doi: 10.1016/j.stemcr.2023.02.001 (PMC10031308; doi:10.1016/j.stemcr.2023.02.001)
Supplement: Document S2. Article plus supplemental information [file mmc4.pdf]

Stem Cell Reports
Article

OPEN ACCESS

Methylcellulose colony assay and single-cell micro-manipulation reveal progenitor-like cells in adult human pancreatic ducts

Janine C. Quijano,[1,7,*] Lena Wedeken,[1,7,8] Jose A. Ortiz,[1,2] Heather N. Zook,[1,2] Jeanne M. LeBon,[1] Angela Luo,[1] Jeffrey Rawson,[1] Jacob R. Tremblay,[1] Jacob M. Mares,[1] Kassandra Lopez,[1] Min-Hsuan Chen,[3] Kevin Jou,[1] Carlos Mendez-Dorantes,[2] Ismail H. Al-Abdullah,[1] Debbie C. Thurmond,[4] Fouad Kandeel,[1,5] Arthur D. Riggs,[6] and Hsun Teresa Ku[1,2]

[1]Department of Translational Research & Cellular Therapeutics, City of Hope, 1500 E. Duarte Road, Duarte, CA 91010, USA
[2]Irell and Manella Graduate School of Biological Sciences, City of Hope, Duarte, CA 91010, USA
[3]Integrative Genomics Core, City of Hope, Duarte, CA 91010, USA
[4]Department of Molecular & Cellular Endocrinology, City of Hope, Duarte, CA 91010, USA
[5]Department of Clinical Diabetes, Endocrinology & Metabolism, City of Hope, Duarte, CA 91010, USA
[6]Department of Diabetes & Drug Discovery, City of Hope, Duarte, CA 91010, USA
[7]These authors contributed equally
[8]Present address: CELLphenomics GmbH, Robert-Roessle-Str. 10, 13125 Berlin, Germany
*Correspondence: jquijano@coh.org
https://doi.org/10.1016/j.stemcr.2023.02.001

SUMMARY

Progenitor cells capable of self-renewal and differentiation in the adult human pancreas are an under-explored resource for regenerative medicine. Using micro-manipulation and three-dimensional colony assays we identify cells within the adult human exocrine pancreas that resemble progenitor cells. Exocrine tissues were dissociated into single cells and plated into a colony assay containing methylcellulose and 5% Matrigel. A subpopulation of ductal cells formed colonies containing differentiated ductal, acinar, and endocrine lineage cells, and expanded up to 300-fold with a ROCK inhibitor. When transplanted into diabetic mice, colonies pre-treated with a NOTCH inhibitor gave rise to insulin-expressing cells. Both colonies and primary human ducts contained cells that simultaneously express progenitor transcription factors SOX9, NKX6.1, and PDX1. In addition, *in silico* analysis identified progenitor-like cells within ductal clusters in a single-cell RNA sequencing dataset. Therefore, progenitor-like cells capable of self-renewal and tri-lineage differentiation either pre-exist in the adult human exocrine pancreas, or readily adapt in culture.

INTRODUCTION

Progenitor cells are distinguished by their ability to both self-renew and differentiate. These cells have been identified in many adult organs and can maintain tissue homeostasis and initiate repair of injuries. In the adult pancreas, there are three major cell lineages: ductal, acinar, and endocrine cells that include insulin-secreting beta cells. Studies of mouse embryos revealed that, in early (<E12.5) pancreas development, multi-potent progenitor cells (MPCs), expressing *Sox9*, *Pdx1*, and *Nkx6.1* (Gu et al., 2002; Kopp et al., 2011; Nelson et al., 2007), can give rise to these three lineages *in vivo* (Gu et al., 2002; Kopp et al., 2011) as well as *in vitro* using a three-dimensional (3D) culture assay (Greggio et al., 2013). Using *in vivo* lineage-tracing strategies, some studies found that in adult mice ductal cells can also give rise to beta cells (Al-Hasani et al., 2013; Dirice et al., 2019; Gribben et al., 2021; Inada et al., 2008; Xu et al., 2008), while others refuted these findings (Kopp et al., 2011; Solar et al., 2009; Zhao et al., 2021).

Although *in vivo* studies remain inconclusive, the use of certain 3D cultures has shown that some of the adult murine ductal cells self-renew and differentiate *in vitro* (Dorrell et al., 2014; Huch et al., 2013). For example, the 3D organoid assay established by Huch et al. (2013) showed that dissociated adult murine ductal cells and duct fragments can differentiate into endocrine and ductal, but not acinar, cell lineages in the presence of high concentrations of Matrigel. In contrast, the 3D colony assay system developed by our laboratory (Jin et al., 2013) uses methylcellulose, a biologically inert and viscous material, which allows us to lower Matrigel concentration to 5% v/v and detect tri-lineage differentiation. In a methylcellulose-containing semi-solid medium, cells cannot move and aggregate. Following the tradition of hematologists who call hematopoietic progenitor cells grown in a methylcellulose-containing culture medium "colony-forming units," we named a pancreatic progenitor cell capable of giving rise to a colony a pancreatic colony-forming unit (PCFU). Using this system, quantifying colony-forming progenitor cells can be done with relative ease.

In cadaveric human pancreatic ducts, previous reports have identified progenitor-like cells that are capable of duct and endocrine differentiation and some expansion (Bonner-Weir et al., 2000; Georgakopoulos et al., 2020; Lee et al., 2013; Loomans et al., 2018; Qadir et al., 2018). However, no human study of pancreas tissue thus far has utilized micro-manipulation of a single cell or colony to address lineage potential or lineage composition, respectively. Micro-manipulation is a technique that utilizes tools

This is an open access article under the CC BY license (http://creativecommons.org/licenses/by/4.0/).

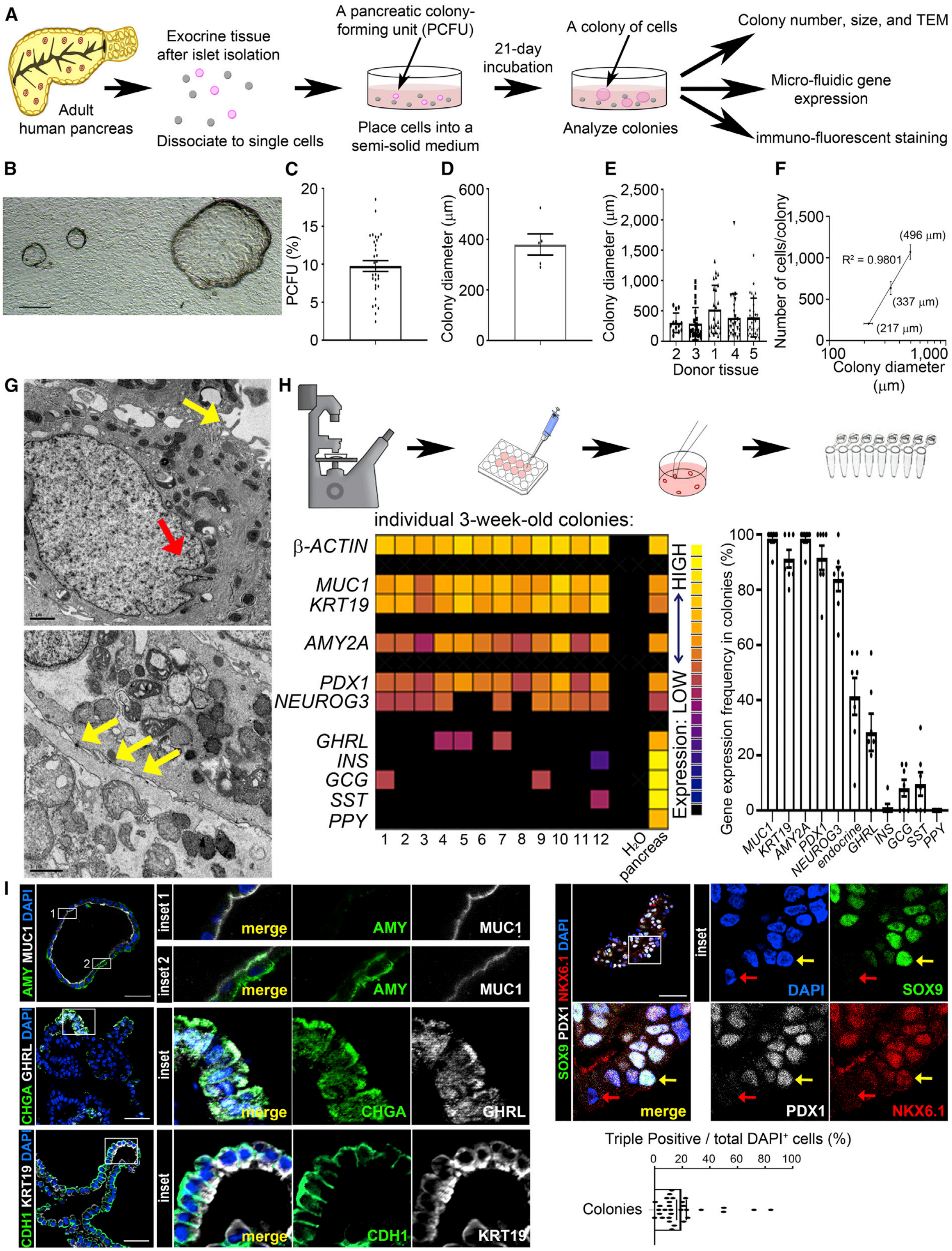

(legend on next page)

such as a pipette with a narrow opening to aspirate a cell or a colony of interest, one at a time, for subsequent downstream analysis (Tremblay et al., 2016). Such a clonal analysis is critical to ascertain multi-potency because a population of uni-potent progenitor cells for different lineages may collectively appear to be multipotent. Thus, despite the advances made in the aforementioned studies, no definitive evidence exists yet to demonstrate self-renewal and tri-lineage differentiation of adult human pancreatic progenitor cells. In this study, we describe a human colony assay system that reveals the self-renewal and tri-lineage differentiation abilities of an adult human ductal subpopulation. Single-cell RNA sequencing (scRNA-seq) analysis on dissociated exocrine cells confirms ductal cell heterogeneity, with a sub-cluster expressing genes consistent with progenitor cell phenotype.

RESULTS

Establishment of a methylcellulose-based colony assay for adult human PCFUs

We studied pancreatic exocrine tissues, which include ductal and acinar cells, from 41 cadaveric human donors without apparent disease (Table S1). These donors had an average age of 36 ± 14 years, body mass index of 30.4 ± 6.6 kg/m^2, and hemoglobin A1c of 5.1 ± 0.3% (Table S2). After islets were isolated, the pancreas tissue was dissociated into a single-cell suspension and was either cryopreserved or immediately plated into our colony assay system (Figure 1A). Our "standard" culture medium (Table S3) contains methylcellulose (1% w/v), non-defined extracellular matrix proteins (Matrigel; 5% v/v) (Jin et al., 2013), and defined soluble factors (Nicotinamide, EGF, Noggin, Exendin4, SB202190, Gastrin, RSPO1, VEGF, and A83-01) that were inspired by culture conditions for adult human gastrointestinal stem cells (Bartfeld et al., 2015; Sato et al., 2011) and adult murine ductal progenitor cells (Huch et al., 2013; Wedeken et al., 2017). Using this colony assay system, we achieved 100% isolation efficiency from every human exocrine tissue obtained to date, in contrast to 75%–80% shown by others (Boj et al., 2015).

After 3 weeks of culture morphologically distinct colonies formed, mostly appearing as hollow spheres (Figure 1B). The % PCFU, or colony-forming efficiency, varied among different donors with an average of 9.8% ± 0.7% (n = 31; range 2.4%–18.6%) (Figure 1C). The mean diameter of a colony was 380 ± 42 μm (Figure 1D), with individual donors showing high variability of sizes (Figure 1E). Colonies were segregated into small, medium, and large categories (10 colonies each) and were dispersed into single cells to quantify the number of cells per category. We observed a strong positive correlation between colony size and cell number (Figure 1F; R^2 = 0.98), indicating that colony size is predictive of the number of cells in that colony. The colony size can be indicative of the proliferative potential of the originating PCFU. Alternatively, the differences in colony size may be due to variable delay of cells entering replication or time needed for differentiation.

Transmission electron microscopy (TEM) revealed that cells in colonies displayed microvilli facing the lumen (Figure 1G), suggesting apical polarization. Cells had nuclear invaginations and desmosomes at cell-cell junctions. These results indicate that a colony is composed of duct-like cells. Furthermore, 3D scanning electron microscopy (3D-SEM) analysis clarified that the walls of the colonies contained individual cells that were flat and elongated (Video S1). Also, microvilli were facing lumen and nuclei contained invaginations (Figure S1C); confirming that most cells in a colony are ductal and exhibit apical-basal polarity.

The lineage potential of a PCFU is reflected in colonies expressing markers for various lineages. To determine the

Figure 1. Methylcellulose-based colony assay for adult human pancreatic progenitor cells capable of tri-lineage differentiation
(A) Experimental diagram.
(B) Representative bright-field image of colonies. Scale bar, 200 μm.
(C) % PCFUs in dissociated exocrine tissues is 9.8% ± 0.7% (N = 31 donors).
(D) Colony diameter = 394 ± 37 μm; mean ± SEM, ≥10 colonies per donor, N = 6 donors with 4 technical replicates.
(E) Diameters of colonies between different donors (N = 5).
(F) Mean diameter of colonies is positively correlated with the total number of cells per colony (R^2 = 0.9801); mean ± SEM from 2 independent experiments and 20 individual colonies per data point.
(G) TEM of 3-wo colonies displaying microvilli on the apical side (top, yellow arrow), nuclear invaginations (top, red arrow), and desmosomes (bottom, yellow arrows). Scale bars, 1 μm.
(H) Micro-manipulation of individual colony for microfluidic qRT-PCR. Representative heatmap of lineage markers; n = 58 colonies, N = 7 donors. Gene expression frequency; mean ± SD.
(I) IF staining confirms protein expression. Scale bar, 50 μm (insets enlarged 4×). Yellow arrow points to a representative cell that is triple-positive (TP) for SOX9, PDX1, and NKX6.1 and a red arrow for a non-TP cell. TP quantification represents mean ± SEM (19.5% ± 3.5%) from a total of 31 colonies from N = 3 donors. See also Figure S1B.

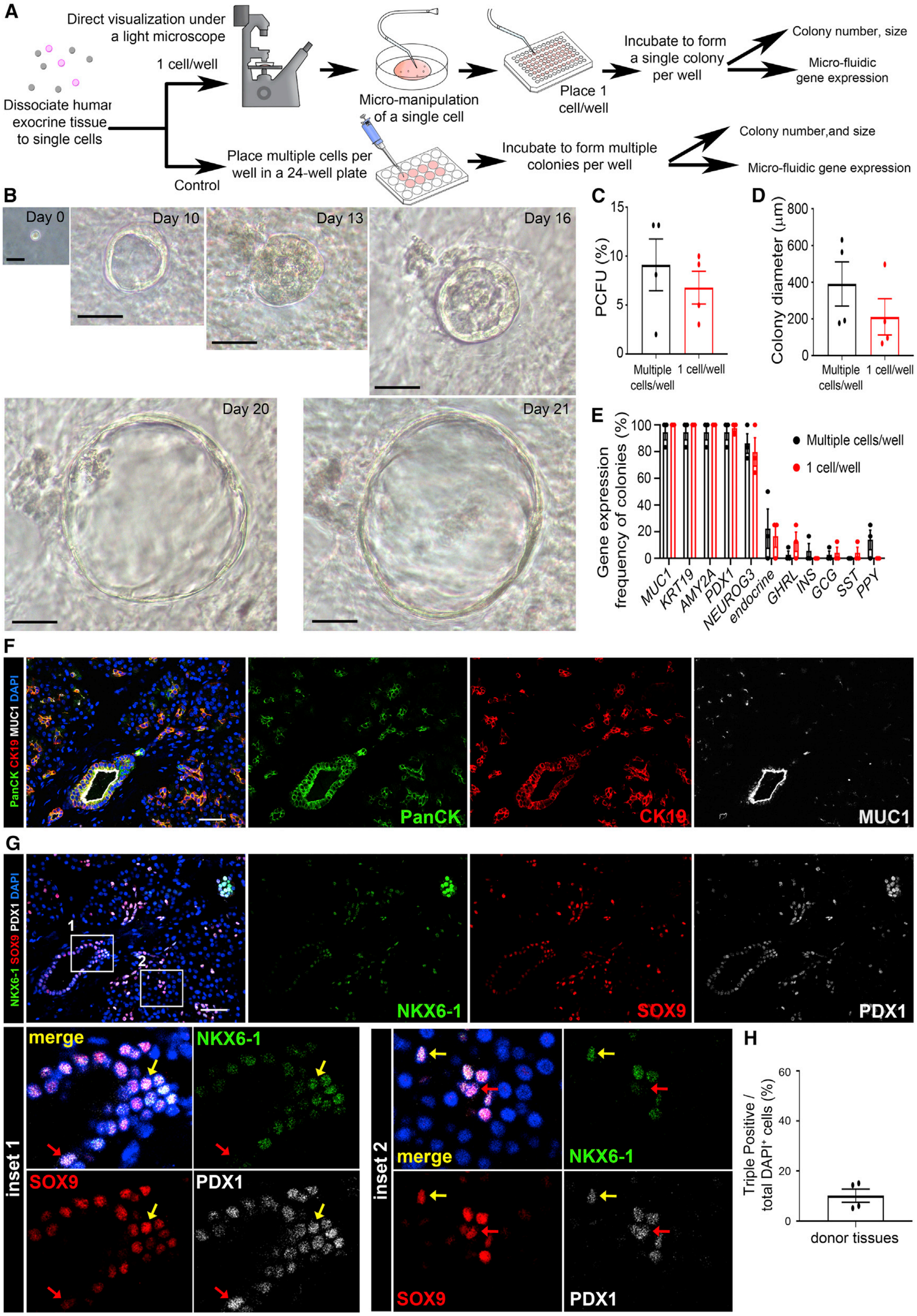

(legend on next page)

lineage potential of each PCFU, we micro-manipulated each 3-week-old (3-wo) colony by identifying it under a light microscope, picking the colony up with a pipette and placing that volume into a microcentrifuge tube (Figure 1H) pre-loaded with reagents for microfluidic qRT-PCR analysis (Jin et al., 2013). All individual colonies expressed high levels of markers for ductal (*MUC1*, *KRT19*, *KRT7*, *HNF1B*, *SOX9*, and *PROM1*), and multi-potent and endocrine progenitor cells (*PDX1*, *NEUROG3*, *NEUROD2*, *MAFB*, and *NKX6.1*; Figures 1H and S1A). The acinar cell marker *AMY2A* was also consistently expressed by all colonies. In contrast, the frequency of colonies that displayed at least one of the combined five endocrine markers (*INS*, *GCG*, *PPY*, *SST*, and *GHRL*) was only 41.7% ± 15.5%, with ghrelin being the most frequent (30.6% ± 16.9%). Because all colonies expressed markers for ductal and acinar lineages, and 41.7% of colonies expressed combined endocrine markers, these results demonstrate that approximately 40% of adult human PCFUs are tri-potent. The lower expression of *INS* in colonies at this stage reflects suboptimal culture conditions rather than a lack of lineage potential, as is shown later in the transplantation study.

Immunofluorescence (IF) staining verified protein expression of MUCIN1, KRT19, amylase, ghrelin, chromogranin A (a pan-endocrine marker), and CDH1 (a pan-epithelial marker) in 3-wo colonies (Figures 1I and S1B). MUCIN1 was detected at the surface of cells facing the lumen, confirming apical polarization. Because the transcription factors PDX1, SOX9, and NKX6.1 are known markers for the mouse and human embryonic MPCs (Jennings et al., 2013), we co-stained for these markers. On average, one-fifth of the cells in 3-wo colonies were triple-positive (TP) for $SOX9^+$/$PDX1^+$/$NKX6.1^+$ (Figure 1I, 19.5% ± 3.5%), demonstrating that a subset of cells within colonies display a progenitor cell phenotype.

A micro-manipulated single PCFU is sufficient to give rise to a 3-wo colony expressing the three major pancreatic lineages

To further ascertain the tri-lineage potential of PCFUs, we micro-manipulated freshly dissociated cells before culture by identifying single cells under a microscope and placing them into a 96-well plate at 1 cell per well (Figure 2A). Cells from the same donor were also plated into a standard colony assay as a control. Tracking each well of the 96-well plate for 3 weeks confirmed that a colony originated from one cell (Figures 2B and S1D). Compared with the control colonies, colonies derived from micro-manipulated single cells showed no significant difference in % PCFU (Figure 2C) and diameter (Figure 2D), suggesting that the formation of a colony is cell autonomous.

Microfluidic qRT-PCR analysis on individual 3-wo colonies demonstrated that the frequency of colonies expressing tri-lineage markers was similar between colonies derived from plating with single vs. multiple cells per well (Figures 2E and S1E). Overall, a total of 306 micro-manipulated single cells from 3 independent experiments resulted in an average of 6.0 ± 2.1% colony formation. Of those colonies, more than 15% gave rise to 3-wo colonies expressing duct, acinar, and endocrine lineage markers (example colonies nos. 3 and 7 in Figure S1E), confirming the tri-potency of those PCFUs.

Endogenous ducts contain TP cells

The presence of TP cells in 3-wo colonies (Figure 1I) and the tri-potency of individually micro-manipulated PCFUs (Figure 2E) prompted us to examine the existence of TP cells in the adult human pancreas. Large and small ducts were identified by pan-CK, CK19, and MUC1 staining (Figures 2F and S2A). In the sequential slide, TP cells were identified in ductal areas (Figure 2G, yellow arrows;

Figure 2. A micro-manipulated single PCFU is sufficient to give rise to a colony expressing the three major pancreas lineages, and identification of $SOX9^+$/$PDX1^+$/$NKX6.1^+$ cells in endogenous ducts

(A) Experimental diagram.

(B) Time course bright-field imaging of a single PCFU grown into a colony. Scale bar, 20 μm (on day 0) and 50 μm (for all other days).

(C and D) (C) % PCFUs and (D) mean diameter of colonies grown from 1 cell per well vs. multiple cells per well; mean ± SEM from 4 independent experiments using 3 donor tissues. Paired t test determined significance.

(E) Microfluidic qRT-PCR analysis of colonies grown from unsorted cells plated at multiple cells per well (black) (n = 24 colonies, 3 independent experiments from 2 donors), vs. 1 cell per well (red) (n = 22 colonies); mean ± SD. Significance was determined by two-way ANOVA with Sidak's multiple comparison.

(F) IF staining of Pan-CK (green), CK19 (red), and MUC1 (white) in human pancreas. The image contains interlobular, intralobular, and intercalated ducts. Scale bar, 50 μm.

(G) The same region in a sequential slide to (F) is stained with NKX6.1 (green), PDX1 (white), and SOX9 (red). Insets 1 and 2 highlight an interlobular and an intercalated duct, respectively; both contained TP cells (yellow arrows point to representatives of $SOX9^+PDX1^+NKX6.1^+$ cells) and non-TP cells (red arrow points to representative of a $SOX9^+PDX1^+NKX6.1^-$ cell). Scale bar, 50 μm (insets enlarged 4×).

(H) Quantification of % TP cells among total cells within stitched images; mean ± SEM (10.2% ± 2.6%), N = 4 donor tissues. See also Figures S1F and S2.

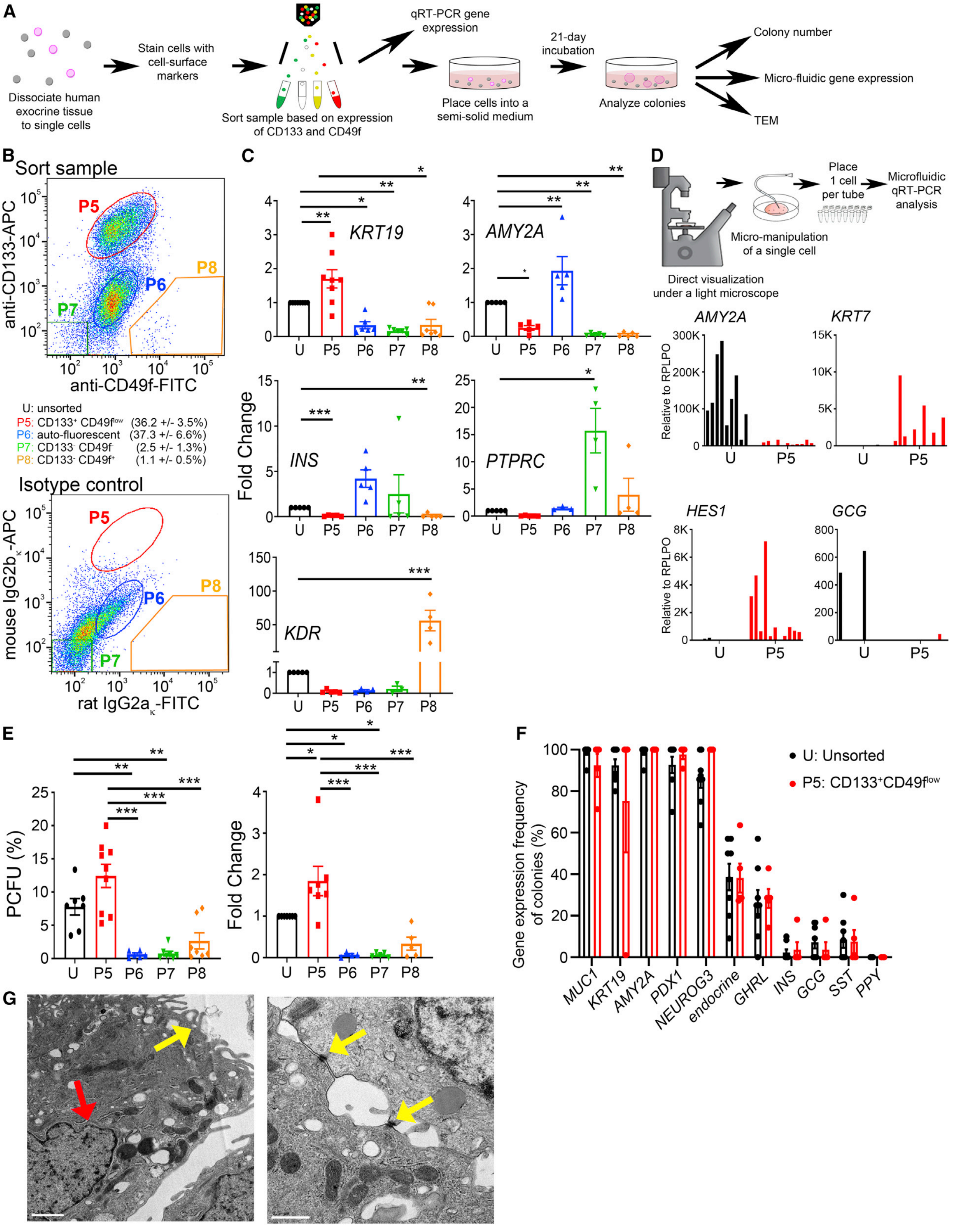

(legend on next page)

Figures S1F andS2B). Some $SOX9^+$/$PDX1^+$ ductal cells lacked NKX6.1 (Figure 2G, red arrows), suggesting heterogeneity. Overall, the TP cells constituted 10.2 ± 2.6% of total pancreatic cells (Figure 2H), a frequency consistent to % PCFUs among dissociated exocrine cells (Figure 1C).

Live-sorted human pancreatic $CD133^+CD49f^{low}$ cells are enriched for ductal cells

CD133 (*PROM1*) is a known ductal marker, and CD49f (*ITGA6*) co-expresses with CD133 in human fetal pancreas (Sugiyama et al., 2007). We therefore tested the utility of these cell surface markers in fluorescence-activated cell sorting (Figure 3A). Freshly dissociated exocrine cells were stained with antibodies against CD133 and CD49f and analyzed using flow cytometry (Figures 3B and S3A). All $CD133^+$ cells expressed low levels of CD49f, indicating that CD49f did not improve adult ductal cell identification, but CD49f separated a $CD133^-$ (non-ductal) subpopulation (see population 8 [P8] below). The $CD133^+CD49f^{low}$ cells (marked as P5) comprised 36.2 ± 3.5% of total dissociated exocrine cells (Figure 3B). We next sorted four subpopulations: $CD133^+CD49f^{low}$ (P5), $CD133^{low}CD49f^{low}$ (P6), $CD133^-CD49f^-$ (P7), and $CD133^-CD49f^+$ (P8). Conventional qRT-PCR analyses revealed that, compared with unsorted cells (U), freshly sorted P5 cells expressed higher levels of the ductal marker *KRT19* and low-to-undetectable levels of endocrine (*INS*) and acinar (*AMY2A*) markers (Figure 3C). Markers for leukocytes (*CD45*) and endothelial cells (*KDR*) were also expressed lower in P5 cells compared with unsorted cells (Figure 3C). Microfluidic qRT-PCR confirmed that freshly sorted, micro-manipulated single P5 cells expressed *KRT7* and low levels of *AMY2A* (Figure 3D). Although most of the islets were already removed, infrequent *GCG* expressing cells remained in the unsorted population (Figure 3D). These results confirm that $CD133^+CD49f^{low}$ cells are specifically enriched for ductal cells.

Sorted human pancreatic $CD133^+CD49f^{low}$ ductal cells are enriched for PCFUs

To assess which pancreatic subpopulation(s) were enriched for PCFUs, sorted cells were plated in our standard colony assay. Unsorted cells from this cohort displayed an overall % PCFU of 7.8 ± 1.3% (Figure 3E, left). Compared with unsorted cells, only P5 displayed a higher (1.9- ± 0.4-fold) % PCFU (Figure 3E, right). The 3-wo colonies grown from P5 cells also appeared as hollow spheres (Figure S3B), with no donor-to-donor variation in colony diameter (Figure S3C). The mean diameter of colonies grown from P5 cells was 316 ± 34 μm (Figure S3D), which is comparable to that of colonies grown from unsorted cells (compare Figure S3D with 1D; $p > 0.05$). Gene expression patterns and frequencies of P5-derived individual colonies were similar to those derived from unsorted cells (Figures 3F and S3E), suggesting that P5-derived PCFUs are tri-potent. Finally, TEM of colonies grown from P5 cells displayed microvilli facing the lumen, nuclei with invaginations, and desmosomes at cell-cell junctions (Figure 3G). Overall, these results demonstrate that sorting does not significantly impact the growth, differentiation, or colony morphology of human PCFUs, and that PCFUs are derived from the ducts. Due to the unchanged colony phenotypes and logistic limitation with cadaveric tissues, unsorted cells were used for subsequent experiments.

Adult human PCFUs self-renew and expand up to 300-fold over 9 weeks in the presence of a ROCK inhibitor

We assessed the self-renewal abilities of PCFUs using serial dissociation of colonies into single cells and re-plating from 1° through 3° cultures (Figure 4A). After 9 weeks, the number of PCFUs only expanded about 3-fold in our standard culture (Figures 4B and 4D). Prior studies found that inhibition of Rho-associated protein kinase (ROCK) enhances the survival of fetal murine pancreatic progenitor cells *in vitro* (Greggio et al., 2013), and that Notch activation is required

Figure 3. Live-sorted human pancreatic $CD133^+CD49f^{low}$ ductal cells are enriched for PCFUs
(A) Experimental diagram.
(B) Representative sorting windows for four cell populations (P5, P6, P7, and P8) and their percentages; mean ± SEM, N = 5 donors.
(C) qRT-PCR analysis of freshly sorted populations, compared with gene expression levels of the unsorted population (U) as fold change for ductal (*KRT19*, N = 6 donors), acinar (*AMY2A*, N = 5 donors), endocrine (*INS*, N = 5), endothelial (*KDR*, N = 3 donors), and leukocyte (*PTPRC*, N = 3 donors) cells; mean ± SEM.
(D) Micro-manipulation of freshly sorted individual cells for microfluidic qRT-PCR analysis. Data are from N = 1 donor with $n \geq 11$ single cells per population.
(E) PCFU among sorted populations compared with unsorted (U) population, expressed as % PCFU (left) or fold change (right) (N = 7 donors; mean ± SEM).
(F) Gene expression frequencies of colonies grown from unsorted (U, black) or sorted (P5, red) cells. U: n = 32 colonies, N = 4 donors. P5: n = 38 colonies, N = 5 donors, mean ± SD. Significance was determined by two-way ANOVA, with Sidak's multiple comparison.
(G) Ultrastructure analysis of P5-derived colonies displaying microvilli on the apical side (left, yellow arrow), nucleus invagination (left, red arrow), and desmosomes (right, yellow arrows). Scale bar, 1 μm (left) or 0.5 μm (right). $^*p < 0.05$, $^{**}p < 0.01$, $^{***}p < 0.001$. See also Figures S3A–S3E.

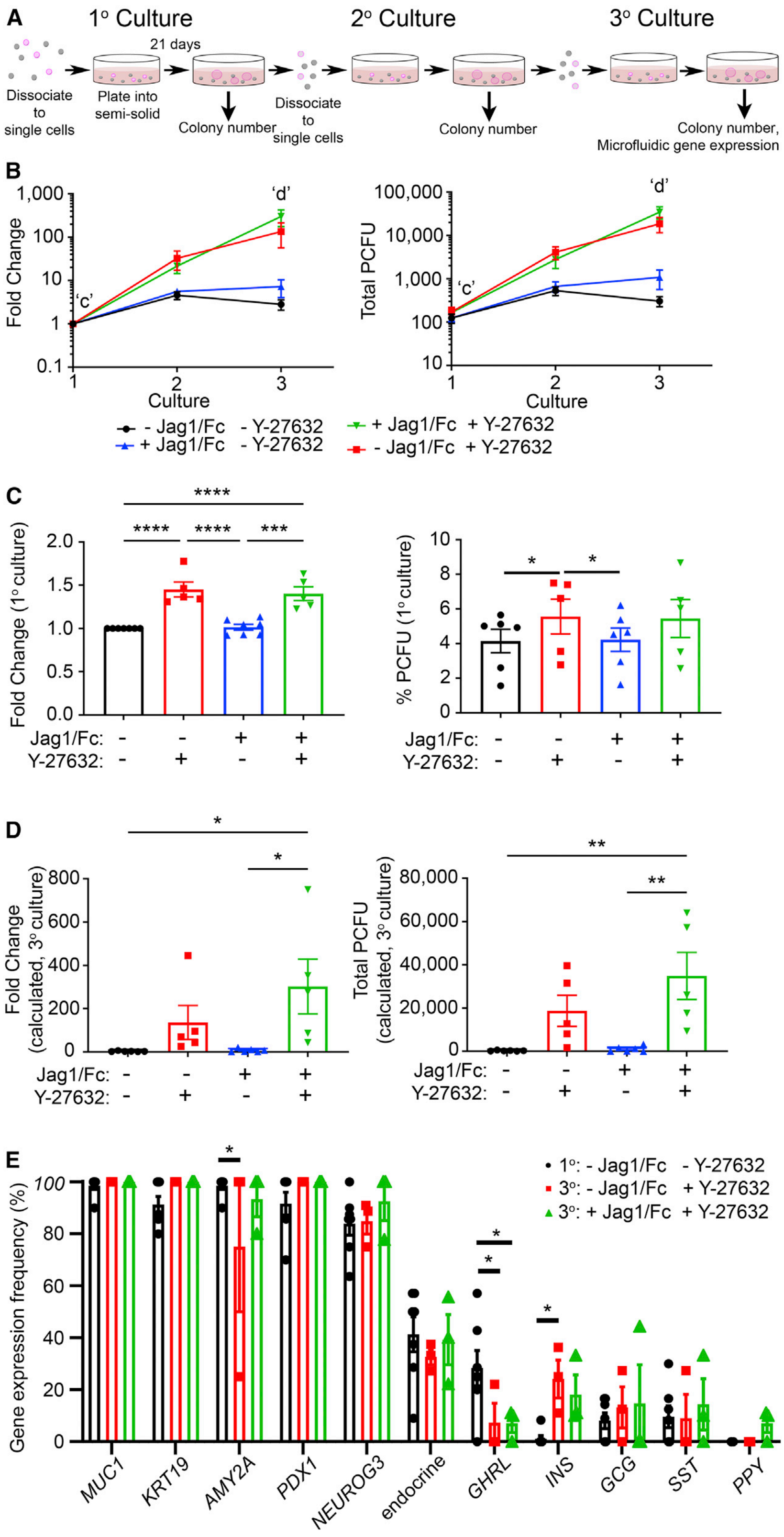

Figure 4. Adult human PCFUs self-renew and expand up to 300-fold

(A) Experimental diagram.

(B) PCFU fold change (left) and total PCFU (right); mean ± SEM, N = 4 donors from 5 (groups containing Y-27632 ± Jag1/Fc) or 6 (control and Jag1/Fc alone) independent experiments, with 4 technical replicates per plating.

(C and D) Further analysis of data from (B); PCFUs in the 1° culture (C) or on the 3° culture (D).

(E) Microfluidic qRT-PCR analysis of n ≥ 8 individually handpicked colonies per donor collected from the 3° culture, grown in the presence of Y-27632 (red; n = 28 total colonies from N = 3 donors) or Y-27632 and Jag1/Fc (green; n = 28 total colonies from N = 3 donors). Black: same data as Figure 1H; mean ± SD. Significance was determined by two-way ANOVA, with Tukey's multiple comparison. *$p < 0.05$, **$p < 0.01$, ***$p < 0.001$, ****$p < 0.0001$. See also Figures S3F–S3H.

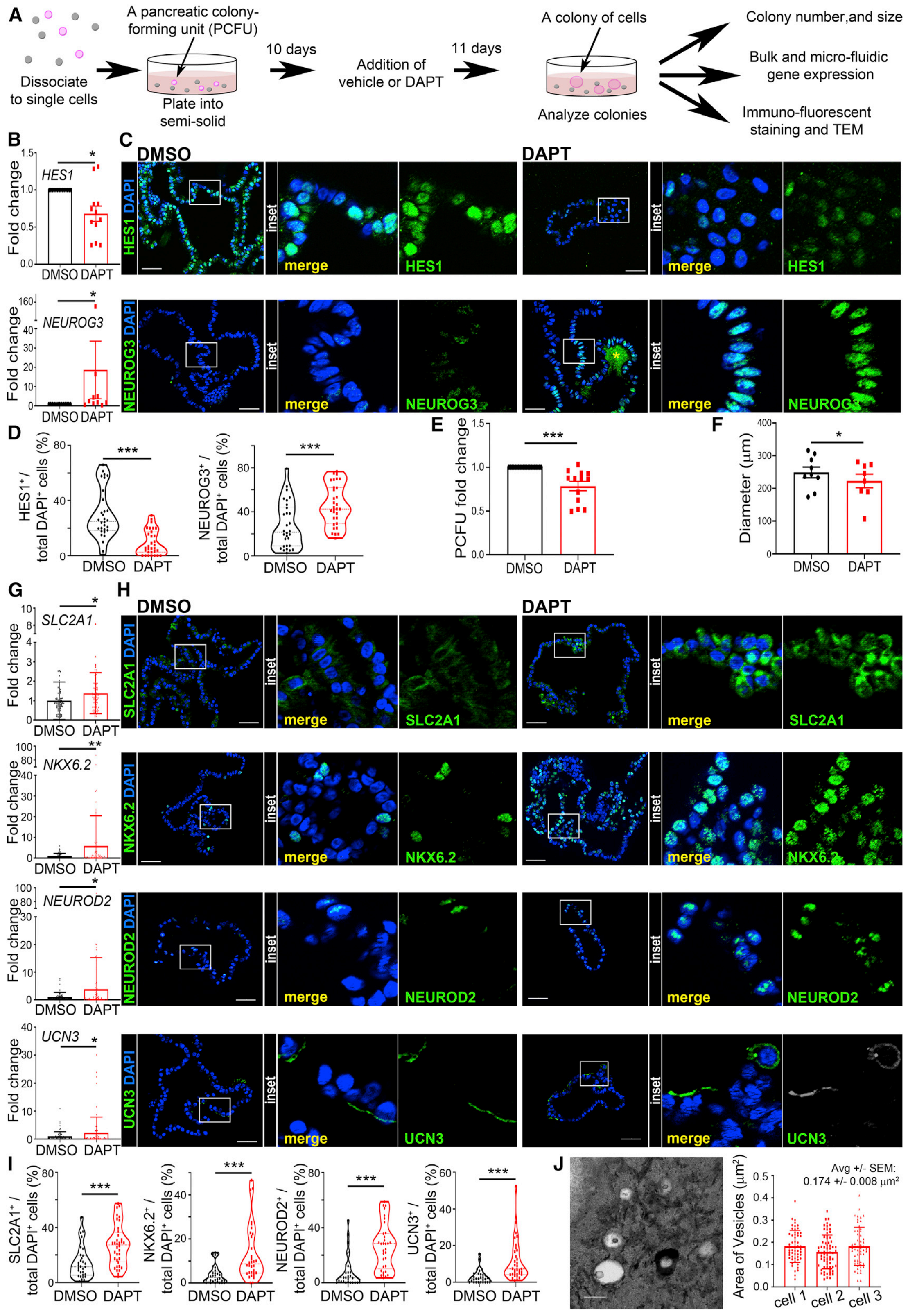

(legend on next page)

for maintaining the progenitor cell pool (Apelqvist et al., 1999). We therefore tested the effects of Y-27632, a ROCK inhibitor, and Jag1/Fc, a Notch activator, on PCFU self-renewal. Compared with the control, addition of Y-27632 with or without Jag1/Fc increased the number of PCFUs over 9 weeks by 302- ± 126-fold or 136- ± 78-fold, respectively (Figure 4B). Y-27632 treatment also increased 1° colonies (Figure 4C), indicating enhanced PCFU survival. In contrast, Jag1/Fc alone did not affect PCFU self-renewal (Figures 4B and 4D) or survival (Figure 4C), even though Jag1/Fc could increase *MKI67* expression in colonies at days 14 and 21 (Figure S3F). In the 3° culture, % PCFUs among total cells plated with Y-27632 was significantly higher than the no addition control (Figure S3G), suggesting PCFU exhaustion over time without ROCK inhibition. These results demonstrate that ROCK inhibition, but not Notch activation, is sufficient for self-renewal of human PCFUs.

To determine whether PCFU tri-potency was preserved after expansion, individual 3° colonies from three donors were micro-manipulated and analyzed using microfluidic qRT-PCR. Only 3° colonies cultured with Y-27632, with or without Jag1/Fc, were analyzed. Similar to 1° colonies (Figure 1H), 3° colonies collectively expressed the three main pancreatic lineage markers (Figures 4E and S3H). There was no difference in the frequency of 3° colonies expressing pancreas lineage markers between colonies grown with Y-27632 and with the combination of Y-27632 + Jag1/Fc (Figure 4E). Interestingly, when comparing the 3° colonies grown with Y-27632 to 1° colonies grown in our standard culture, there was a reduction in the frequency of colonies expressing *AMY2A* and *GHRL*, but an increase of *INS* (Figure 4E). Overall, these data demonstrate that tri-potent PCFUs are preserved over 9 weeks in culture.

Notch inhibition enhances endocrine progenitor gene expression profiles in human colonies

Many micro-manipulated individual $CD133^{+}CD49f^{low}$ cells expressed *HES1* (Figure 3D), a known Notch target gene. The relative low expression of endocrine genes in colonies grown in our standard culture (Figure 1H) prompted us to test Notch inhibition, because the reduction of HES1 can de-repress *NEUROG3* gene expression (Lee et al., 2001; Shih et al., 2012), which is necessary for endocrine lineage commitment (Apelqvist et al., 1999; Jensen et al., 2000; Murtaugh et al., 2003).

DAPT is a small molecule that inhibits gamma-secretase and is known to reduce *HES1* expression levels (Kopinke et al., 2011). It is known that the timing of Notch inhibition is critical for proper endocrine differentiation *in vivo* (Cras-Meneur et al., 2009) and *in vitro* (Shih et al., 2012; Wedeken et al., 2017). Addition of DAPT to human colonies on day 10 (Figure 5A) decreased *HES1* and increased *NEUROG3* expression compared with the vehicle control (Figure 5B). Quantification of IF staining confirmed a reduction of $HES1^{+}$ cells and an increase of $NEUROG3^{+}$ cells in DAPT-treated colonies (Figures 5C, 5D, and S4A). DAPT treatment reduced % PCFU (Figure 5E) and colony size (Figure 5F), suggesting that Notch signaling is necessary for the survival and growth of PCFUs, similar to behavior from pancreatic progenitors (Apelqvist et al., 1999).

Microfluidic qRT-PCR analysis of individual colonies revealed that DAPT did not increase the frequency of tri-lineage colonies (Figure S4B) but increased the expression of several beta cell maturation markers, including *SLC2A1*, *NKX6.2*, *NEUROD2*, and *UCN3* (Figure 5G). IF staining and quantification confirmed that an increased proportion of cells within DAPT-treated colonies express these maturation markers (Figures 5H, 5I, and S4C). These results

Figure 5. Notch inhibition enhances endocrine gene expression in human colonies
(A) Experimental diagram.
(B) Colonies analyzed for *HES1* and *NEUROG3* gene expression by qRT-PCR; mean ± SEM, n = 13 experiments, N = 9 donors.
(C) IF of colonies treated with DMSO (left) or DAPT (right) with HES1 (green, top) or NEUROG3 (green, bottom) and DAPI (blue). Yellow star (*, bottom-right) denotes an area of non-specific staining. Scale bar, 50 μm (box enlarged 4× to the right).
(D) IF quantification of percent positive cells. n = 30–31 colonies treated with DMSO or DAPT from N = 3 donors. Each dot represents a colony.
(E) % PCFU (fold change); mean ± SEM, N = 13 donors.
(F) Average colony diameter; mean ± SEM, N = 8 donors.
(G) Fold change of gene expression from individual colonies examined by microfluidic qRT-PCR analysis; mean ± SD, n = 73–78 colonies treated with DMSO or DAPT, N = 5 donors. Each dot represents a colony.
(H) IF analysis of endocrine markers in DMSO-treated colonies (left) or DAPT (right). Scale bar, 50 μm (insets enlarged 4× to the right).
(I) IF quantification of percent positive cells; mean ± SD, n = 30 DMSO and n = 34 DAPT, N = 3 donors. Each dot represents a colony.
(J) DAPT-treated colonies were analyzed using TEM and three-dimensional scanning electron microscopy (3D-SEM). Left: a representative TEM photomicrograph of a portion of a cell showing vesicles with granules. Scale bar, 500 nm. Right: the area of the vesicles containing insulin-like granules were measured in 3 cells. Data represent areas from 55, 67, and 56 individual vesicles from cells 1, 2, and 3, respectively. *$p < 0.05$, **$p < 0.01$, ***$p < 0.001$. See also Figure S4.

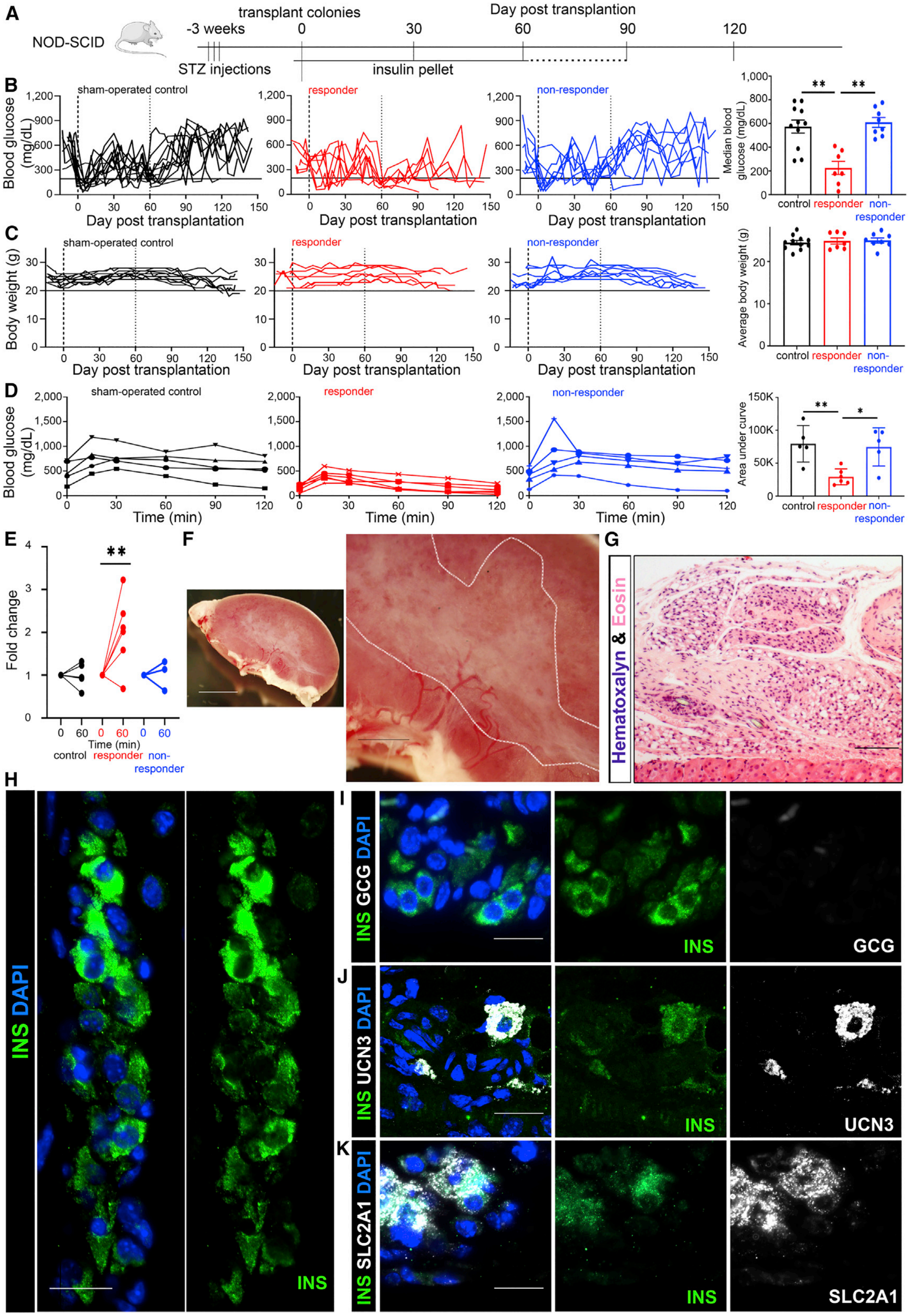

(legend on next page)

demonstrate that Notch inhibition directs differentiation toward an endocrine phenotype in our colonies but does not change a PCFU from bi- to tri-potent.

To clarify if DAPT induced the formation of insulin vesicles, we performed TEM analysis of DAPT-treated colonies and found condensed insulin granules in vesicles (Figure 5J), which were not observed in control colonies. Using 3D-SEM, we analyzed 178 non-overlapping insulin vesicles from 3 cells and found that the mean area was 0.17 ± 0.01 μm^2/vesicle (Figure 5J). The vesicles in DAPT-treated cells were slightly smaller than reported endogenous insulin vesicles (0.19 μm^2/vesicle) (Fava et al., 2012). Also, the insulin granules appeared faint compared with adult beta cells, suggesting functional immaturity (Ni et al., 2017).

DAPT-treated human colonies give rise to insulin-expressing cells in hyperglycemic mice

We determined whether DAPT-treated human colonies may further differentiate and become functionally mature in insulin-dependent diabetic NOD-SCID mice (Figure 6A). Streptozotocin was injected to mice to destroy beta cells and induce hyperglycemia (fasting blood glucose >200 mg/dL). Subsequently, 3-wo DAPT-treated colonies were pooled and placed under the kidney capsule at 1–2.5 million cells per mouse. An insulin pellet was inserted when fasting blood glucose exceeded 450 mg/dL to minimize the detrimental effects of overt hyperglycemia (Brereton et al., 2016). To test graft function without interference, insulin pellets were not inserted 60 days post-transplantation. Colonies from 4 different donor tissues were independently transplanted into multiple mice (n ≥ 2 mice per donor tissue, 15 mice total).

Between days 90 and 120 post-transplantation, body weight was similar (Figure S5A) while median fasting blood glucose trended lower but did not reach significance in diabetic mice transplanted with colonies compared with sham controls (Figure S5B; individual mouse data in Figure S5C). However, a difference was found when we ranked transplant recipients based on their median fasting blood glucose, separating the top 8 from the bottom 7 mice—the bottom 7 mice (herein "responders") displayed a median fasting blood glucose at ~200 mg/dL (Figure 6B). Control diabetic mice did not approach a median fasting blood glucose of 200 mg/dL (Figure 6B), suggesting a lack of beta cells; this was confirmed using H&E staining (Figure S5D). Again, body weights were not different between responders and non-responders (Figure 6C). Importantly, colonies from each donor tissue transplanted into diabetic mice resulted in at least one responder mouse (Figure S5E); this suggests that 1–2.5 million DAPT-treated cells per mouse are the marginal mass, the minimum number of cells necessary to reverse hyperglycemia in some but not all transplanted diabetic mice.

To further analyze graft function, we performed an intraperitoneal glucose tolerance test (IP-GTT), which revealed a trend of better glucose clearance in transplanted mice (Figure S5F), with a significant decrease in mean area under the curve (AUC) in the responder mice (Figure 6D). Human C-peptide levels in blood 1 h after glucose stimulation significantly increased in the responder mice (Figure 6E). However, stimulated human C-peptide concentration was 2.8 ± 1.9 pmol/L, which was much lower than the 366 ± 154 pmol/L of human C-peptide detected in hyperglycemic NOD-SCID mice transplanted with 1,200 human islets (Table S1) 90–150 days post-transplantation (Figure S5G). This result prompted us to calculate the ratio of blood glucose to human C-peptide in individual mice; a lower ratio indicates a better function of the graft (Takita and Matusmoto, 2012). The colony transplanted mice showed higher ratio than the mice with human islet grafts (Figure S5H), suggesting functional immaturity of our grafted colonies *in vivo* compared with adult islets.

To detect beta-like cells in the transplant mice, we dissected the kidney grafts (Figures 6F and 6G) 3–4 months post-transplantation for IF analysis. INS^+ cell clusters that did not co-express GCG were found (Figures 6H and 6I), suggesting the presence of mono-hormonal beta-like cells (Herrera, 2000). Some INS^+ cells also co-expressed beta cell maturation markers UCN3 or SLC2A1 (Figures 6J, 6K, S6A, and S6B). Together, these results show that colonies

Figure 6. DAPT-treated human colonies give rise to insulin-expressing cells in insulin-dependent diabetic mice

(A–C) (A) Experimental diagram. Data were analyzed between day 90 and 120 post-transplantation for (B) blood glucose and (C) body weight, with individual mice separated by sham-operated control (black, n = 11), responder (red, n = 7) and non-responder (blue, n = 8) mice. Data represent median blood glucose or average body weight ± SEM.

(D) IP-GTT analysis on control (black, n = 5), responder (red, n = 6), and non-responder (blue, n = 5) mice. AUC was analyzed as mean ± SD.

(E) Human C-peptide in serum, expressed as relative C-peptide fold change between time 0 and 60 min post glucose challenge from control (black, n = 4), responder (red, n = 6), and non-responder (blue, n = 3) mice.

(F) Bright-field image of a kidney grafted with DAPT-treated human colonies. Grafted cells are outlined (white dashed line). Scale bars, 3 mm (left) or 1 mm (right).

(G) H&E staining of a kidney graft, with kidney tissue shown at the bottom of the image. Scale bar, 200 μm.

(H–K) IF staining of grafted cells with INS (green) (G and H), GCG (white) (H), UCN3 (white) (J), or SLC2A1 (white) (K). Scale bar, 20 μm. $^*p < 0.05$, $^{**}p < 0.01$. See also Figures S5 and S6.

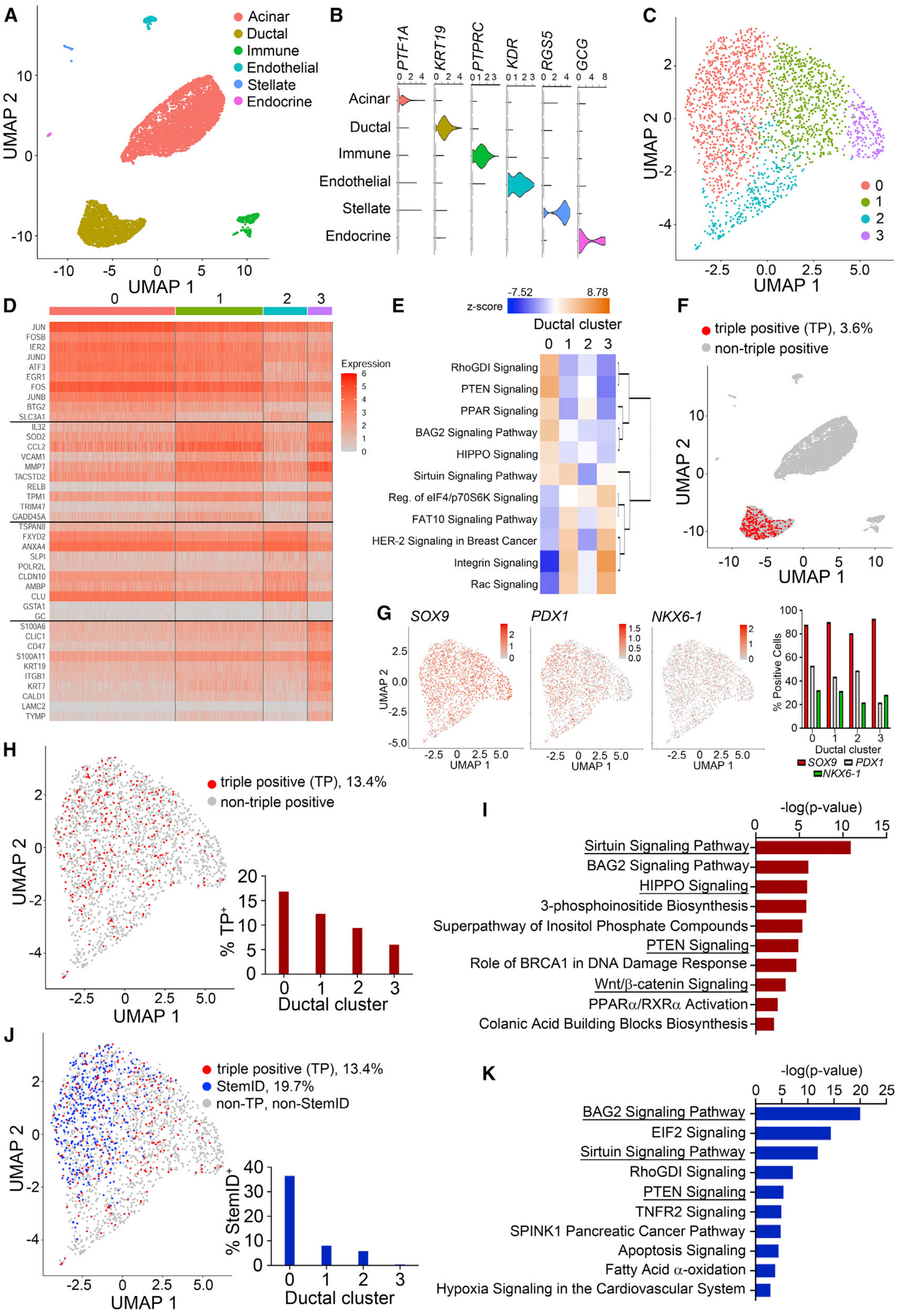

(legend on next page)

pre-treated with DAPT can differentiate into beta-like cells after transplantation into diabetic mice.

scRNA-seq analysis reveals a subset of ductal cells as progenitor-like cells

To gain insight into gene expression patterns of human pancreatic ducts, we performed scRNA-seq analysis using freshly dissociated exocrine tissue. An estimated 14,822 cells were read at 103,333 mean reads per cell. Events that passed quality control (7,812 cells, Figures S7A and S7B) were subsequently analyzed using the Seurat R package. Using Uniform Manifold Approximation and Projection dimensional reduction, we identified clusters of acinar (*PTF1A*: 5,094 cells), ductal (*KRT19*: 2,119 cells), immune (*PTPRC*: 326 cells), endothelial (*KDR*: 185 cells), stellate (*RGS5*: 50 cells), and endocrine cells (*GCG*: 38 cells) (Figures 7A and 7B). Consistent with the sorting results (Figure 3), the ductal cell cluster expressed *PROM1* (CD133) with minimal expression of *ITGA6* (CD49f) (Figure S7C).

To identify potential progenitor cell population within ductal cells, we performed unbiased principal-component analysis of the ductal cluster and found 4 distinct subpopulations (Figure 7C); the top 10 cluster-specific genes are presented in a heatmap (Figure 7D; differentially expressed [DE] genes in Data S1). Cluster 0 was identified with progenitor genes such as *JUN* and *FOS* (Goncalves et al., 2021), whereas cluster 3 showed more mature ductal genes such as *KRT19* and *KRT7*. Bioinformatics analysis using Ingenuity Pathway Analysis (IPA) software revealed the top 6 predicted upregulated and top 5 downregulated canonical pathways for cluster 0, which showed both similar and divergent pathways against other ductal clusters (Figure 7E). These results support the heterogeneity hypothesis on adult human pancreatic ductal cells.

TP cells were exclusively found in the ductal cluster (Figure 7F), consistent with IF analysis in ducts (Figures 2G and 2H). Independent assessment of *SOX9*, *PDX1*, or *NKX6.1* expression in total cells (Figure S7D) and within the four ductal clusters revealed that *PDX1* and *NKX6.1* were the limiting factors (Figure 7G). Overall, TP cells were 3.6% of total cells (Figure 7F) and 13.4% of ductal cells (Figure 7H), with a majority found in cluster 0 (Figure 7H); these percentages were within range of colony-forming efficiency of unsorted and sorted CD133^{+}CD49^{flow} ductal (P5) cells (Figure 3E, left). DE genes between the TP cells and other non-TP ductal cells (Data S1) were analyzed by IPA, which revealed upregulated Sirtuin, HIPPO, PTEN, and Wnt-β catenin pathways in TP cells (Figure 7I). These pathways are known to be involved in pancreas development.

The StemID algorithm (Grun et al., 2016) was applied to independently re-cluster all of the ductal cells, and each new cluster according to their stem potential was scored. We selected the cluster with the highest score, StemID cluster 4 (Figures S7E–S7I), and re-mapped those cells to the original clustering analysis. StemID cluster 4 was mapped to 19.7% of the total ductal cells and 36.5% of cluster 0 ductal cells (Figure 7J). DE gene expression of StemID cluster 4 compared with other ductal cells (Data S1) was analyzed in IPA. Predicted upregulated pathways, such as BAG2, Sirtuin, and PTEN signaling, were identified in the StemID cells (Figure 7K); they were also found in the TP cell analysis, indicating similarities between the two populations. Together, these data provide evidence that a subset of ductal cells have progenitor properties.

DISCUSSION

In this study, we show evidence for the existence of self-renewing progenitor-like cells from the adult human pancreatic ducts, which we call PCFUs. By using single-cell micro-manipulation, we provide a rigorous demonstration of multi-lineage differentiation potential of PCFUs *in vitro*. In addition, we show for the first time that colonies and adult human ducts *in vivo* contain cells capable of expressing embryonic MPC markers (SOX9^{+}/PDX1^{+}/NKX6.1^{+}), which we call TP cells.

Figure 7. Human ducts are heterogeneous with a subset resembling progenitor-like cells

(A) scRNA-seq of dissociated adult human exocrine tissue identifies 6 distinct clusters.

(B) Violin plots of representative gene markers.

(C) The ductal cluster was re-analyzed by principal-component analysis and segregated into four unique clusters (0–3).

(D) Heatmap of cluster-specific genes identified in the four ductal clusters.

(E) IPA predicted up and downregulated pathways using DE genes from the four ductal clusters.

(F) Cells simultaneously expressing *SOX9*, *PDX1*, and *NKX6.1* are 3.6% of the total cells, and only in the ductal cell population.

(G) Uniform Manifold Approximation and Projections (UMAPs) of the ductal cluster showing the expression of the *SOX9*, *PDX1*, or *NKX6.1*, with percent positive cells calculated in each ductal cluster.

(H) TP cells are enriched in ductal cluster 0.

(I) IPA-predicted upregulated pathways in TP cells.

(J) StemID cluster 4 cells (blue) are overlaid with TP cells (red) within the ductal cluster.

(K) IPA-predicted upregulated pathways in StemID cluster 4. See also Figure S7 and Data S1.

Tremendous progress has been made in 3D organoid technology. Many current organoid culture techniques are modeled after a study that established epithelial organoid culture using Matrigel (Sato et al., 2011), where high concentrations (>90% v/v) of Matrigel are used to embed and immobilize cells. However, it is difficult to micromanipulate individual organoids; one reason is the elevated viscosity and rigidity caused by the high Matrigel concentrations. Our 3D colony assay differs in that viscous methylcellulose is added (Perko et al., 2011), allowing the dilution of Matrigel to a much lower concentration (5% v/v in this study) and aiding micromanipulation.

Using organoid culture systems based on the intestinal organoid platform from Sato et al., several studies show that pancreatic ductal cells can expand *in vitro*, but those ductal cells possess only two lineage potential (duct and endocrine) or with limited self-renewal. Loomans et al. (2018) found that the net expansion of total ductal cells was approximately 20-fold over 20 weeks. Lee et al. (2013) observed an 8-fold increase of total cells over 6–9 weeks, and Georgakopoulos et al. (2020) showed an impressive expansion of organoids for over 15 weeks. In contrast, rather than mechanical digestion into cell clumps as in the other studies, we expanded our colonies by enzymatic digestion to single cells during passaging, observing up to 300-fold expansion of PCFUs over 9 weeks (Figure 4). In addition, PCFUs comprise ~8% of the total cells in the 3° culture (Figure S3G); therefore, total expansion of our ductal cells is calculated to be about 3,750-fold. We also report that colonies after expansion maintain the same gene expression patterns as colonies from the first culture, showing preservation of tri-lineage potency (Figure 4E). Thus, PCFUs may represent the true self-renewing progenitor cells from the adult human pancreas.

With respect to lineage potential, Loomans et al. (2018) showed that progenitor-like cells marked by ALDH^{high} staining possess duct and endocrine lineage potential. In contrast, our PCFUs possess duct, endocrine, and acinar lineage potential. Lee et al. (2013) reported that their ductal cells cannot be transdifferentiated into INS$^+$ cells in organoid culture unless forced to express NGN3, MAFA, and PDX1. Interestingly, Qadir et al. (2018, 2020) demonstrated that sorted adult human P2RY1$^+$/ALK3^{bright} cells, which are found in the main ducts, can give rise to the three major pancreatic lineages using a 2D attachment culture system. Our data agree with their findings that some ductal cells possess tri-lineage potential. However, Qadir et al. did not report the self-renewal capacity of the P2RY1$^+$/ALK3^{bright} cells, which is an important aspect of progenitor cells.

Cellular compartments in the adult pancreas had been largely considered homogeneous, but increasing evidence suggests that endocrine (Baron et al., 2016; Butler et al., 2010), acinar (Kusmartseva et al., 2020), and ductal cells (Baron et al., 2016; Grun et al., 2016; Qadir et al., 2020) are heterogeneous. In this study, we not only confirm ductal cell heterogeneity among adult human exocrine tissue (Figures 2G and 7), but we also add functional heterogeneity in colony formation among sorted human ductal cells (Figure 3E). This ductal cell heterogeneity may explain the difficulties of Cre-lox lineage tracing using a pan-duct marker, such as *Sox9* (Kopp et al., 2011) or *Hnf1b* (Solar et al., 2009), to detect significant activities of adult murine pancreatic progenitor cells due to the relatively minor population of progenitor cells. In addition, subtle differences in the expression levels of progenitor cell markers may dictate functionality (Rezanejad et al., 2018). Thus, to address ductal cell heterogeneity further, future experiments are needed to identify unique cell surface markers in combination with CD133, but not CD49f, that enrich or purify the ductal progenitor cells.

A potential clinically relevant finding of our study is that DAPT-treated colonies grafted into diabetic mice give rise to beta-like cells *in vivo*. Although, our grafts did not result in high levels of human C-peptide, our transplant mice did show an observable drop in blood glucose levels (i.e., in responder mice) between 3 and 4 months post-transplantation (Figure 6B). These results raise the possibility for proinsulin, rather than C-peptide or insulin, as the predominant form of the insulin gene product that is secreted from our colony grafts—a possibility that requires future investigation. Proinsulin has been reported to exert biological effects in development and various adult cell types (Malaguarnera et al., 2012) and therefore may provide clinically beneficial effects to the hyperglycemic mice. Loomans et al. (2018) transplanted ductal organoids under the kidney capsule of mice (up to 4.5×10^5 cells per mouse) and detected INS$^+$KRT19$^-$ cells. However, their mice were followed for only 1 month post-transplantation; it remains unknown whether their INS$^+$ cells improve glucose regulation over a longer period. Pluripotent stem cell (PSC)-derived insulin-expressing cells have been shown to regulate blood glucose levels in insulin-dependent diabetic mice after transplantation (Migliorini et al., 2021). However, there is the concern of teratoma formation from undifferentiated PSCs (Cunningham et al., 2012). In contrast to PSCs, adult stem cells do not give rise to teratomas. Thus, should PSC-derived products raise safety concerns in future clinical trials, adult PCFUs can be a suitable alternative source of insulin-expressing cells.

In summary, we have shown in functional *in vitro* assays that some adult human ductal cells, resembling progenitor cells, are capable of tri-lineage differentiation and self-renewal in a unique 3D methylcellulose-containing culture system. Also, we identified a subset of human

pancreatic ductal cells capable of expressing TP progenitor markers through IF and *in silico* analysis. Given the severe shortage of donor organs, our results suggest a potential utility of human cadaveric ductal tissues for therapy in insulin-dependent diabetic patients.

EXPERIMENTAL PROCEDURES

Resource availability

Corresponding author

The data that support the findings of this study are available from the corresponding author, Janine C. Quijano (jquijano@coh.org), upon reasonable request.

Materials availability

This study did not generate new unique reagents.

Single-cell suspension

Donated pancreata were procured and shipped to City of Hope for isolation of islets (Qi et al., 2015). All tissues used in this study had consent for research from close relatives of the donors. After islet removal, de-identified human pancreata were obtained from the Southern California Islet Cell Resource (SC-ICR) Center at the City of Hope. The exocrine tissue was dissociated to yield a single-cell suspension before cryopreservation, culture, or other procedures.

Mice

Mice used in this study were maintained according to protocols approved by the City of Hope Institutional Animal Care and Use Committee.

Additional detailed experimental methods are provided in the supplemental information.

Data availability

The data that support the findings of this study are available from the corresponding author upon reasonable request. Single-cell RNA sequencing (scRNA-seq) data are available from Gene Expression Omnibus (GEO) database: GSE153834.

SUPPLEMENTAL INFORMATION

Supplemental information can be found online at https://doi.org/10.1016/j.stemcr.2023.02.001.

AUTHOR CONTRIBUTIONS

Conceptualization, J.C.Q., L.W., and H.T.K.; methodology, J.C.Q., L.W., and H.T.K.; software, M.-H.C.; formal analysis, J.C.Q. and L.W.; investigation, J.C.Q., L.W., J.A.O., J.M.L., A.L., J.R., J.M.M., K.L., H.N.Z., J.R.T., K.J., and C.M.-D.; resources, I.H.A. and F.K.; writing – original draft, J.C.Q., L.W., and H.T.K.; writing – review & editing, J.C.Q., L.W., J.A.O., H.N.Z., J.R.T., I.H.A., D.C.T., F.K., A.D.R., and H.T.K.; supervision, J.C.Q., L.W., and H.T.K.; funding acquisition, J.C.Q., L.W., and H.T.K.

ACKNOWLEDGMENTS

We thank Christiana J. Crook for technical writing and editing, and Elena C. Chen for graphic illustration. The graphical abstract was created using BioRender.com. We also thank services provided by the following research cores at the City of Hope: Pathology, Analytical Flow Cytometry, Light Microscopy Digital Imaging, Animal Resource Center, Integrative Genomics, and Electron Microscopy. This work is supported, in part, by postdoctoral fellowships to J.C.Q. from JDRF, New York (3-PDF-2016-174-A-N) and to L.W. from the California Institute for Regenerative Medicine (CIRM) (TG2-01150), and by grants from the National Institutes of Health to H.T.K. (R01DK099734 and R56DK099734), the City of Hope Research Facilities (P30CA33572), and the EM core (P30CA0668). Support from the Wanek Family Project for Type 1 Diabetes to H.T.K. is also gratefully acknowledged.

CONFLICT OF INTERESTS

H.T.K. maintains a patent no. 9,783,784 titled “Methods for establishing and improving the survival of a population of pancreatic progenitor or stem cells.” The other authors declare no competing interests.

Received: February 21, 2022
Revised: January 31, 2023
Accepted: February 1, 2023
Published: March 2, 2023

REFERENCES

Al-Hasani, K., Pfeifer, A., Courtney, M., Ben-Othman, N., Gjernes, E., Vieira, A., Druelle, N., Avolio, F., Ravassard, P., Leuckx, G., et al. (2013). Adult duct-lining cells can reprogram into beta-like cells able to counter repeated cycles of toxin-induced diabetes. Dev. Cell *26*, 86–100. https://doi.org/10.1016/j.devcel.2013.05.018.

Apelqvist, A., Li, H., Sommer, L., Beatus, P., Anderson, D.J., Honjo, T., Hrabe de Angelis, M., Lendahl, U., and Edlund, H. (1999). Notch signalling controls pancreatic cell differentiation. Nature *400*, 877–881. https://doi.org/10.1038/23716.

Baron, M., Veres, A., Wolock, S.L., Faust, A.L., Gaujoux, R., Vetere, A., Ryu, J.H., Wagner, B.K., Shen-Orr, S.S., Klein, A.M., et al. (2016). A single-cell transcriptomic map of the human and mouse pancreas reveals inter- and intra-cell population structure. Cell Syst. *3*, 346–360.e4. https://doi.org/10.1016/j.cels.2016.08.011.

Bartfeld, S., Bayram, T., van de Wetering, M., Huch, M., Begthel, H., Kujala, P., Vries, R., Peters, P.J., and Clevers, H. (2015). In vitro expansion of human gastric epithelial stem cells and their responses to bacterial infection. Gastroenterology *148*, 126–136.e6. https://doi.org/10.1053/j.gastro.2014.09.042.

Boj, S.F., Hwang, C.I., Baker, L.A., Chio, I.I.C., Engle, D.D., Corbo, V., Jager, M., Ponz-Sarvise, M., Tiriac, H., Spector, M.S., et al. (2015). Organoid models of human and mouse ductal pancreatic cancer. Cell *160*, 324–338. https://doi.org/10.1016/j.cell.2014.12.021.

Bonner-Weir, S., Taneja, M., Weir, G.C., Tatarkiewicz, K., Song, K.H., Sharma, A., and O’Neil, J.J. (2000). In vitro cultivation of human islets from expanded ductal tissue. Proc. Natl. Acad. Sci. USA *97*, 7999–8004.

Brereton, M.F., Rohm, M., Shimomura, K., Holland, C., Tornovsky-Babeay, S., Dadon, D., Iberl, M., Chibalina, M.V., Lee, S., Glaser, B., et al. (2016). Hyperglycaemia induces metabolic dysfunction and

glycogen accumulation in pancreatic beta-cells. Nat. Commun. *7*, 13496. https://doi.org/10.1038/ncomms13496.

Butler, A.E., Galasso, R., Matveyenko, A., Rizza, R.A., Dry, S., and Butler, P.C. (2010). Pancreatic duct replication is increased with obesity and type 2 diabetes in humans. Diabetologia *53*, 21–26. https://doi.org/10.1007/s00125-009-1556-8.

Cras-Méneur, C., Li, L., Kopan, R., and Permutt, M.A. (2009). Presenilins, notch dose control the fate of pancreatic endocrine progenitors during a narrow developmental window. Genes Dev. *23*, 2088–2101. https://doi.org/10.1101/gad.1800209.

Cunningham, J.J., Ulbright, T.M., Pera, M.F., and Looijenga, L.H.J. (2012). Lessons from human teratomas to guide development of safe stem cell therapies. Nat. Biotechnol. *30*, 849–857. https://doi.org/10.1038/nbt.2329.

Dirice, E., De Jesus, D.F., Kahraman, S., Basile, G., Ng, R.W., El Ouaamari, A., Teo, A.K.K., Bhatt, S., Hu, J., and Kulkarni, R.N. (2019). Human duct cells contribute to beta cell compensation in insulin resistance. JCI Insight *4*, e99576. https://doi.org/10.1172/jci.insight.99576.

Dorrell, C., Tarlow, B., Wang, Y., Canaday, P.S., Haft, A., Schug, J., Streeter, P.R., Finegold, M.J., Shenje, L.T., Kaestner, K.H., and Grompe, M. (2014). The organoid-initiating cells in mouse pancreas and liver are phenotypically and functionally similar. Stem Cell Res. *13*, 275–283. https://doi.org/10.1016/j.scr.2014.07.006.

Fava, E., Dehghany, J., Ouwendijk, J., Müller, A., Niederlein, A., Verkade, P., Meyer-Hermann, M., and Solimena, M. (2012). Novel standards in the measurement of rat insulin granules combining electron microscopy, high-content image analysis and in silico modelling. Diabetologia *55*, 1013–1023. https://doi.org/10.1007/s00125-011-2438-4.

Georgakopoulos, N., Prior, N., Angres, B., Mastrogiovanni, G., Cagan, A., Harrison, D., Hindley, C.J., Arnes-Benito, R., Liau, S.S., Curd, A., et al. (2020). Long-term expansion, genomic stability and in vivo safety of adult human pancreas organoids. BMC Dev. Biol. *20*, 4. https://doi.org/10.1186/s12861-020-0209-5.

Gonçalves, C.A., Larsen, M., Jung, S., Stratmann, J., Nakamura, A., Leuschner, M., Hersemann, L., Keshara, R., Perlman, S., Lundvall, L., et al. (2021). A 3D system to model human pancreas development and its reference single-cell transcriptome atlas identify signaling pathways required for progenitor expansion. Nat. Commun. *12*, 3144. https://doi.org/10.1038/s41467-021-23295-6.

Greggio, C., De Franceschi, F., Figueiredo-Larsen, M., Gobaa, S., Ranga, A., Semb, H., Lutolf, M., and Grapin-Botton, A. (2013). Artificial three-dimensional niches deconstruct pancreas development in vitro. Development *140*, 4452–4462. https://doi.org/10.1242/dev.096628.

Gribben, C., Lambert, C., Messal, H.A., Hubber, E.L., Rackham, C., Evans, I., Heimberg, H., Jones, P., Sancho, R., and Behrens, A. (2021). Ductal Ngn3-expressing progenitors contribute to adult beta cell neogenesis in the pancreas. Cell Stem Cell *28*, 2000–2008.e4. https://doi.org/10.1016/j.stem.2021.08.003.

Grün, D., Muraro, M.J., Boisset, J.C., Wiebrands, K., Lyubimova, A., Dharmadhikari, G., van den Born, M., van Es, J., Jansen, E., Clevers, H., et al. (2016). De novo prediction of stem cell identity using single-cell transcriptome data. Cell Stem Cell *19*, 266–277. https://doi.org/10.1016/j.stem.2016.05.010.

Gu, G., Dubauskaite, J., and Melton, D.A. (2002). Direct evidence for the pancreatic lineage: NGN3+ cells are islet progenitors and are distinct from duct progenitors. Development *129*, 2447–2457.

Herrera, P.L. (2000). Adult insulin- and glucagon-producing cells differentiate from two independent cell lineages. Development *127*, 2317–2322.

Huch, M., Bonfanti, P., Boj, S.F., Sato, T., Loomans, C.J.M., van de Wetering, M., Sojoodi, M., Li, V.S.W., Schuijers, J., Gracanin, A., et al. (2013). Unlimited in vitro expansion of adult bi-potent pancreas progenitors through the Lgr5/R-spondin axis. EMBO J. *32*, 2708–2721. https://doi.org/10.1038/emboj.2013.204.

Inada, A., Nienaber, C., Katsuta, H., Fujitani, Y., Levine, J., Morita, R., Sharma, A., and Bonner-Weir, S. (2008). Carbonic anhydrase II-positive pancreatic cells are progenitors for both endocrine and exocrine pancreas after birth. Proc. Natl. Acad. Sci. USA *105*, 19915–19919. https://doi.org/10.1073/pnas.0805803105.

Jennings, R.E., Berry, A.A., Kirkwood-Wilson, R., Roberts, N.A., Hearn, T., Salisbury, R.J., Blaylock, J., Piper Hanley, K., and Hanley, N.A. (2013). Development of the human pancreas from foregut to endocrine commitment. Diabetes *62*, 3514–3522. https://doi.org/10.2337/db12-1479.

Jensen, J., Heller, R.S., Funder-Nielsen, T., Pedersen, E.E., Lindsell, C., Weinmaster, G., Madsen, O.D., and Serup, P. (2000). Independent development of pancreatic alpha- and beta-cells from neurogenin3-expressing precursors: a role for the notch pathway in repression of premature differentiation. Diabetes *49*, 163–176.

Jin, L., Feng, T., Shih, H.P., Zerda, R., Luo, A., Hsu, J., Mahdavi, A., Sander, M., Tirrell, D.A., Riggs, A.D., and Ku, H.T. (2013). Colony-forming cells in the adult mouse pancreas are expandable in Matrigel and form endocrine/acinar colonies in laminin hydrogel. Proc. Natl. Acad. Sci. USA *110*, 3907–3912. https://doi.org/10.1073/pnas.1301889110.

Kopinke, D., Brailsford, M., Shea, J.E., Leavitt, R., Scaife, C.L., and Murtaugh, L.C. (2011). Lineage tracing reveals the dynamic contribution of Hes1+ cells to the developing and adult pancreas. Development *138*, 431–441. https://doi.org/10.1242/dev.053843.

Kopp, J.L., Dubois, C.L., Schaffer, A.E., Hao, E., Shih, H.P., Seymour, P.A., Ma, J., and Sander, M. (2011). Sox9+ ductal cells are multipotent progenitors throughout development but do not produce new endocrine cells in the normal or injured adult pancreas. Development *138*, 653–665. https://doi.org/10.1242/dev.056499.

Kusmartseva, I., Beery, M., Hiller, H., Padilla, M., Selman, S., Posgai, A., Nick, H.S., Campbell-Thompson, M., Schatz, D.A., Haller, M.J., et al. (2020). Temporal analysis of amylase expression in control, autoantibody-positive, and type 1 diabetes pancreatic tissues. Diabetes *69*, 60–66. https://doi.org/10.2337/db19-0554.

Lee, J., Sugiyama, T., Liu, Y., Wang, J., Gu, X., Lei, J., Markmann, J.F., Miyazaki, S., Miyazaki, J.I., Szot, G.L., et al. (2013). Expansion and conversion of human pancreatic ductal cells into insulin-secreting endocrine cells. Elife *2*, e00940. https://doi.org/10.7554/eLife.00940.

Lee, J.C., Smith, S.B., Watada, H., Lin, J., Scheel, D., Wang, J., Mirmira, R.G., and German, M.S. (2001). Regulation of the pancreatic

pro-endocrine gene neurogenin3. Diabetes *50*, 928–936. https://doi.org/10.2337/diabetes.50.5.928.

Loomans, C.J.M., Williams Giuliani, N., Balak, J., Ringnalda, F., van Gurp, L., Huch, M., Boj, S.F., Sato, T., Kester, L., de Sousa Lopes, S.M.C., et al. (2018). Expansion of adult human pancreatic tissue yields organoids harboring progenitor cells with endocrine differentiation potential. Stem Cell Rep. *10*, 712–724. https://doi.org/10.1016/j.stemcr.2018.02.005.

Malaguarnera, R., Sacco, A., Voci, C., Pandini, G., Vigneri, R., and Belfiore, A. (2012). Proinsulin binds with high affinity the insulin receptor isoform A and predominantly activates the mitogenic pathway. Endocrinology *153*, 2152–2163. https://doi.org/10.1210/en.2011-1843.

Migliorini, A., Nostro, M.C., and Sneddon, J.B. (2021). Human pluripotent stem cell-derived insulin-producing cells: a regenerative medicine perspective. Cell Metab. *33*, 721–731. https://doi.org/10.1016/j.cmet.2021.03.021.

Murtaugh, L.C., Stanger, B.Z., Kwan, K.M., and Melton, D.A. (2003). Notch signaling controls multiple steps of pancreatic differentiation. Proc. Natl. Acad. Sci. USA *100*, 14920–14925. https://doi.org/10.1073/pnas.2436557100.

Nelson, S.B., Schaffer, A.E., and Sander, M. (2007). The transcription factors Nkx6.1 and Nkx6.2 possess equivalent activities in promoting beta-cell fate specification in Pdx1+ pancreatic progenitor cells. Development *134*, 2491–2500. https://doi.org/10.1242/dev.002691.

Ni, Q., Gu, Y., Xie, Y., Yin, Q., Zhang, H., Nie, A., Li, W., Wang, Y., Ning, G., Wang, W., and Wang, Q. (2017). Raptor regulates functional maturation of murine beta cells. Nat. Commun. *8*, 15755. https://doi.org/10.1038/ncomms15755.

Perko, T., Markočič, E., Knez, Ž., and Škerget, M. (2011). Solubility and diffusivity of CO2 in natural methyl cellulose and sodium carboxymethyl cellulose. J. Chem. Eng. Data *56*, 4040–4044. https://doi.org/10.1021/je200483p.

Qadir, M.M.F., Álvarez-Cubela, S., Klein, D., Lanzoni, G., García-Santana, C., Montalvo, A., Pláceres-Uray, F., Mazza, E.M.C., Ricordi, C., Inverardi, L.A., et al. (2018). P2RY1/ALK3-expressing cells within the adult human exocrine pancreas are BMP-7 expandable and exhibit progenitor-like characteristics. Cell Rep. *22*, 2408–2420. https://doi.org/10.1016/j.celrep.2018.02.006.

Qadir, M.M.F., Álvarez-Cubela, S., Klein, D., van Dijk, J., Muñiz-Anquela, R., Moreno-Hernández, Y.B., Lanzoni, G., Sadiq, S., Navarro-Rubio, B., García, M.T., et al. (2020). Single-cell resolution analysis of the human pancreatic ductal progenitor cell niche. Proc. Natl. Acad. Sci. USA *117*, 10876–10887. https://doi.org/10.1073/pnas.1918314117.

Qi, M., Valiente, L., McFadden, B., Omori, K., Bilbao, S., Juan, J., Rawson, J., Scott, S., Ferreri, K., Mullen, Y., et al. (2015). The choice of enzyme for human pancreas digestion is a critical factor for increasing the success of islet isolation. Transplant. Direct *1*, e14. https://doi.org/10.1097/TXD.0000000000000522.

Rezanejad, H., Ouziel-Yahalom, L., Keyzer, C.A., Sullivan, B.A., Hollister-Lock, J., Li, W.C., Guo, L., Deng, S., Lei, J., Markmann, J., and Bonner-Weir, S. (2018). Heterogeneity of SOX9 and HNF1beta in pancreatic ducts is dynamic. Stem Cell Rep. *10*, 725–738. https://doi.org/10.1016/j.stemcr.2018.01.028.

Sato, T., Stange, D.E., Ferrante, M., Vries, R.G.J., Van Es, J.H., Van den Brink, S., Van Houdt, W.J., Pronk, A., Van Gorp, J., Siersema, P.D., and Clevers, H. (2011). Long-term expansion of epithelial organoids from human colon, adenoma, adenocarcinoma, and Barrett's epithelium. Gastroenterology *141*, 1762–1772. https://doi.org/10.1053/j.gastro.2011.07.050.

Shih, H.P., Kopp, J.L., Sandhu, M., Dubois, C.L., Seymour, P.A., Grapin-Botton, A., and Sander, M. (2012). A Notch-dependent molecular circuitry initiates pancreatic endocrine and ductal cell differentiation. Development *139*, 2488–2499. https://doi.org/10.1242/dev.078634.

Solar, M., Cardalda, C., Houbracken, I., Martín, M., Maestro, M.A., De Medts, N., Xu, X., Grau, V., Heimberg, H., Bouwens, L., and Ferrer, J. (2009). Pancreatic exocrine duct cells give rise to insulin-producing beta cells during embryogenesis but not after birth. Dev. Cell *17*, 849–860. https://doi.org/10.1016/j.devcel.2009.11.003.

Sugiyama, T., Rodriguez, R.T., McLean, G.W., and Kim, S.K. (2007). Conserved markers of fetal pancreatic epithelium permit prospective isolation of islet progenitor cells by FACS. Proc. Natl. Acad. Sci. USA *104*, 175–180. https://doi.org/10.1073/pnas.0609490104.

Takita, M., and Matusmoto, S. (2012). SUITO index for evaluation of clinical islet transplantation. Cell Transplant. *21*, 1341–1347. https://doi.org/10.3727/096368912X636885.

Tremblay, J.R., LeBon, J.M., Luo, A., Quijano, J.C., Wedeken, L., Jou, K., Riggs, A.D., Tirrell, D.A., and Ku, H.T. (2016). In vitro colony assays for characterizing tri-potent progenitor cells isolated from the adult murine pancreas. J. Vis. Exp., 54016. https://doi.org/10.3791/54016.

Wedeken, L., Luo, A., Tremblay, J.R., Rawson, J., Jin, L., Gao, D., Quijano, J., and Ku, H.T. (2017). Adult murine pancreatic progenitors require epidermal growth factor and nicotinamide for self-renewal and differentiation in a serum- and conditioned medium-free culture. Stem Cells Dev. *26*, 599–607. https://doi.org/10.1089/scd.2016.0328.

Xu, X., D'Hoker, J., Stangé, G., Bonné, S., De Leu, N., Xiao, X., Van de Casteele, M., Mellitzer, G., Ling, Z., Pipeleers, D., et al. (2008). Beta cells can be generated from endogenous progenitors in injured adult mouse pancreas. Cell *132*, 197–207. https://doi.org/10.1016/j.cell.2007.12.015.

Zhao, H., Huang, X., Liu, Z., Pu, W., Lv, Z., He, L., Li, Y., Zhou, Q., Lui, K.O., and Zhou, B. (2021). Pre-existing beta cells but not progenitors contribute to new beta cells in the adult pancreas. Nat. Metab. *3*, 352–365. https://doi.org/10.1038/s42255-021-00364-0.

Stem Cell Reports, Volume *18*

Supplemental Information

Methylcellulose colony assay and single-cell micro-manipulation reveal progenitor-like cells in adult human pancreatic ducts

Janine C. Quijano, Lena Wedeken, Jose A. Ortiz, Heather N. Zook, Jeanne M. LeBon, Angela Luo, Jeffrey Rawson, Jacob R. Tremblay, Jacob M. Mares, Kassandra Lopez, Min-Hsuan Chen, Kevin Jou, Carlos Mendez-Dorantes, Ismail H. Al-Abdullah, Debbie C. Thurmond, Fouad Kandeel, Arthur D. Riggs, and Hsun Teresa Ku

SUPPLEMENTARY MATERIALS

This document contains the following information.

1. **Figure S1 to S7.**
2. **Table S1 to S5.**
3. **Supplemental experimental procedures.**
4. **Supplemental references.**

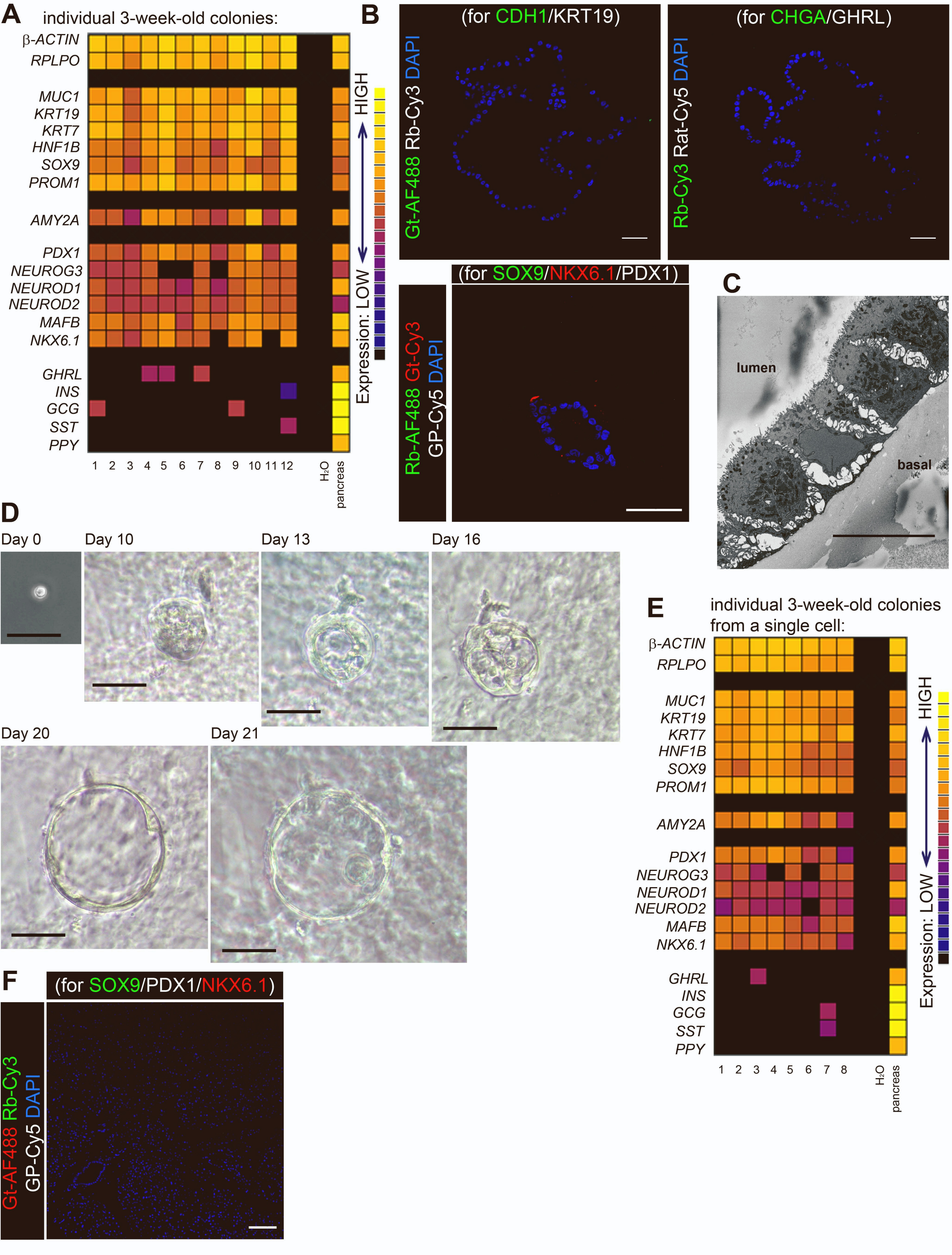
A
individual 3-week-old colonies:
β-ACTIN
RPLPO
MUC1
KRT19
KRT7
HNF1B
SOX9
PROM1
AMY2A
PDX1
NEUROG3
NEUROD1
NEUROD2
MAFB
NKX6.1
GHRL
INS
GCG
SST
PPY
1 2 3 4 5 6 7 8 9 10 11 12
H2O
pancreas
Expression: LOW HIGH
B
(for CDH1/KRT19)
Gt-AF488 Rb-Cy3 DAPI
(for CHGA/GHRL)
Rb-Cy3 Rat-Cy5 DAPI
(for SOX9/NKX6.1/PDX1)
Rb-AF488 Gt-Cy3 GP-Cy5 DAPI
C
lumen
basal
D
Day 0
Day 10
Day 13
Day 16
Day 20
Day 21
E
individual 3-week-old colonies from a single cell:
β-ACTIN
RPLPO
MUC1
KRT19
KRT7
HNF1B
SOX9
PROM1
AMY2A
PDX1
NEUROG3
NEUROD1
NEUROD2
MAFB
NKX6.1
GHRL
INS
GCG
SST
PPY
1 2 3 4 5 6 7 8
H2O
pancreas
Expression: LOW HIGH
F
(for SOX9/PDX1/NKX6.1)
Gt-AF488 Rb-Cy3 GP-Cy5 DAPI

Fig. S1: Characterization of 3-week-old colonies grown in our standard colony assay (associated with Fig. 1 and 2).

(A) Heatmap example of micro-fluidic qRT-PCR from 58 individually micro-manipulated colonies from 7 donors, associated with Fig. 1H. (B) Negative control images for antibodies used in Fig. 1I. Scale bars = 50 μm. (C) A representative image from 3-dimensional scanning electron microscopy (3D-SEM) of a 3-week-old colony derived from unsorted cells grown in the standard colony assay. The lumen of the colony is to the left of cells. Cells in colonies are polarized and ductal-like. Scale bar = 100 μm. (D) Tracking of a single cell to colony formation. The single cell was imaged and then micro-manipulated into a well of a 96-well plate. The resulting colony was identified on Day 10 and tracked to Day 21. Another colony is shown in Fig. 2B. Scale bar = 50 μm for all images. (E) Representative heatmap of micro-fluidic qRT-PCR from 22 (1 cell/well) individually micro-manipulated colonies in 3 independent experiments from 2 donors, associated with Fig. 2E. (F) Negative control image for antibodies used in Fig. 2G. Scale bar = 100 μm.

A

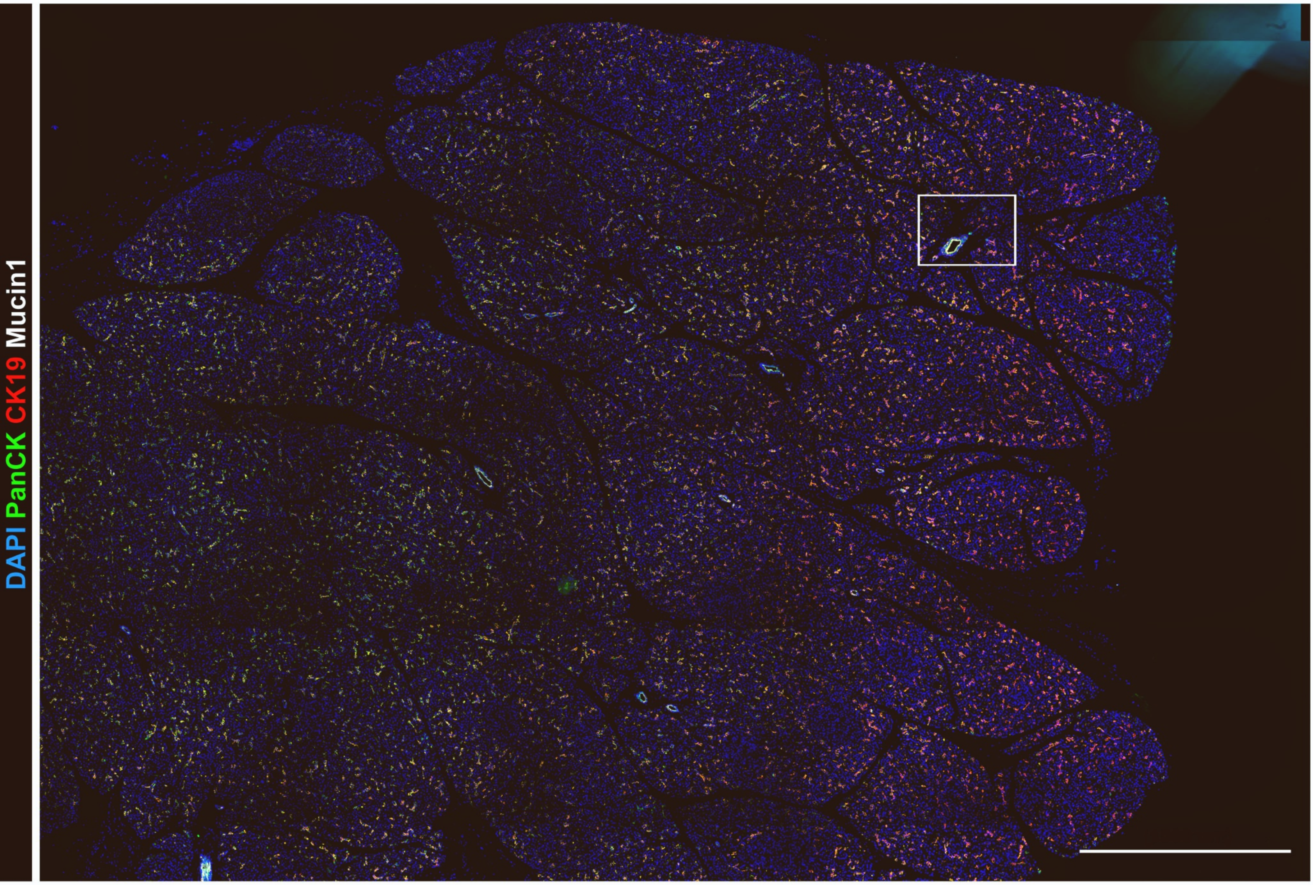

B

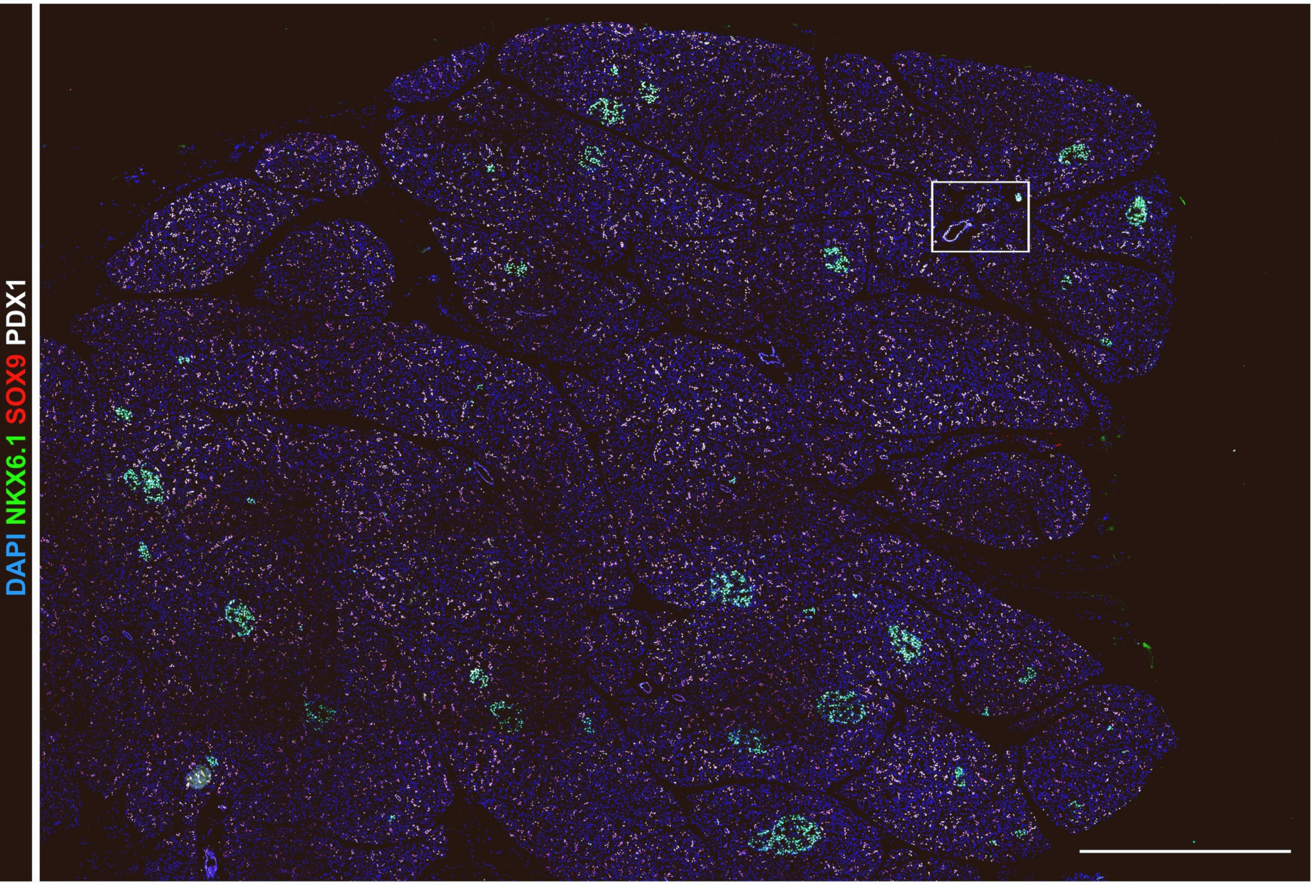

Fig. S2: Stitched images of human pancreas stained with ductal markers or in a sequential slide with SOX9/PDX1/NKX6-1 (associated with Fig. 2F-H).

Examples of sequential slides from a human pancreas that was triple stained for (A) ductal markers Pan-CK (green), CK19 (red) and Mucin1 (white), or (B) NKX6.1 (green), SOX9 (red) and PDX1 (white). Imaging was performed on a Zeiss Observer and the individual images stitched together and presented from 4 different donor tissues. White box indicates areas imaged with a confocal microscope shown in Fig. 2F and 2G. Scale bar = 1 mm. Quantification of SOX9+/PDX1+/NKX6-1 triple positive cells (shown in Fig. 2H) was done using QuPath software.

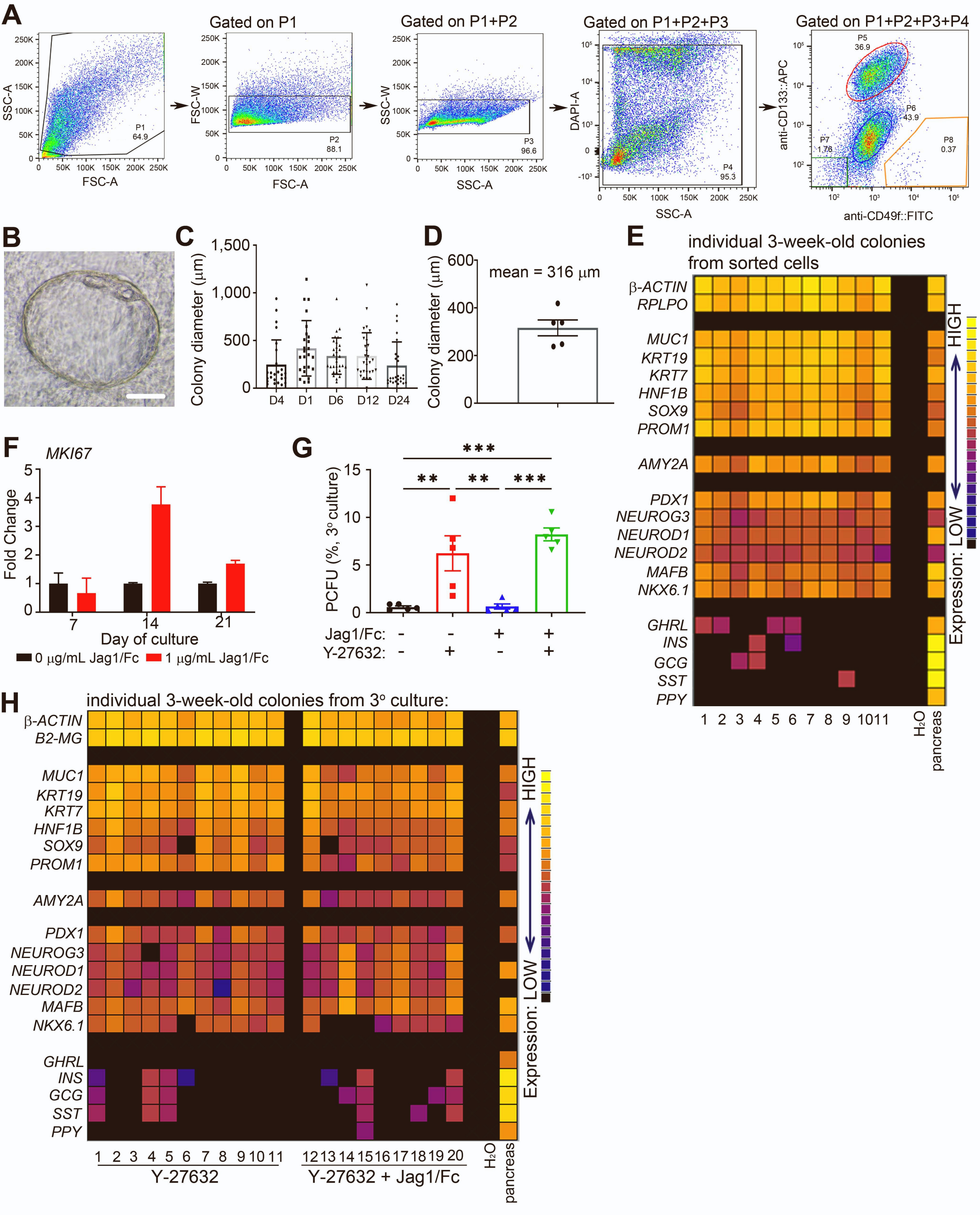
A
Gated on P1
Gated on P1+P2
Gated on P1+P2+P3
Gated on P1+P2+P3+P4
SSC-A
FSC-A
P1 64.9
FSC-W
P2 88.1
SSC-W
SSC-A
P3 96.6
DAPI-A
P4 95.3
anti-CD133::APC
anti-CD49f::FITC
P5 36.9
P6 43.9
P7 1.78
P8 0.37
B
C
Colony diameter (μm)
D4
D1
D6
D12
D24
D
mean = 316 μm
E
individual 3-week-old colonies from sorted cells
β-ACTIN
RPLPO
MUC1
KRT19
KRT7
HNF1B
SOX9
PROM1
AMY2A
PDX1
NEUROG3
NEUROD1
NEUROD2
MAFB
NKX6.1
GHRL
INS
GCG
SST
PPY
1 2 3 4 5 6 7 8 9 10 11
H2O
pancreas
Expression: LOW HIGH
F
MKI67
Fold Change
Day of culture
7
14
21
0 μg/mL Jag1/Fc
1 μg/mL Jag1/Fc
G
PCFU (%, 3° culture)
Jag1/Fc: - - + +
Y-27632: - + - +
H
individual 3-week-old colonies from 3° culture:
β-ACTIN
B2-MG
MUC1
KRT19
KRT7
HNF1B
SOX9
PROM1
AMY2A
PDX1
NEUROG3
NEUROD1
NEUROD2
MAFB
NKX6.1
GHRL
INS
GCG
SST
PPY
1 2 3 4 5 6 7 8 9 10 11
12 13 14 15 16 17 18 19 20
Y-27632
Y-27632 + Jag1/Fc
H2O
pancreas
Expression: LOW HIGH

Fig. S3: Characterization of 3-week-old colonies derived from FACS sorted cells and serially replated colonies in 3º culture (associated with Fig. 3 and 4).

(A) Representative flow cytometry gates for sorting $CD133^{+}CD49f^{low}$ cells. To exclude debris, cells were gated on forward scatter (FSC-A) and side scatter (SSC-A) (population [P] 1). To exclude cell doublets, cells were gated by FSC-W (P2) followed by SSC-W (P3). To exclude dead cells, DAPI-negative live cells were gated (P4). The DAPI positive gate for dead cells was set based on pancreatic exocrine cell samples treated with 70% ethanol (not shown). (B) Representative bright-field image of a 3-week-old colony grown from plated $CD133^{+}CD49f^{low}$ cells. Scale bar = 50 μm. (C) The diameters of individual colonies grown from $CD133^{+}CD49f^{low}$ cells were measured from N=5 different donors and did not show donor-to-donor variation. The average and standard deviation of colony diameters are as follows: Donor 4 (n=25), 248 ± 260 μm; Donor 1 (n=25), 419 ± 291 μm; Donor 6 (n=30), 338 ± 191 μm; Donor 12 (n=29), 338 ± 244 μm; and Donor 24 (n=26), 238 ± 248 μm. (D) The mean diameter of 3-week-old colonies grown from sorted $CD133^{+}CD49f^{low}$ cells was 316 ± 34 μm (mean ± SEM) from N=5 donors analyzed in C. (E) Representative heatmap showing the gene expression profiles of individually micro-manipulated colonies grown from $CD133^{+}CD49f^{low}$ cells were analyzed using microfluidic qRT-PCR analysis. Data are representative of a total of n=38 colonies from N=5 donors tested. (F) Cells stimulated with Jag1/Fc (1 ug/ml, red) or without (0 ug/ml, black) were collected at different timepoints and analyzed for *MKI67* RNA expression. Cells from 1 donor were plated in 4 wells per group. The resulting colonies at designated days were pooled and analyzed by conventional qRT-PCR in technical triplicates. (G) The percent of PCFU among cells plated in 3° culture was higher in the presence of Y-27632 alone or a combination of Y-27632 plus Jag1/Fc, n=5 experiments from 4 donors. (H) A representative heatmap from microfluidic qRT-PCR analysis of individually micro-manipulated colonies from 3° culture treated with Y-27632 alone (n=28 colonies) or a combination of Y-27632 plus Jag1/Fc (n=28 colonies). Data are a representative heatmap from N= 3 donors tested. **p<0.01, ***p<0.001.

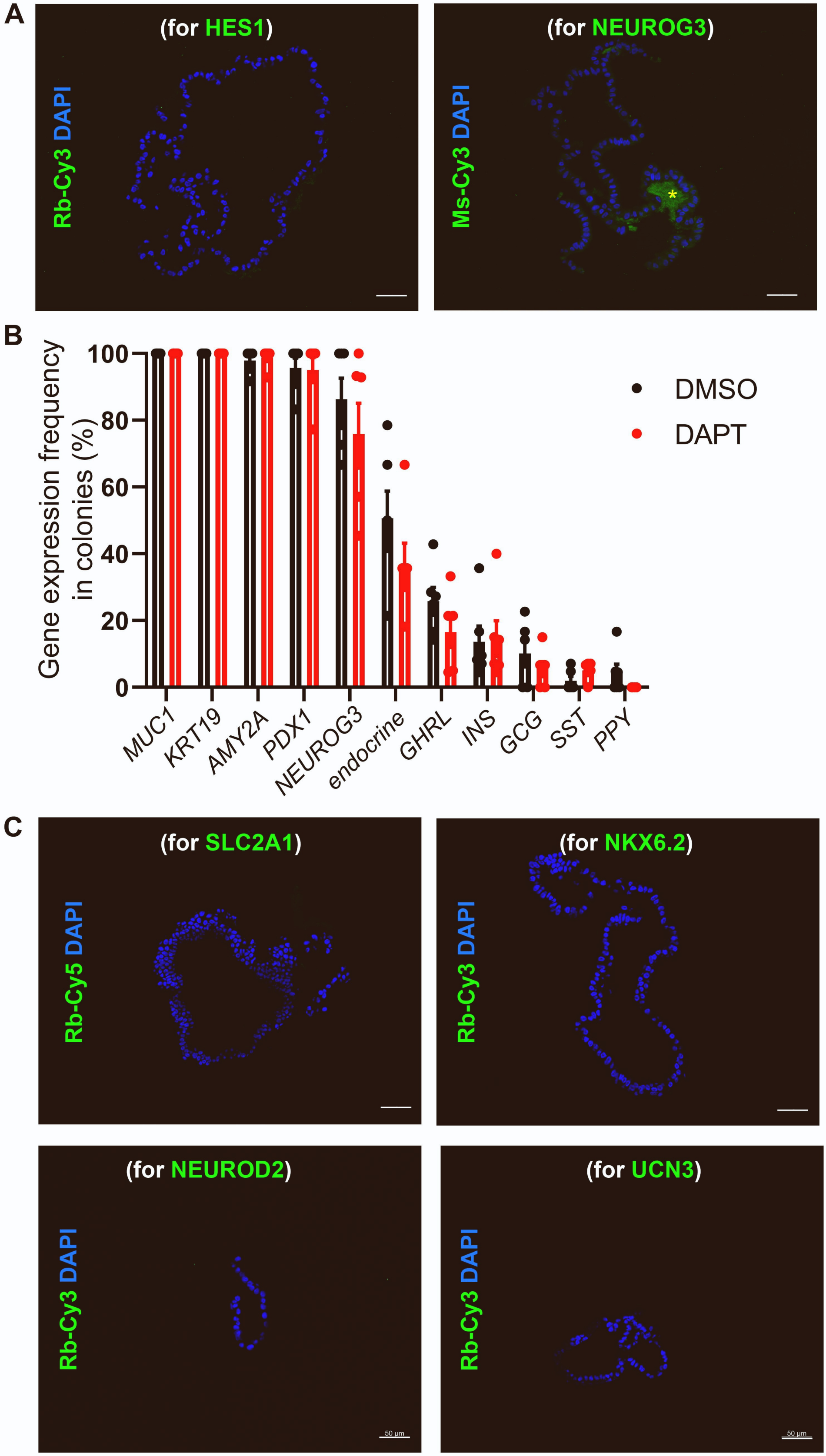

A
(for HES1)
Rb-Cy3 DAPI
(for NEUROG3)
Ms-Cy3 DAPI
*
B
Gene expression frequency in colonies (%)
100
80
60
40
20
0
DMSO
DAPT
MUC1
KRT19
AMY2A
PDX1
NEUROG3
endocrine
GHRL
INS
GCG
SST
PPY
C
(for SLC2A1)
Rb-Cy5 DAPI
(for NKX6.2)
Rb-Cy3 DAPI
(for NEUROD2)
Rb-Cy3 DAPI
(for UCN3)
Rb-Cy3 DAPI

Fig. S4: Characterization of 3-week-old colonies treated with DAPT (a Notch signaling inhibitor) (associated with Fig. 5).

(A) Sequential slides from 3-week-old colonies treated with DAPT shown in Fig. 5C were used for negative control staining. Secondary antibodies for the respective fluorescence were used, as indicated. Yellow star (*) denotes an area of non-specific staining in image on right. Scale bar = 50 μm. (B) Gene expression frequencies among 3-week-old colonies treated with vehicle (DMSO, black) or a Notch signaling inhibitor (DAPT, red). DMSO: n=95 colonies, DAPT: n=100 colonies from N=6 donors. Data represent mean ± standard deviation. Two-way ANOVA, with Sidak's multiple comparison, was used to determine significance. (C) Sequential slides from 3-week-old colonies treated with DAPT shown in Fig. 5H were used for negative control staining. Secondary antibodies for the respective fluorescence were used, as indicated. Scale bar = 50 μm.

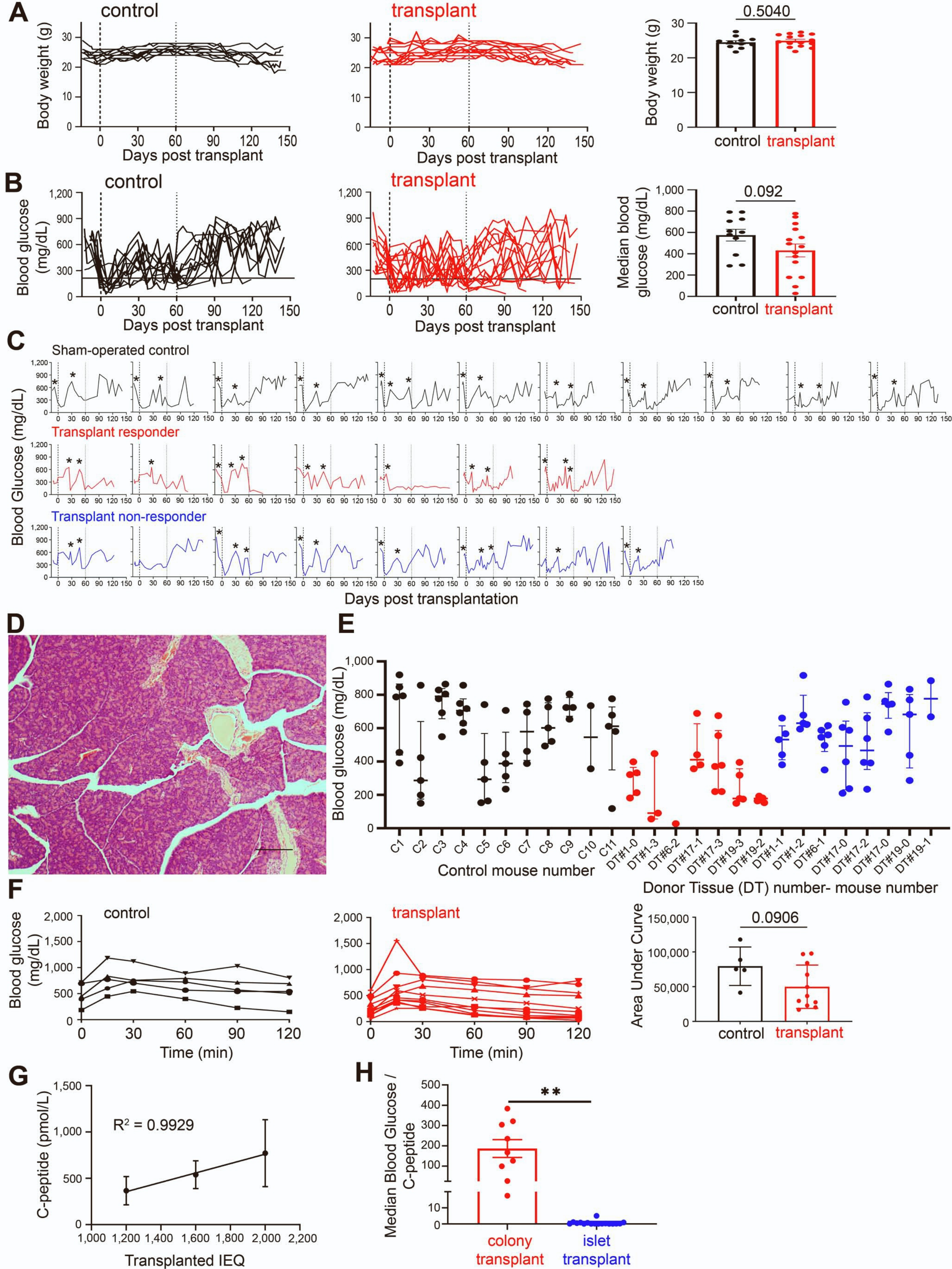
A
control
transplant
Body weight (g)
Days post transplant
0.5040
control
transplant
B
Blood glucose (mg/dL)
Median blood glucose (mg/dL)
0.092
C
Sham-operated control
Transplant responder
Transplant non-responder
Blood Glucose (mg/dL)
Days post transplantation
D
E
Blood glucose (mg/dL)
Control mouse number
Donor Tissue (DT) number- mouse number
C1
C2
C3
C4
C5
C6
C7
C8
C9
C10
C11
DT#1-0
DT#1-3
DT#6-2
DT#17-1
DT#17-3
DT#19-3
DT#19-2
DT#1-1
DT#1-2
DT#6-1
DT#17-0
DT#17-2
DT#17-0
DT#19-0
DT#19-1
F
Blood glucose (mg/dL)
Time (min)
Area Under Curve
0.0906
G
C-peptide (pmol/L)
R² = 0.9929
Transplanted IEQ
H
Median Blood Glucose / C-peptide
**
colony transplant
islet transplant

Fig. S5: Additional data associated with transplantation studies (associated with Fig. 6).
(A) Body weight measurements over time from individual sham-operated control (black) and transplant (red) mice. No difference was found. Data represent mean ± SEM. (B) Blood glucose measurements over time grouped by sham-operated control (black) or transplant (red) mice. Median blood glucose levels (90+ days post-transplantation) of transplant mice trended lower than sham-operated control mice. Data represent median ± SEM. (C) Blood glucose measurements over time from individual mice from sham-operated controls (black), transplant responders (red) and non-responders (blue). Asterisks (*) indicates the day that an insulin pellet was inserted into the mouse. (D) H&E staining of STZ-treated NOD-SCID pancreas 4 months post-transplantation; a representative mouse that received colonies under kidney capsule is shown. Scale bar = 200 μm. (E) Blood glucose measurements from mice between days 90 and 120 post-transplantation. Data represent median ± standard deviation of fasting blood glucose measured at least once per week. (F) An intraperitoneal glucose tolerance test (IP-GTT) was performed 3 months post-transplantation. Fasting blood glucose levels (mg/dL) were graphed, grouping together sham-operated mice (black) and transplant (red) mice. The area under the curve (AUC) of transplant mice trends lower than control mice. Data represent mean ± standard deviation from 5 control and 11 transplant mice. (G) Human islets were transplanted into the kidney capsule of STZ-treated NOD-SCID mice. Serum C-peptide levels were analyzed between 90-150 days post-transplantation. Abbreviation: islet equivalent, IEQ. (H) Ratio of the median blood glucose and measured c-peptide concentration in individual mice indicate that all mice transplanted with DAPT-treated colonies are significantly different than mice transplanted with human islets. **$p<0.01$.

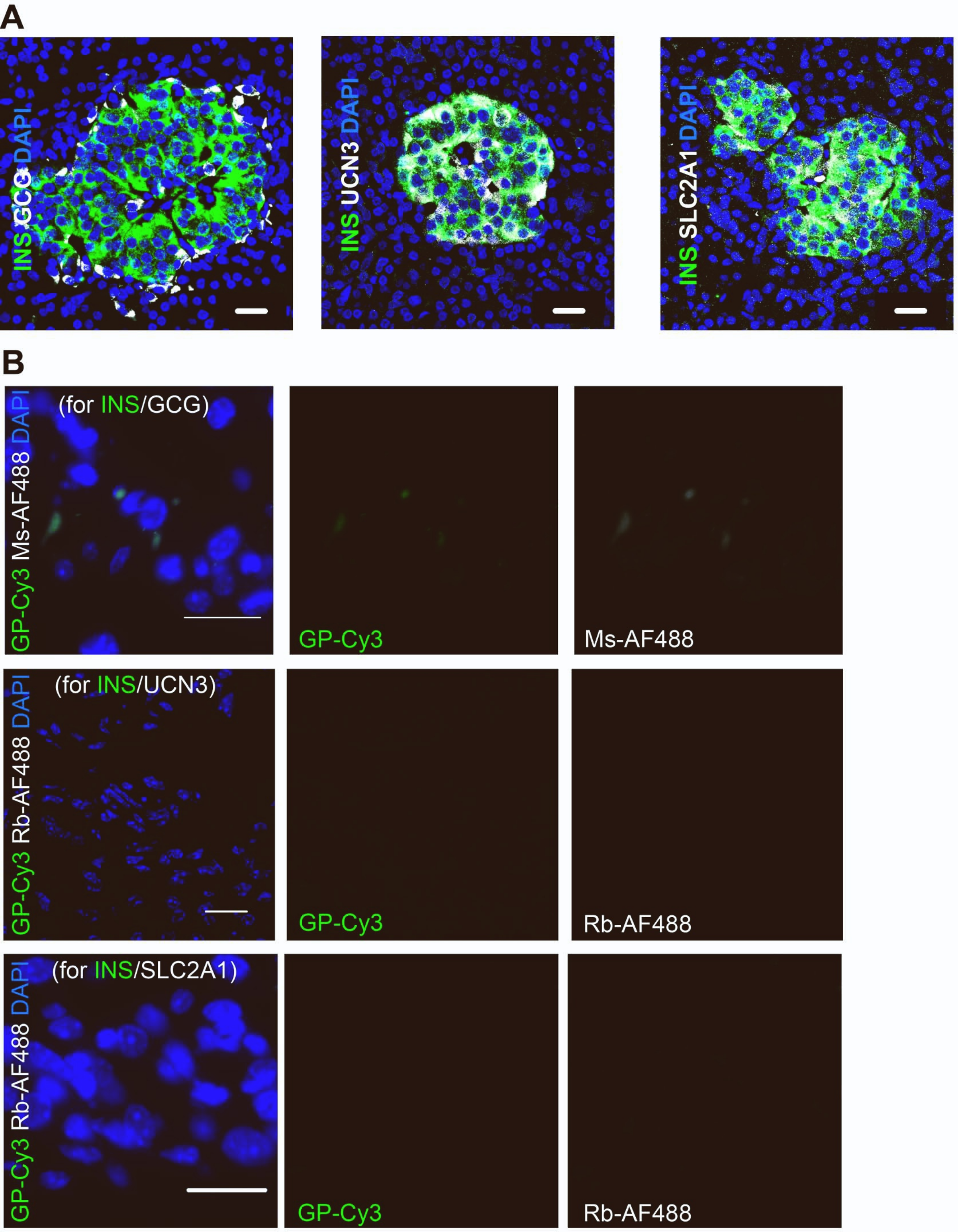
A
INS GCG DAPI
INS UCN3 DAPI
INS SLC2A1 DAPI
B
GP-Cy3 Ms-AF488 DAPI
(for INS/GCG)
GP-Cy3
Ms-AF488
GP-Cy3 Rb-AF488 DAPI
(for INS/UCN3)
GP-Cy3
Rb-AF488
GP-Cy3 Rb-AF488 DAPI
(for INS/SLC2A1)
GP-Cy3
Rb-AF488

Fig. S6: Validation of immunofluorescence staining of the grafts with positive and negative controls (associated with Fig. 6).

(A) Positive control staining. Left: a human islet stained with INS (green) and GCG (white). Middle: a human islet stained with INS (green) and UCN3 (white). Right: a human islet stained with INS (green) and SLC2A1 (white). All nuclei are stained with DAPI (blue). The same antibodies and conditions were used for staining kidney grafts in Fig. 6H-K. Scale bars = 20 μm. (B) Sequential slides from grafts shown in Fig. 6H-K were used for negative control staining (secondary antibodies only). Top row: INS (anti-guinea pig) and GCG (anti-mouse) secondary antibodies. Middle row: INS (anti-guinea pig) and UCN3 (anti-rabbit) secondary antibodies. Bottom row: INS (anti-guinea pig) and SLC2A1 (anti-rabbit) secondary antibodies. Scale bars = 20 μm.

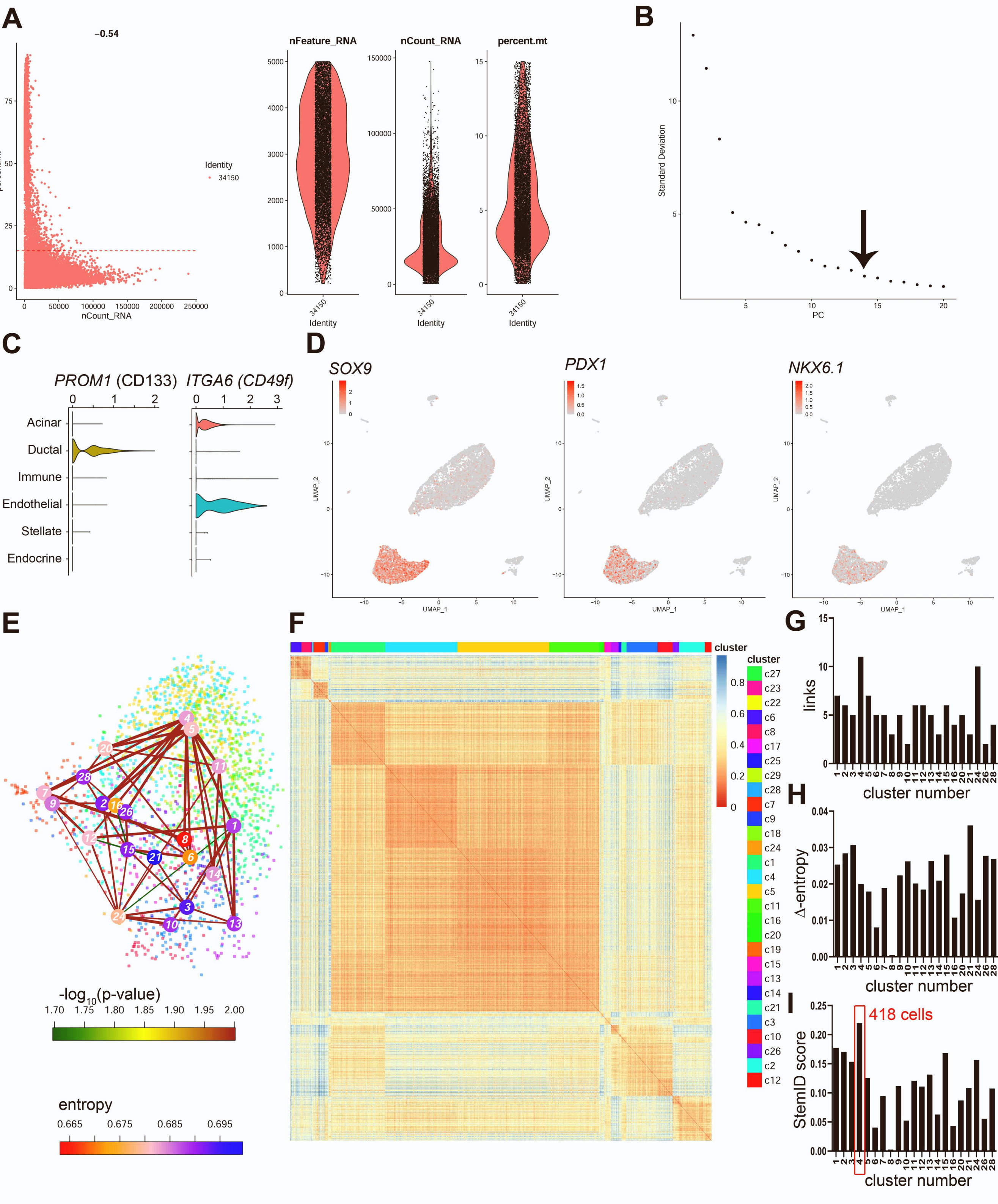

A
−0.54
nCount_RNA
Identity
34150
nFeature_RNA
nCount_RNA
percent.mt
B
Standard Deviation
PC
C
PROM1 (CD133)
ITGA6 (CD49f)
Acinar
Ductal
Immune
Endothelial
Stellate
Endocrine
D
SOX9
PDX1
NKX6.1
UMAP_1
UMAP_2
E
-log10(p-value)
entropy
F
cluster
G
links
cluster number
H
Δ-entropy
cluster number
I
418 cells
StemID score
cluster number

Figure S7. Single-cell RNA-sequencing (scRNAseq) analysis of uncultured and dissociated exocrine tissue from adult human pancreas (associated with Fig. 7).

(A) Quality control analysis of scRNAseq data. Events that exceeded higher than 15% mitochondria genes, less than 200 RNA counts and more than 5,000 feature genes were excluded from subsequent analysis. (B) Elbow-plot; PC number 14 was used for clustering for total cells. (C) Ductal cell cluster expressed high levels of *PROM1* (CD133) but low amount of *ITGA6* (CD49f), consistent to flow cytometry analysis (Fig 3B). (D) UMAPs showing the gene expression of the *SOX9*, *PDX1* or *NKX6.1* within the total cells. The majority of the detected cells are within the ductal cluster. (E-I) Quality control for StemID analysis. (E) RaceID2 clusters, connected by links. (F) Upper left is showing a heatmap of cell-to-cell transcriptome distances. The color of the dots indicates the –log10 p-value and the color of the vertices indicates the entropy. (G) The number of links per cluster, (H) median transcriptome entropy, and the (I) resulting StemID score are shown in bar graphs, with cluster 4 indicated by the red box having the highest value; this bar represents 418 cells.

Table S1: List of donor information, related to Figures 1, 2, 3, 4, 5, 6 and 7.

Donor tissue[1]	Age (years)	BMI (kg/m^2)	HbA1c (%)	Gender	Ethnicity	Figure #
1	16	23.5	5.3	Male	Caucasian	1c, 1d, 1e, 1g, 1h, 1i, 3f, 3g, 4e, 5b, 5e, 6b, 6c, 6d, 6e, 6f, s1c, s3c, s3d, s5a, s5b, s5c, s5d, s5f, s5g, s5i
2	25	25.3	5.3	Male	Caucasian	1c, 1d, 1e, 2h, s3f
3	26	24.6	5.1	Male	Caucasian	1c, 1d, 1e, 4b, 4c, 4d, 5e, s3g
4	52	31.5	n/a	Female	Caucasian	1c, 1d, 1e, 1h, 3f, 4e, s3c, s3d
5	52	32.4	5	Male	Hispanic	1c, 1d, 1e, 1h, 4e, s3c, s3d
6	24	24.6	5	Male	Caucasian	1c, 1f, 1i, 4b, 4c, 4d, 4e, 5b, 5c, 5d, 5g, 5h, 5i, 5j, 6b, 6c, 6d, 6e, 6h, 6i, s3g, s4a, s4b, s4c, s5a, s5b, s5c, s5d, s5f, s5g, s5i, s6b
7	27	31.4	5.4	Male	African American	1c, 2c, 2d, 2e
8	45	38	5.3	Female	Caucasian	1c, 1h, 2c, 2d, 2e, 2h, 3d, 3e, 3f, 4e, s1a, s1e, s3e
9	59	35.6	n/a	Female	Caucasian	1c, 3b, 3c, 3e
10	24	32	4.7	Male	Caucasian	1c, 3b, 3c, 3e
11	29	33.9	4.2	Male	Hispanic	1c, 3b, 3c, 3e, s3a, s3c, s3d
12	24	50.5	5.6	Male	Caucasian	1c, 3b, 3c, 3e, 3f, s3b
13	41	19.1	4.7	Female	Caucasian	1c, 3b, 3c, 3e
14	36	36.6	5.3	Male	Hispanic	1c, 3c, 3e, 5e, 5f, 5g, s4b
15	53	27.7	5.5	Female	Caucasian	3c, 3e, 4e
16	17	39.4	5.1	Male	Hispanic	1c, 3c, 3e
17	35	27.4	5.4	Male	Caucasian	1c, 4e, 5b, 5e, 5f, 6b, 6c, 6d, 6e, s5a, s5b, s5c, s5d, s5f, s5g, s5i
18	60	37.9	4.5	Male	Caucasian	1c, 5b, 5e, 5f, 5g, s4b
19	65	35.1	5.6	Female	African American	1c, 4b, 4c, 4d, 5b, 5e, 5f, 5g, 6b, 6c, 6d, 6e, 6g, 6j, 6k, s3g, s4b, s5a, s5b, s5c, s5d, s5f, s5g, s5i, s6b
20	23	20.6	5.5	Male	African American	1c, 1i, 5b, 5d, 5e, 5f, 5g, 5h, 5i, s1b, s4b
21	50	21.5	4.9	Female	Caucasian	1c, s3g
22	39	30	5	Male	Caucasian	1c, 4b, 4c, 4d, s3g
23	61	42.1	n/a	Male	Hispanic	1c, s3c, s3d
24	47	29.3	5.1	Male	Hispanic	1c, 1h, 4e
25	34	31.3	5.1	Male	Caucasian	1c, 1i, 5b, 5d, 5e, 5h, 5i, s4c
26	23	32.9	4.7	Male	Hispanic	1c, 1i, 5b, 5d, 5e, 5h, 5i, s4c
27	27	30	5.2	Male	Hispanic	1c
28	57	26	5.4	Female	Hispanic	1c, 5b
29	24	24	5.2	Female	Hispanic	1c, 5b, 5e, 5f
30	23	36.1	5.1	Male	Hispanic	1c, 7, s7
31	28	21	5.5	Male	Caucasian	1c
32	22	31.3	5.5	Male	Hispanic	1b, 1c
33	15	24.5	5.1	Male	Caucasian	1c
34	40	36	5	Female	Hispanic	1c
35	46	30	5.6	Female	Caucasian	1c
36	38	23.8	5.1	Male	Hispanic	1i, 5e, 5f, s1b
37	26	28.6	4.8	Male	Hispanic	3f
38	26	24.4	5.2	Male	Hispanic	5g, s4b
39	50	36.2	4.9	Male	Caucasian	2b, 2c, 2d, s1d

40	44	24.38	5.3	Male	n/a	2h
41	18	35.5	5.2	Male	n/a	2f, 2g, 2h, s1f, s2a, s2b
42	17	32.2	5.5	male	Caucasian	S5H
43	55	28.6	5.6	male	Caucasian	S5H
44	42	35	5.4	male	Hispanic	S5H

[1]: Donor numbers 1-41 are from exocrine tissues that were used for progenitor cell studies. Donor numbers 42-44 are from islets that were used as positive control for transplantation studies.

Table S2: Summary of donor characteristics on exocrine tissue (donor numbers 1-41), related to Figures 1, 2, 3, 4, 5, 6 and 7.

	Mean ± SD	Lower limit	Upper limit
Age (years)	36 ± 14	15	65
Body Mass Index (BMI) (kg/m^2)	30.4 ± 6.6	19.1	50.5
HbA1c (%)	5.1 ± 0.3	4.2	5.6
Gender: male	73%	n/a	n/a
Gender: female	27%	n/a	n/a
Ethnicity[1]: Caucasian	51%	n/a	n/a
Ethnicity[1]: Hispanic	41%	n/a	n/a
Ethnicity[1]: African American	8%	n/a	n/a

[1]: When available in documentation; n=39 donors

Table S3: List of cell culture components used for the standard colony assay, related to Figures 1, 2, 3, 4, 5, 6 and 7.

Name	Stock Concentration	Final Concentration	Company	Catalog number
Methylcellulose, 1500 cPs	3.3% in DMEM/F12	1%	Shin-Etsu	9004-67-5
Matrigel	100% (8-12 mg/ml)	5%	Corning	356231
KnockOut Serum Replacement	100%	10%	ThermoFisher	10828-028
Nicotinamide	1 M	10 mM	Sigma	N0636
Exendin 4	0.1 mM	0.1 nM	Sigma	E7144
SB 202190	100 mM	10 μM	Sigma	S7067
Gastrin II Sulfated	1 mM	100 nM	Sigma	G1260
rm R-Spondin 1	250 mg/ml	750 ng/ml	R&D	3474-RS
rh VEGF	10 mg/ml	10 ng/ml	R&D	293-VE
rh EGF	25 mg/ml	50 ng/ml	R&D	236-EG
rm Noggin	10 mg/ml	100 ng/ml	R&D	1967-NG
A 83-01	50 mM	500 nM	Tocris	2939
rh/m/r Activin B[1]	10 mg/ml	10 ng/ml	R&D	6905-AB

[1]: Activin B is included in culture media for Fig. 1, 2, 3, S1, S3, and Movie 1.

Table S4: List of primary antibodies used for immune-fluorescent staining, related to Figures 1, 2, 5 and 6.

Antibody	Company	Catalog Number	RRID
Hamster anti-Mucin	Neomarkers/ThermoFisher	1630-PI	AB_11000874
Rabbit anti-Amylase	Sigma	A8373	AB_258380
Guinea pig anti-PDX1	Abcam	Ab47308	AB_777178
Rabbit anti-Ghrelin	Phoenix	H-031-031	AB_2314558
Mouse anti-NEUROG3	DSHB	F25A1B3	AB_528401
Rabbit anti-Glut1	Abcam	Ab15309	AB_301844
Guinea pig anti-Insulin	Dako/Agilent	A0564	AB_10013624
Mouse anti-Glucagon	Sigma	G2654	AB_259852
Goat anti-Ecad	R&D Systems	AF748	AB_355568
Rabbit anti-KRT19	Abcam	ab52625	AB_2281020
Mouse anti-pan-CK	DAKO	M3515	AB_2132885
Rabbit anti-Urocortin III (Ucn3)	Phoenix Pharmaceuticals	H-019-28	AB_2889826
Rabbit anti-Hes1 (D6P2U)	Cell signaling	11988S	AB_2728766
Rabbit anti-Neurod2	Abcam	ab104430	AB_10975628
Rabbit anti-Nkx6-2	Thermofisher	PA5-103900	AB_2853232
Rabbit anti-Chromogranin A (ChrA)	Novus	NB120-15160	AB_789299
Goat anti-NKX6.1	Novus/R&D Systems	AF5857	AB_1857045
Rabbit anti-Sox9	Chemicon/Milipore-Sigma	AB5535	AB_2239761

Table S5: List of Taqman probes used for conventional and micro-fluidic qRT-PCR, related to Figures 1, 2, 3, 4, and 5.

Human gene	Assay ID
Beta-actin	Hs01060665_g1
B2-MG	Hs00984230_m1
RPLPO	Hs99999902_m1
KRT19	Hs00761767_s1
KRT7	Hs00818825_m1
MUC1	Hs00159357_m1
HNF1B	Hs01001602_m1
SOX9	Hs00165814_m1
PROM1 (CD133)	Hs01009259_m1
AMY2A	Hs00420710_g1
CPA1	Hs01056157_m1
HES1	Hs00172878_m1
SLC2A1 (GLUT1)	Hs00892681_m1
PTPRC (CD45)	Hs00236304_m1
KDR	Hs00176676_m1
INS	Hs00355773_m1 (microfluidic), Hs02741908_m1 (conventional)
GCG	Hs00174967_m1
PPY	Hs01078030_m1
SST	Hs00356144_m1
GHRL	Hs00175082_m1
PDX1	Hs00236830_m1
NEUROG3 (NGN3)	Hs01875204_s1
NEUROD1	Hs01922995_s1
NEUROD2	Hs00272055_s1
MAFB	Hs00534343_s1
NKX6.1	Hs00232355_m1
NKX6.2	Hs00752986_s1
UCN3	Hs00846499_s1

Supplemental Experimental Procedures:

Single-cell suspension. Donated pancreata were procured and shipped to City of Hope for isolation of islets as previously described (1). All tissues used in this study had consent for research from close relatives of the donors. After islet removal, de-identified human pancreata were obtained from the Southern California Islet Cell Resource (SC-ICR) Center at the City of Hope. The tissue was rinsed once in cold PBS, resuspended in Dulbecco's phosphate-buffered saline (DPBS) containing 0.1% bovine serum albumin (BSA), collagenase B (2-4 mg/ml) (Roche, Mannheim, Germany), and DNase I (2,000 U/ml) (Calbiochem, Darmstadt, Germany), and incubated at 37°C for 20 min, during which time the tissue was gently disrupted every 5-10 min using a 16G syringe needle. Cells were washed twice in DPBS / 0.1% BSA / 2,000 U/ml DNase I and filtered through a 40 µm nylon mesh (BD Biosciences, San Jose, CA) to yield a single-cell suspension before cryopreservation, culture, or antibody staining.

Freezing and thawing of single cells. Dissociated single-cell suspensions were counted, resuspended in Cryostor (BioLife Solutions), and frozen using a programed cell freezer before being transferred to a liquid nitrogen tank for long-term storage. To thaw, frozen vials were placed in a 37°C water bath for 2 minutes. Thawed cells were transferred to a 15 ml conical tube and washed once with warm PBS / 0.1% BSA before culture.

Flow cytometry and cell sorting. Cell suspensions were blocked with human gamma globulin (IgG) (22.8 µg/ml) (Jackson ImmunoResearch, West Grove, PA) for 15 min on ice. Biotin-conjugated anti-human CD133/2 (clone 293C3; Miltenyi Biotec) and fluorescein (FITC)-conjugated anti-human/mouse CD49f (clone GoH3; BioLegend) antibodies were added to cells. After 20 min on ice, cells were washed twice, treated with streptavidin-labeled allophycocyanin (APC) (2 µg/ml BioLegend, San Diego, CA) for 15 min on ice, washed twice, and resuspended in PBS / BSA / DNase I containing 4',6-diamidine-2'-phenylindole dihydrochloride (DAPI, 0.2 µg/ml). Control antibodies used were biotin-conjugated mouse IgG_{2b} (Clone eBMG2b; eBioscience) and FITC-conjugated rat IgG_{2a} (BioLegend). Cell sorting was performed on an Aria-special order research product (SORP) (Becton Dickinson, San Jose, CA). All analyses included an initial gating of forward (FSC) and side (SSC) scatters to exclude debris. Sorting further excluded doublets by gating out high pulse-width cells; live cells were selected by negative staining with DAPI. The purity of the sorted events was routinely > 95%. Acquired flow data were analyzed using software provided by FlowJo (TreeStar, Ashland, OR).

***In vitro* colony assay.** Cells were cultured at a density between 1×10^3 to 5×10^3 cells in 0.5 ml per well as previously described (2). The standard culture medium for adult human cells contained DMEM/F12, KnockOut Serum Replacement, methylcellulose, Matrigel, nicotinamide, Noggin, epidermal growth factor (EGF), A83-01, SB202190, Rspondin-1 (RSPO1), exendin-4, vascular endothelial growth factor-A (VEGF), gastrin II (sulfated), and activin B (see Table S3 for concentrations and sources). Because transforming growth factor (TGF)-beta signaling is agonized by activin B and antagonized by A83-01, we later determined that activin B was dispensable (data not shown). Cells were plated in 24-well ultra-low protein-binding plates (Corning, New York, USA) and incubated in a humidified 5% CO_2 atmosphere at 37°C. Addition of N-[N-(3,5-Difluorophenacetyl)-L-alanyl]- S-phenylglycine t-butyl ester (DAPT) on day 10 was accomplished by distributing 50 µL of DAPT solution per well on top of the semisolid medium. The number of colonies was counted 3 weeks post-plating. Percent PCFU was calculated by dividing the number of colonies formed by the total number of cells plated per well.

Analysis of colony size and cell number. Individual colonies were imaged using Infinity Analyze software (Lumenera Corporation) and colony diameters were measured. Colonies were assigned to a category (small, medium, or large; 10 colonies per group with 2 technical replicates), hand-picked, and pooled into wells in a 96-well plate. Colonies were dissociated into single cells by incubating with 0.25% trypsin-EDTA at 37°C for 5 min, followed by gentle pipetting. Single cells were counted using a hemocytometer.

Micro-manipulation of single colonies or cells. Three-week-old colonies were visualized under a microscope, lifted one-by-one using a 10-µL pipette tip in a volume of ~2 µL, and processed for microfluidic qRT-PCR analysis (Fig. 1H, analysis procedure described below) (2). To micro-manipulate single cells, using a method similar to reported by Suda et al. (3), cells were first placed in a semisolid

medium in DMEM/F12 with 1% methylcellulose and 10% Knockout Serum at a density of 3,000 cells per ml in a 35-mm petri dish. Individual cells were visualized under a microscope and lifted one-by-one using a fine Pasteur pipet with a diameter of ~30 µm at the opening. Each cell was transferred into one well in a low-binding 96-well plate containing 100 µL of standard semisolid medium (Fig. 2A). The presence of a single colony was confirmed by visualization under a microscope. In some experiments, micro-manipulated single cells were processed for microfluidic qRT-PCR analysis (Fig. 3D).

In vitro self-renewal assay. Cells were plated into our standard colony assay (1° culture). Jag1/Fc (R&D Systems #1277), a Notch ligand, or Y-27632 (Stemgent #04-0012-10), a ROCK inhibitor, was added at a concentration of 1 µg/ml or 10 µM, respectively. Three weeks later, colonies were collected, pooled, washed in warm PBS/0.1% BSA, and dissociated into single cells by incubating with 0.25% trypsin-EDTA at 37°C for 5 min, followed by gentle pipetting. Single cells were counted, diluted, and a fraction of the cells were re-plated into a new 24-well plate at a concentration of 5×10^3 cells in 0.5 ml per well (2° culture). This pooling, dissociation, and re-plating was repeated once more into 3° culture. The final total number of PCFUs was calculated by multiplying the previous dilution factor(s) with the number of colonies per well in the present culture. The fold change of PCFUs was calculated by the total number of PCFUs in the culture divided by the total number of PCFUs in the 1° culture.

***In vivo* transplantation.** Three-week-old colonies that were treated with 10 µM DAPT on day 10 were collected on day 21, pooled, partially digested in 0.063% trypsin-EDTA at 37°C for 3 min, gently pipetted, washed, and concentrated (4). An aliquot was further dissociated into single cell suspension and counted with a hemocytometer to estimate cell number per graft. Three weeks before transplantation, NOD-SCID mice (8-week-old males) were injected with streptozotocin (STZ; 45 mg/kg body weight; freshly made in 100 mM Na-citrate buffer, pH 4.5) for 3 consecutive days to induce insulin-dependent diabetes (5). Hyperglycemia was defined as fasting blood glucose >200 mg/dl and was measured using a HemoCue Glucose 201 (HemoCue). Approximately 3,500 colonies (1-2.5 $x10^6$ cells) were placed under the renal capsule for each mouse; colonies from a single donor were transplanted into 2 to 4 mice (n=15 mice from 4 donor tissues). Fasting blood glucose was monitored weekly; mice were placed in a new cage without food and blood glucose measured 5 hours later. When a mouse had a blood glucose level higher than 450 mg/dL an insulin pellet was inserted within the first 60 days. Data collection on function of the beta cells occurred after 90 days to allow for a washout of the exogenous insulin.

Colonies from four different donor tissues were transplanted into mice in independent experiments. Control mice were age-matched, received STZ injections, and were subjected to sham operations. Mice with the top 8 median blood glucose were separated from the bottom 7, representing the non-responder and responder transplants, respectively.

***In vivo* glucose-stimulated insulin secretion (GSIS).** Mice were fasted for 6 hours in a clean cage before the first blood collection. D-glucose was then given intraperitoneally at 2 g/kg to each mouse, and blood serum was collected after 60 minutes. Concentrations of C-peptide were measured using the human C-peptide Ultrasensitive ELISA kit (Mercodia, Winston Salem, NC). The amount of C-peptide secretion was expressed as fold-change of C-peptide concentration before and after glucose bolus, from the same mouse.

***In vivo* intra-peritoneal glucose tolerance test (IP-GTT).** Levels of blood glucose were measured at 0, 15, 30, 60, 90, and 120 min using a HemoCue after an i.p. injection of D-glucose (2 g/kg) to a mouse that had fasted for 6 hours in a clean cage.

Immunofluorescence staining. For paraffin-embedded formalin-fixed graft tissues, samples were cut to 5 µm thickness, de-waxed in xylene and rehydrated in ethanol. Antigen retrieval was performed by heating samples in a microwave oven for 10 min in 200 ml of sodium citrate buffer, 6.0 pH (Vector Laboratories, H-3300). Samples were incubated with blocking buffer supplemented with 5% donkey serum and 0.1% Triton X-100 at for 2 hours at room temperature. Primary antibodies, as listed in Table S4, were added and samples were incubated at 4°C overnight**.** Slides were washed with PBS/0.1% Triton X-100 and incubated with donkey-raised secondary antibodies conjugated to Cy3, Cy5, DyLight488, AlexaFluor488, or AlexaFluor647 (Jackson ImmunoResearch), for 2 hours at room temperature.

Autofluorescence was minimized using TrueView kit (Vector Laboratories, SP-8400-15) and mounted with Vectashield vibrance antifade mounting medium (Vector Laboratories, H-1700)

For whole-mount immunofluorescent staining, colonies were collected, pooled, and incubated in Cell Recovery Solution (Corning, 354253) for 30 min on ice, fixed in 4% paraformaldehyde with 0.15% Triton-X 100x at 4°C overnight, and incubated with blocking buffer, primary and secondary antibodies as described above.

For frozen sections, colonies were collected, pooled, and incubated with Cell Recovery Solution for 30 minutes on ice. Next, colonies were fixed in 4% paraformaldehyde with 0.15% Triton X-100 at 4°C overnight. Colonies were then cryoprotected in 30% sucrose with PBS at 4°C overnight, embedded in Optimal Cutting Temperature compound (Fisher Scientific, 23-730-571), and frozen into blocks. Samples were cut into 8 µm thickness and thawed at room temperature; thawed samples were washed with PBS. Antigen retrieval was necessary for anti-NEUROG3 (at pH 5.5) as well as anti-NEUROD2 and anti-UCN3 (at pH 8.0) detection. For antibodies with high background, slides were blocked with buffer containing 5% donkey serum (DS) or 10% DS + 1x Universal Blocking Reagent (BioGenex #HK085-5K), and 0.1% Triton X-100 for 1 hour at room temperature. Next, slides were incubated with primary and secondary antibodies as described above.

Images were captured on a Zeiss LSM880 with Airyscan, Zeiss Axio-Observer-Z1 with Apotome, or Zeiss Axio-Cam506 Mono for tiling images. Figures were prepared using LSM Image Browser software (Carl Zeiss, Germany) and Photoshop (Adobe). Quantification of images was performed with QuPath software to identify total cells (DAPI) and positive cell staining (6).

Conventional or microfluidic quantitative reverse transcription-polymerase chain reaction (qRT-PCR). Total RNA extraction, reverse transcription, and conventional qRT-PCR analyses were performed as previously described (7). β-actin was used as an internal control for normalization. Three technical replicates were used in all PCR runs.

Microfluidic qRT-PCR was performed using the BioMark 48.48 Dynamic Array system (Fluidigm-Biomark). Individual micro-manipulated cells or colonies were collected in 10 µL of reaction buffer (CellsDirect One-Step qRT-PCR Kit, Invitrogen, Carlsbad, CA) and preamplified (14 cycles for single colonies or 22 cycles for single cells) according to the manufacturer's instructions (Fluidigm, Invitrogen). Amplified cDNA was loaded onto a 48.48 Dynamic Array using the NanoFlex integrated fluidic circuit (IFC) controller (Fluidigm). Threshold cycle (Ct) was used to measure fluorescence intensity. Ct values were determined by the BioMark PCR analysis software (Fluidigm) and expressed either as a heat map or relative expression (delta Ct), of the gene of interest to internal control gene. In a heat map, the cooler and warmer colors represent lower and higher expression, respectively. All reactions were performed with negative (water) and positive (cDNA from adult human pancreatic cells) controls in all experiments. Taqman probes (Life Technologies, Carlsbad, CA) and their catalog numbers are listed in Table S5. Gene expression frequency in colonies (%) was obtained from the number of colonies expressing a gene divided by the total number of colonies examined. Also, the 'endocrine' category is the percent of colonies expressing either *INS*, *GCG*, *SST*, *PPY* or *GHRL* gene.

Electron microscopy. Single colonies were collected, pooled, and fixed in 2% glutaraldehyde in 0.1 M cacodylate buffer [$Na(CH_3)_2AsO_2$•$3H_2O$; pH7.2] at 4°C overnight. Colonies were washed with 0.1 M cacodylate buffer (pH 7.2), post-fixed with 1% OsO_4 in 0.1 M cacodylate buffer for 30 min, and washed with 0.1 M cacodylate buffer. Samples were dehydrated, embedded in EMbed 812 (Electron Microscopy Sciences, Hatfield, PA), and polymerized at 64°C for 48 h. Ultrathin sections (70-nm thickness) were cut using a Leica ultramicrotome with a diamond knife, transferred to 200-mesh EM grids, and stained with 2% uranyl acetate in 70% ethanol for 1 min followed by Reynold's lead citrate for 1 min. Electron microscopy was performed on an FEI Tecnai 12 transmission electron microscope (ThermoFisher Scientific, Waltham, MA) equipped with a Gatan Ultrascan 2K CCD camera operated at 120 keV.

Three-dimensional scanning electron microscopy. Serial block-face scanning electron microscopy (SBF-SEM) was performed as described (8) with minor modifications. Colonies were manually picked and immediately fixed overnight at 4°C in 2.5% glutaraldehyde containing 2 mM calcium chloride. Subsequently, colonies were collected by centrifugation, rinsed in 0.15 M cacodylate buffer (pH 7.4) containing 2 mM calcium chloride, and post-fixed for 1 hour on ice in 0.15 M cacodylate buffer containing 2 mM calcium chloride, 1.5% potassium ferrocyanide, and 2% osmium tetroxide. Samples were rinsed in

distilled water and treated with 0.1% thiocarbohydrazide for 20 min at room temperature and rinsed in distilled water. Samples were treated with 2% osmium tetroxide for 30 min, rinsed in distilled water, dehydrated in an ethanol series, and infiltrated with Durcupan ACM resin. The sample block was mounted on an aluminum pin and trimmed to 0.5 mm × 0.5 mm. The specimen was placed in a field-emission scanning electron microscope (Zeiss Sigma VP) equipped with a serial block-face sectioning unit (Gatan 3View2XP). A backscattered electron image of the face was obtained under an accelerating voltage of 4 keV and a chamber pressure of 20 Pa in a variable pressure mode. An automatic microtome removed a 70 nm-thick slice from the face of the block and another image was recorded. Image sets (~500 slices) were collected to capture an entire cell in a colony. Individual cells in the image sets were segmented and color-rendered; three-dimensional reconstructions of the segmented single cells were derived using the Amira software (ThermoFisher Scientific). The area of vesicles containing insulin-like granules was determined using Image J (9).

Single-cell RNA sequencing. Fresh exocrine tissue was dissociated to single cells, counted, and diluted to the manufacturer's recommended concentration in 1X PBS supplemented with 0.1% BSA. An estimated 14,822 cells were captured on a 10x Chromium device using a 10X V3 Single Cell 3' Solution kit (10x Genomics, Chromium Single Cell 3' Regent kit V3 Chemistry, Cat. PN-1000092). All protocols were performed following the manufacturer's instructions. Final sequencing libraries were analyzed on a High Sensitivity DNA Chip (Agilent, Cat 5067-4626) to determine library size; final library concentrations were determined using a Qubit High Sensitivity DNA Assay Kit (Thermo). Libraries were sequenced using the paired-end setting of 101-101 with 8 cycles of index reads on an Illumina NovaSeq 6000 platform. Approximately 0.1 million reads per cell were sequenced.

Data analysis for single-cell RNA sequencing. Raw sequencing data from the biological sample was aligned to the human genome (hg19) using the CellRanger count command to produce expression data at a single-cell resolution, according to 10x Genomics. The R package Seurat (10) was used for gene and cell filtration, normalization, principal component analysis, variable gene finding, clustering analysis, and Uniform Manifold Approximation and Projection (UMAP) dimension reduction. Briefly, a matrix containing gene-by-cell expression data was imported to create individual Seurat objects for the sample. Cells with <200 detectable genes, >5,000 featured genes, and >15% mitochondrial genes were excluded, resulting in 7,812 cells. Principle component analysis of all cells identified sub-populations, which were visualized using UMAP data dimension reduction into 2D clusters. Violin plots were used to present gene expression levels of cells for each cluster. Another round of unbiased principle component analysis of the ductal cell cluster further distinguished unique sub-populations.

The R packages RaceID3 and StemID2 (11) were used to detect rare cells within the Seurat-identified ductal cluster, that correspond to outliers in conventional clustering methods. These algorithms require a gene-by-cell expression matrix as input and produce a clustering partition representing cell types. Within the ductal cluster, *SOX9*, *PDX1*, *NKX6.1* triple-positive (TP) cells were separated from the non-TP cells, which include cells that expressed 1 or 2 of these transcription factors. Differentially expressed genes between these two groups were determined using the FindAllMarkers function in Seurat. Ingenuity Pathway Analysis (Qiagen) was performed on differentially expressed genes ($P<0.05$ and no limit set for log2 fold change) among the four ductal clusters (Data S1), TP cells compared to non-TP ductal cells (Data S1), and StemID cells compared to non-StemID ductal cells (Data S1). The identified upregulated canonical pathways (with a z-score >1.1) or downregulated canonical pathways (with a z-score <-1.1) were subsequently sorted based on –log(p-value). The top 5-10 pathways are presented.

Statistical Analysis. All values are shown as mean or median ± standard error of the mean (SEM) or standard deviation (SD). Unless otherwise specified, Student's *t*-test with Welch's correction was used between two groups, and ordinary one-way ANOVA, with Dunnett's multiple comparison, was used when there were more than two groups to determine statistical significance, with $p<0.05$ considered significant.

References:

1. Qi M, Valiente L, McFadden B, Omori K, Bilbao S, Juan J, et al. The Choice of Enzyme for Human Pancreas Digestion is a Critical Factor for Increasing the Success of Islet Isolation. Transplantation direct. 2015 May;1(4). PubMed PMID: 26146662. Pubmed Central PMCID: 4486320.
2. Tremblay JR, LeBon JM, Luo A, Quijano JC, Wedeken L, Jou K, et al. In Vitro Colony Assays for Characterizing Tri-potent Progenitor Cells Isolated from the Adult Murine Pancreas. Journal of visualized experiments : JoVE. 2016 Jun 10(112). PubMed PMID: 27340914. Pubmed Central PMCID: 4927776.
3. Suda T, Suda J, Ogawa M. Single-cell origin of mouse hemopoietic colonies expressing multiple lineages in variable combinations. Proceedings of the National Academy of Sciences of the United States of America. 1983 Nov;80(21):6689-93. PubMed PMID: 6579554. Pubmed Central PMCID: 391236.
4. Quijano JC, Tremblay JR, Rawson J, Ku HT. Isolation and Characterization of Colony-Forming Progenitor Cells from Adult Pancreas. Methods in molecular biology. 2019;2029:63-80. PubMed PMID: 31273734.
5. Gerling IC, Friedman H, Greiner DL, Shultz LD, Leiter EH. Multiple low-dose streptozocin-induced diabetes in NOD-scid/scid mice in the absence of functional lymphocytes. Diabetes. 1994 Mar;43(3):433-40. PubMed PMID: 8314017.
6. Bankhead P, Loughrey MB, Fernandez JA, Dombrowski Y, McArt DG, Dunne PD, et al. QuPath: Open source software for digital pathology image analysis. Scientific reports. 2017 Dec 4;7(1):16878. PubMed PMID: 29203879. Pubmed Central PMCID: 5715110.
7. Jin L, Feng T, Shih HP, Zerda R, Luo A, Hsu J, et al. Colony-forming cells in the adult mouse pancreas are expandable in Matrigel and form endocrine/acinar colonies in laminin hydrogel. Proceedings of the National Academy of Sciences of the United States of America. 2013 Mar 5;110(10):3907-12. PubMed PMID: 23431132. Pubmed Central PMCID: 3593860.
8. West JB, Fu Z, Deerinck TJ, Mackey MR, Obayashi JT, Ellisman MH. Structure-function studies of blood and air capillaries in chicken lung using 3D electron microscopy. Respiratory physiology & neurobiology. 2010 Feb 28;170(2):202-9. PubMed PMID: 20038456. Pubmed Central PMCID: 2821748.
9. Schneider CA, Rasband WS, Eliceiri KW. NIH Image to ImageJ: 25 years of image analysis. Nature methods. 2012 Jul;9(7):671-5. PubMed PMID: 22930834. Pubmed Central PMCID: 5554542.
10. Satija R, Farrell JA, Gennert D, Schier AF, Regev A. Spatial reconstruction of single-cell gene expression data. Nature biotechnology. 2015 May;33(5):495-502. PubMed PMID: 25867923. Pubmed Central PMCID: 4430369.
11. Grun D, Muraro MJ, Boisset JC, Wiebrands K, Lyubimova A, Dharmadhikari G, et al. De Novo Prediction of Stem Cell Identity using Single-Cell Transcriptome Data. Cell stem cell. 2016 Aug 4;19(2):266-77. PubMed PMID: 27345837. Pubmed Central PMCID: 4985539.
